# Supplementary material for: GTM-decon: guided-topic modeling of single-cell transcriptomes enables sub-cell-type and disease-subtype deconvolution of bulk transcriptomes
Source: Genome Biol. 2023 Aug 18;24:190. doi: 10.1186/s13059-023-03034-4 (PMC10436670; doi:10.1186/s13059-023-03034-4)
Supplement: Supplementary file 1 — Additional file 1. Supplementary Section S1 (Evaluation on gene selection strategies); S2 (Evaluation on raw count and transformation strategies); S3 (Experimenting hyperparameters $\alpha_{\mathrm m,\mathrm k}$αm,k for cell-type mixture prior); S4 (Experimenting hyperparameter $\beta$β for CTS topics); S5 (Experimenting number of topics per cell type); S6 (Benchmark time and memory usage); S7 (Effect of sparsification on phenotype classification); S8 (Phenotype-CTS topic modeling of single-cell breast cancer transcriptomes for TCGA-BRCA bulk deconvolution); S9 (Effect of cell size on inference of cell-type deconvolution); Table S1-S3; Figure S1-S31. [file 13059_2023_3034_MOESM1_ESM.docx]

GTM-decon: guided-topic modelling of single-cell transcriptomes enables sub-cell-type and disease-subtype deconvolution of bulk transcriptomes

Additional file 1

Lakshmipuram Seshadri Swapna^1^, Michael Huang^1^, and Yue Li^1,*^

^1^School of Computer Science, McGill University, Montreal, QC, Canada

^*^Correspondence: yueli@cs.mcgill.ca

# S1. Evaluation on gene selection strategies

We assessed the effects of using different genes selection strategies and different data transformations on the deconvolution performance. Specifically, we tested the effect of using all genes (ALL), preprocessed genes (PP) and only the highly variable genes (HVG) as input set for training GTM-decon. We observe that using all genes performed as well as or better than using only the HVG (**Figure S2-S4, S6**). Pre-processed genes vary in their performance depending on the dataset. The good performance on all genes also offers the added advantage of learning the individual contributions of each gene for each cell type in efforts of discovering novel marker genes.

# S2. Evaluation on raw count and transformation strategies

We experimented two transformation methods namely normalizing read counts (normr) and scaling followed by log-transformation (normr_log1p) as detailed in **Methods**. GTM-decon that operate on raw counts without any normalization performs the best for all the gene sets (**Figure S3**).

# S3. Experimenting hyperparameters $\boldsymbol{\alpha}_{\mathbf{m,k}}$ for cell-type mixture prior

We also experimented with different values for the hyperparameter values of the cell-type topic mixture prior $\boldsymbol{\theta}_{m}\sim\mathrm{Dir}(\boldsymbol{\alpha}_{m})$. In our default setting, we set the hyperparameter $\alpha_{m,k}$ to a relatively high value (i.e., 0.9 by default) given the cell-type label $y_{m}=k$; the rest of the K-1 $\alpha_{m,k^{'}}$ values, where $y_{m}\neq k^{'}$, are set to a relatively low values (i.e., randomly sampled from a range between 0.1 and 0.01). To assess the impacts of the hyperparameter values, we varied the values for the CTS $\alpha_{m,k}$ from 0.6 to 1. We also derived a data-driven prior from a multi-class logistic regression (MCLR) model that was trained to predict cell types of each cell. In particular, we use the fitted values of the MCLR in terms of the predicted probabilities as the prior values for each topic. The fitted values for all the correct cell types are above 0.8, suggesting a high confidence of the MCLR fit. We evaluated these prior settings on two cell-sorted purified immune cell datasets, GSE107011 (114 samples) and GSE64655 (48 samples) (**Table S2**). We find that the model is largely robust to the variation in topic prior (including the prior from MCLR), for all gene sets, for all the data sets (**Figure S4**). Visualizing the results as heatmaps also shows that varying the topics does not vary the clustering pattern, while confirming that the samples are largely clustered based on their primary cell type (**Figure S5**). We further evaluate on two real bulk datasets of whole blood and immune cells with known ground truth proportions (i.e., WB and S13 cohorts listed in **Table S2**) by assessing the correlation between deconvolved cell-type proportions from GTM-decon and ground truth proportions from flow cytometry data for each sample. The results also confirm that GTM-decon is robust to the variation of topic prior values in this range (**Figure** **S6**).

# S4. Experimenting hyperparameter $\boldsymbol{\beta}$ for CTS topics

By default, we set the hyperparameter $\beta$ to 0.01, to let the scRNA-seq data likelihood drive the CTS topic distribution Φ. We vary this value between 0.0001 – 1 to evaluate its effect on the deconvolution of the whole blood dataset (WB) with known ground truth proportions, as well as on purified immune cells (**Figure S7**). The results show that GTM-decon is robust to the different values of the hyperparameter.

# S5. Experimenting number of topics per cell type

GTM-decon can infer multiple topics per cell type. While in most of our applications we find that the basic GTM-decon with one topic per cell type confers good performance, the GTM-decon with multiple topics per cell type tends to provide even better performance in terms of deconvolution accuracy on simulated bulk RNA-seq data using real scRNA-seq data (**Figure S8**).

# S6. Benchmark time and memory usage

We have benchmarked the time cost and memory usage as a function of the number of cells in the scRNA-seq reference data for GTM-decon in comparison with 3 SOTA methods, namely BISQUE, BSEQSC, and MuSiC. We did not benchmark CIBERSORTx because it is run remotely on a hosted web server. For the scRNA-seq reference, we used a combined PMBC datasets (**Table S2**) containing 30,000 cells, and 33,694 genes. Each method was trained to infer the cell type proportion in the purified bulk samples (GEO accession GSE64655). While the exact type of bulk data is not important in these time/memory benchmark experiments, we expect that each algorithm converges relatively fast due to the homogeneity of the bulk data. For GTM-decon, we used 1-5 topics per celltype (i.e., 9-45 topics in total for 9 cell types in the scRNA-seq reference data). For the other 3 methods, we used their recommended or default settings. We used the GNU time command to record the time and memory usage. GTM-decon scale linearly with both the number of topics per cell type and the number of cells (**Figure S17** top row), which is what we expected since its time and space complexity are both $O(N\times G\times K)$ for N cells, G genes, and K topics. It also compares favourably with BISQUE, BSEQ-sc, and MuSiC in terms of running time and memory usage (**Figure S17** bottom row).

# S7. Effect of sparsification on phenotype classification

The sparsification procedure was done on the bulk data only when we directly inferred gene-topic distributions from them (i.e., only when we trained GTM-decon directly on the bulk data). This only pertains to inferring *de novo* topics from the bulk data using the standard LDA and the phenotype-guided topic inference from the bulk data. We observe that sparsification improved phenotype prediction accuracy on breast cancer data (TCGA-BRCA) for predicting ER+ and Basal subtypes compared to using the original bulk data as they are. Specifically, we varied the sparsification rates on the bulk data by setting all values below the n-th percentile to be zero and evaluated the prediction accuracies as a function of the sparsification rate. We observe increasing prediction accuracies for up to 90% as we increased the sparsification rate up to 60% (**Figure S27)**.

We sparsified values not genes. As a result, the percentage of the non-zero genes (i.e., genes with non-zero values in at least one sample after the sparsification) is about 70% even at 75% sparsification, suggesting that the model still contains a sufficient number of genes for capturing the cell types and phenotype specific variations. We investigated the contribution of the zero genes (i.e., genes that were removed after sparsification due to zero value across all samples). We observe that most of the zero genes occur in the lower 50th percentile of the CTS probabilities (**Figure S28**, left panel). Additionally, most of the zero genes do not feature in the top 25th percentile of the DE genes as determined by DESeq2 on the bulk TCGA-BRCA samples at FDR < 0.05 (**Figure S28**, right panel). Although differentially expressed in bulk, sparse genes do not contribute much to cell-type specific differences (**Figure S28**, left panel), and hence may not be captured by GTM-decon, which captures cell-type specific differences in gene expression.

# S8. Phenotype-CTS topic modeling of single-cell breast cancer transcriptomes for TCGA-BRCA bulk deconvolution

We can also infer both phenotype and CTS topics from the same single-cell breast cancer transcriptomes, which are composed of labeled cells from patients with 11 breast cancer patients of ER+ subtype and 10 patients with triple negative breast cancer (TNBC) subtype. From this dataset, we inferred CTS-phenotype topics simultaneously guided by the cancer subtypes (i.e., ER+ and TNBC) and cell types. We then a) applied the GTM-decon to deconvolve bulk TCGA-BRCA RNA-seq samples into CTS and phenotype-specific profiles; b) identified CTS-specific DE genes between ER+ and TNBC from the single-cell data; c) used the genes-by-CTS topics matrix to visualize the CTS distribution of DE genes identified from the bulk data.

Briefly, we modeled the phenotype-guided and cell-type-guided topic model by assigning one topic to each cell type per phenotype. During training, the topic corresponding to that cell type of that phenotype is set to a prior value of 0.9, and the rest of the topics (for the other cell types or phenotype) were set at a random value between 0.01 and 0.1 (**Figure S30a**). These priors guide the topic model to learn phenotype-CTS distribution. All the genes in the experiment were used as features.

We described the results presented in **Figure S30b-d** as follows:

1. The trained GTM-decon was evaluated by its classification accuracy in discriminating ER+ phenotypes from Basal-like phenotypes (the closest subtype to the TNBC subtype from the TCGA-BRCA data) in bulk RNA-seq data from TCGA-BRCA (n=1212). The left panel in **Figure S30b** illustrates that the samples corresponding to ER+ subtype are enriched for the ER+ topic. Interestingly, the samples corresponding to Basal-like subtype exhibit 2 different clusters - a cluster of samples showing enrichment for the TNBC topic (enclosed in green rectangle), and another cluster showing similar distributions of ER+ and TNBC topics (enclosed in brown rectangle). The former cluster likely corresponds to TNBC samples clustered into the Basal-like category, and the latter to others in the Basal-like category.
2. We deconvolved the TCGA-BRCA bulk samples without sparsification, resulting in a remarkably high phenotype classification accuracy of 92% using all genes, 90% using pre-processed genes, and 93% using highly variable genes. These results indicate that phenotype-guided topic modeling using scRNA-seq data can be effective in deconvolving bulk RNA-seq data as they are.
3. Furthermore, the CTS topics inferred for each phenotype enable us to visualize the deconvolution per sample at the CTS resolution for ER+ and TNBC/Basal-like subtypes (**Figure S30b**, right panel). We observe the enrichment of Cancer-Basal topic for TNBC (enclosed in orange rectangles), and enrichment of LumA and LumB topics for ER+ samples, as expected (enclosed in blue rectangles), and in concordance with the results obtained from cell-type guided topic modeling before (**Figure S23**).
4. Using the single-cell breast cancer transcriptomes alone, we identified CTS DE genes between the ER+ and TNBC subtypes based on the phenotypic differences in terms of the CTS probabilities per gene (**Figure S30c**). Based on 100,000 permutation tests of randomly shuffling the phenotype labels across cells, we identified 4,838 DE genes for at least one cell type at the empirical p-value < 0.05, 1,687 of which overlapped with the 5,936 DE genes identified from the TCGA-BRCA bulk RNA-seq dataset using DESeq2 at FDR < 0.05 for testing 20,501 protein-coding genes in total. Therefore, the overlap is statistically significant at p-value = 6.86E-25 based on Hypergeometric test. Similarly, as a baseline, we performed 100,000 permutation tests on the average CTS gene expression values derived from the single-cell data to compare the CTS gene expression difference between ER+ and basal-like subtypes. We identified 1892 DE genes for at least one cell type at the empirical p-value < 0.05, 592 of which overlapped with the 5,936 DE genes identified from the TCGA-BRCA bulk RNA-seq dataset using DESeq2 at FDR < 0.05 for testing 20,501 protein-coding genes in total. Therefore, the overlap is not as statistically significant as the above result at p-value = 0.01041539 based on Hypergeometric test.
5. We then visualized the expression difference of the DE genes from the bulk data in terms of the CTS-topic probabilities difference between ER+ and TNBC (**Figure S30d** left heatmap). We observe two distinct clusters of genes as highlighted in the orange and blue boxes, the former down-regulated in the ER+ phenotype topics (and up-regulated in TNBC), and the latter down-regulated in the TNBC phenotype topics (and up-regulated in ER+).
   1. The cluster of genes upregulated in TNBC phenotype (enclosed in orange rectangle) consists of ~1200 genes, predominantly upregulated in the Cancer-Basal, and Cancer-Cycling cell types. Based on over-representation analysis by ClusterProfiler on the MSigDB database, we found that these genes are enriched for E2F_TARGETS, G2M_CHECKPOINT, MYC_TARGETS_V2, and MITOTIC_SPINDLE pathways at FDR < 1.71E-18, 4.71E-11, 2.37E-05, and 1.07E-02, respectively, with an overlap of 58, 46, 17 and 29 genes. For the E2F_TARGETS gene set, some of the DE genes are MCM4, SYNCRIP, STMN1, TK1, SSRP1, CKS1B, EED, HELLS, USP1, and for the MYC_TARGETS_V2 set, some of the DE genes are MCM4, SRM, NDUFAF4, WDR43, WDR74, PLK1, PUS1, NOP2, RCL1, NIP7, SUPV3L1, MRTO4. The associations of these genes with the TNBC phenotype are supported by the literature. For instance, MYC_TARGETS_V1 and MYC_TARGETS_V2 are associated with tumor aggressiveness and poor survival in aggressive subtypes such as TNBC and HER2-positive breast cancer, when compared with ER-positive/HER2-negative tumors (MYC v1 and v2, both *p* < 0.001) [98]. Also, the genes encoding cell-cycle-related targets of E2F transcription factors (E2F_TARGETS) are associated with aggressive clinical characteristics, such as TNBC [99]. Higher G2M cell cycle pathway activity has been shown to be associated with worse clinico-pathologic features, with the TNBC subtype exhibiting the highest activity [100].
   2. The cluster of genes up-regulated in ER+ cancer subtype (enclosed in blue rectangle) consists of ~1000 genes, predominantly upregulated in the Cancer-LumA, Cancer-LumB, Cancer-Her2 cell types. These genes are over-represented in the Estrogen Response (Early and Late) pathways, with an overlap of 57 and 48 genes, at an FDR of 2.84E-25 and 9.54E-18, respectively. As the name suggests, these pathways correspond to genes up-regulated in response to Estrogen receptor activation, from which the ER+ subtype derives its name. Interestingly, 20 genes involved in Bile Acid Metabolism are also up-regulated at an FDR of 7.34E-03, which may be relevant as elevated bile metabolism is associated with better survival in breast cancer, with ER+ subtype exhibiting the highest bile acid metabolism in comparison to other subtypes [101].
   3. Finally, we visualized the average gene expression in another heatmap (**Figure S30d** right heatmap), which exhibits a similar but much less salient pattern compared to the heatmap derived from the CTS-topic probabilities (**Figure S30d** left heatmap).

Together, these results further demonstrate the utility of GTM-decon in guiding phenotype and cell-type topic inference to identify disease-associated gene modules.

# S9. Effect of cell size on inference of cell-type deconvolution

Algorithmically, GTM-decon estimates the RNA fractions per cell type instead of cell fractions. Therefore, differences in cell sizes may lead to divergences between RNA fractions and cell fractions. Using the Pancreas reference data as an example, while we observe differences in the average RNA counts per cell among the seven cell types (**Figure S31a**), the relative proportion of RNA counts are quite similar to the relative proportion of the cells (**Figure S31b**). More generally, using the four scRNA-seq reference datasets that were used for the deconvolution of real bulk, we observed strong correlations between the total cell fraction and total RNA fraction associated with each given cell-type (**Figure S31c-f**). This suggests that RNA fractions per cell type can serve as a good surrogate to the cell fraction per cell type despite the potential differences in cell sizes among cell types (i.e., different average RNA counts among cell types).

# Supplementary Tables

## Table S1: scRNA-seq datasets used as reference datasets for training​

| **Dataset​** | **Tissue​** | **Sample size** | **Number of cells​** | **Source​** | **Reference​** | **Data Reference** |
| --- | --- | --- | --- | --- | --- | --- |
| **Segerstolpe​** | Pancreas​ | 10​ | 2209​ | E-MTAB-5061​ | Segerstolpe et al, Cell Metab, 2016​ [85] | [69] |
| **Baron – Human​** | Pancreas​ | 4​ | 8569​ | GSE84133​ | Baron et al, Cell Systems, 2016​ [86] | [71] |
| **Baron – Mouse​** | Pancreas​ | 2​ | 1886​ | GSE84133​ | Baron et al, Cell Systems, 2016​ [86] | [71] |
| **Pancreas – Cancer​** | Pancreas​ | 35​ | 57530​ | CRA001160​ | Peng et al, Cell Res, 2019​ [87] | [72] |
| **PBMC2​** | PBMC​ | 1​ | 11183​ | Single Cell Portal / GSE132044​ | Ding et al, Nat Biotech, 2020​ [88] | [75] |
| **HBC​** | Blood​ | 31​ | 7643​ | GSE149938​ | Xie et al, Natl Sci Rev, 2021​ [89] | [76] |
| **Lake_Human​** | Brain​ | 1​ | 10319​ | GSE97930​ | Lake et al, Science, 2016 [89]​ | [77] |
| **Breast - Normal​** | Breast​ | 4​ | 24646​ | GSE113197​ | Nguyen et al, Nat Comm, 2018​ [90] | [73] |
| **Breast - Cancer​** | Breast​ | 26​ | 1000064​ | GSE176078 ​ | Wu et al, Nat Genet., 2021 ​[91] | [74] |

## Table S2: Bulk RNA-seq datasets with ground truth proportions for evaluation

​

| **Dataset​** | **Tissue​** | **Samples​** | **Data Type​** | **Source​** | **Reference​** | **Data reference** |
| --- | --- | --- | --- | --- | --- | --- |
| **Whole Blood (WB)​** | PBMC​ | 12​ | Bulk RNA-seq​ | CIBERSORTx – Fig2b​ | Steen et.al, Stem Cell Tran. Net, 2020​ [93] | [93] |
| **PBMC S13 cohort​** | PBMC​ | 11​ | Bulk RNA-seq​ | GSE107011 ​ | Monoco et. al. Cell Rep, 2019 ​[94] | [80] |
| **SDY67​** | PBMC​ | 346​ | Bulk RNA-seq​ | Immport SDY67​ | Zimmerman et. al. Front Immunol, 2017 ​[95] | [81] |
| **PBMC - GSE107011​** | Purified immune cells​ | 127​ | Purified cell-sorted Bulk RNA-seq​ | GSE107011​ | Monoco et. al. Cell Rep, 2019​ [94] | [80] |
| **PBMC - GSE64655​** | Purified immune cells​ | 56​ | Purified cell-sorted Bulk RNA-seq​ | GSE64655​ | Hoek et. al., PLoS One, 2015​ [96] | [82] |
| **ROSMAP​** | Brain​ | 41​ | Bulk RNA-seq​ | CortexCellDeconv tutorial​ | Patrick et. al., PLOS Comp. Biol, 2020​ [97] |  |
| **Segerstolpe​** | Pancreas​ | 7​ | Bulk RNA-seq​ | E-MTAB-5060​ | Segerstolpe et al, Cell Metab, 2016​ ​[85] | [70] |

The ground truth proportions are available from the supplementary materials or web links of the references. Monaco et. al. contains ground truth proportions of PBMC S13 cohort and SDY67.​

## Table S3: Percentage of preprocessed (PP) genes and HVG for scRNA-seq datasets​

| **Dataset​** | **Total genes​** | **PP genes​** | **HVG​** | **%PP​** | **%HVG​** |
| --- | --- | --- | --- | --- | --- |
| **Segerstolpe​** | 26178​ | 17486​ | 5027​ | 66.8​ | 19.2​ |
| **Baron​** | 20130​ | 15502​ | 1667​ | 77​ | 8.28​ |
| **Breast – Normal​** | 33694​ | 20721​ | 2060​ | 61.5​ | 6.11​ |
| **Breast - Cancer​** | 29736​ | 26169​ | 2355​ | 88​ | 7.92​ |
| **PBMC2​** | 33693​ | 20830​ | 8225​ | 61.8​ | 24.4​ |
| **HBC​** | 19815​ | 17577​ | 4389​ | 88.7​ | 22.1​ |

​

# Supplementary Figures


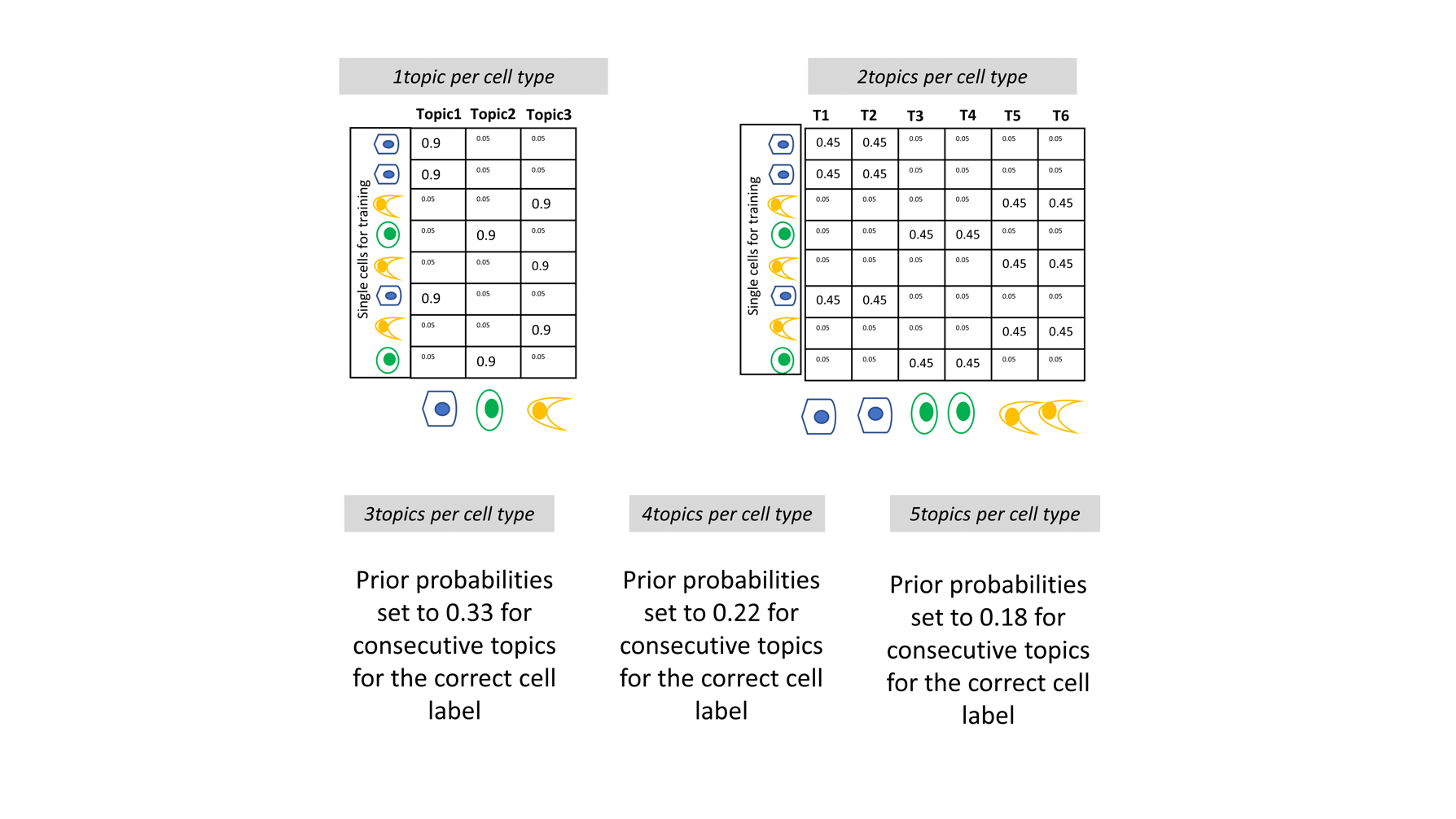


## Figure S1: Modeling a cell type using multiple topics. GTM-decon can model each cell type via multiple topics (2, 3, 4, 5). It achieves this by setting the prior values to be assigned to the hyperparameter $\boldsymbol{\alpha}_{\boldsymbol{m,k}}$ for each of cell of cell type *k* to 0.45 , 0.33, 0.22, and 0.18, respectively, for ‘x’ consecutive topics. For example, a 2-topic model with 3 cell types, as shown above, is modelled using 6 topics, with each set of 2 adjacent topics modeling a cell type. Here, the 2 adjacent topics modeling the cell type corresponding to cell ‘m’ are set to 0.45 and the remaining topics were assigned random probabilities between 0.001 and 0.01.


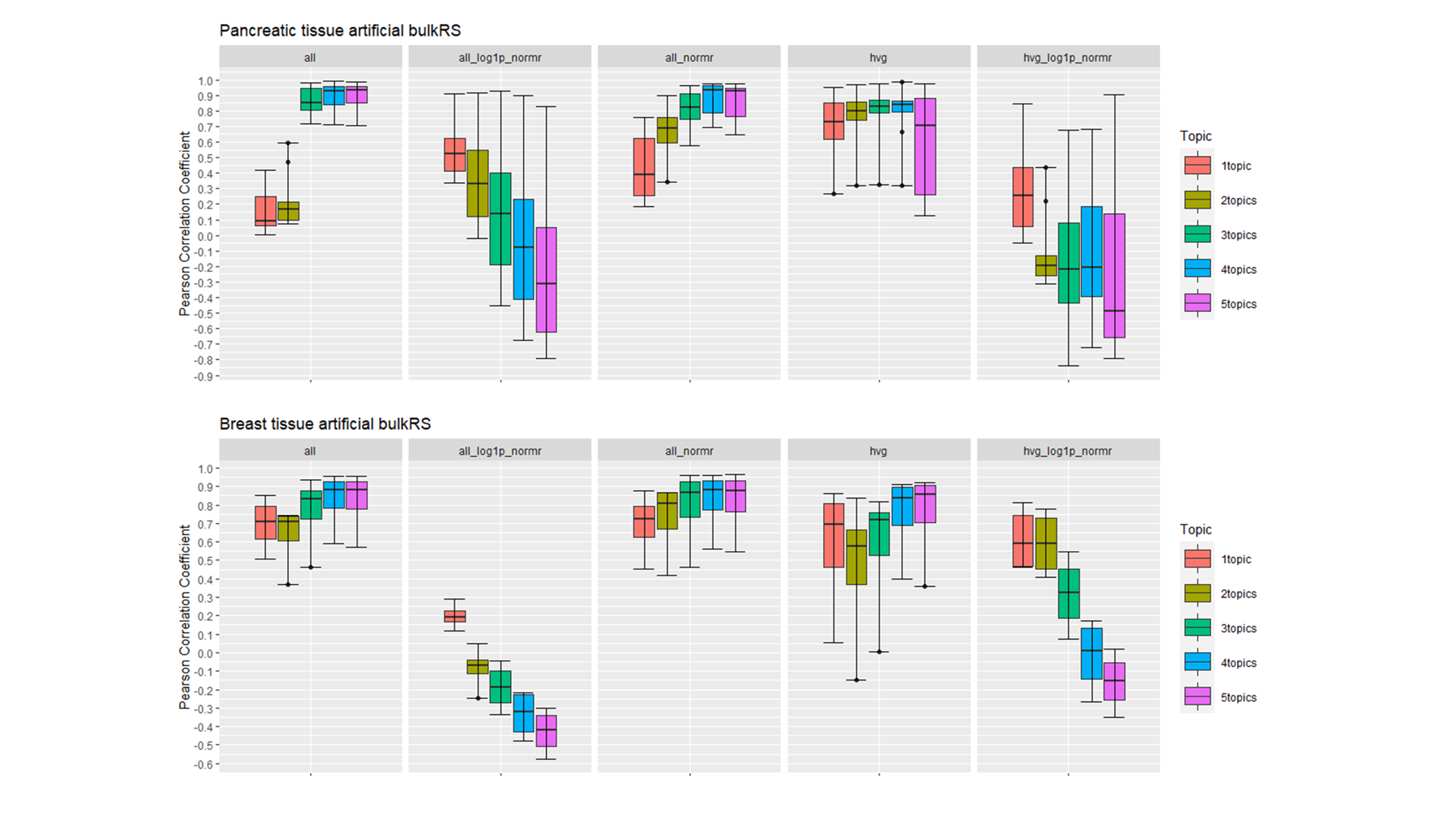


## Figure S2: Deconvolution accuracy for different gene sets from simulated data. Each GTM-decon model was trained by modelling a cell type using different gene selection strategies and their transformations i) all genes – corresponding to raw gene counts; ii) all_normr – corresponding to normalized of raw genes by total counts in a cell; iii) all_log1p_normr – corresponding to log-transformed form of all_normr; iv) hvg – corresponding to raw counts of highly variable genes; v) hvg_log1p_normr – corresponding to log-transformed form of normalized hvg. Each of these models was further trained based on K number of topics, with K varying from 1 to 5. We evaluated the deconvolution accuracy of these models on simulated bulk data from two datasets: Pancreatic (Segerstolpe) and Breast (Normal). We simulated the bulk RNA-seq (bulkRS) data by randomly sampling cells from the held-out set of scRNA-seq data (20%) and summing up the values for each gene from all cells belonging to an individual. The cell proportions inferred for each individual was compared with the ground truth values and evaluated by Pearson correlation. The box and the whiskers in each boxplot indicate the 25%-75% quartile and min-max of the evaluation scores over the individuals, respectively. ​


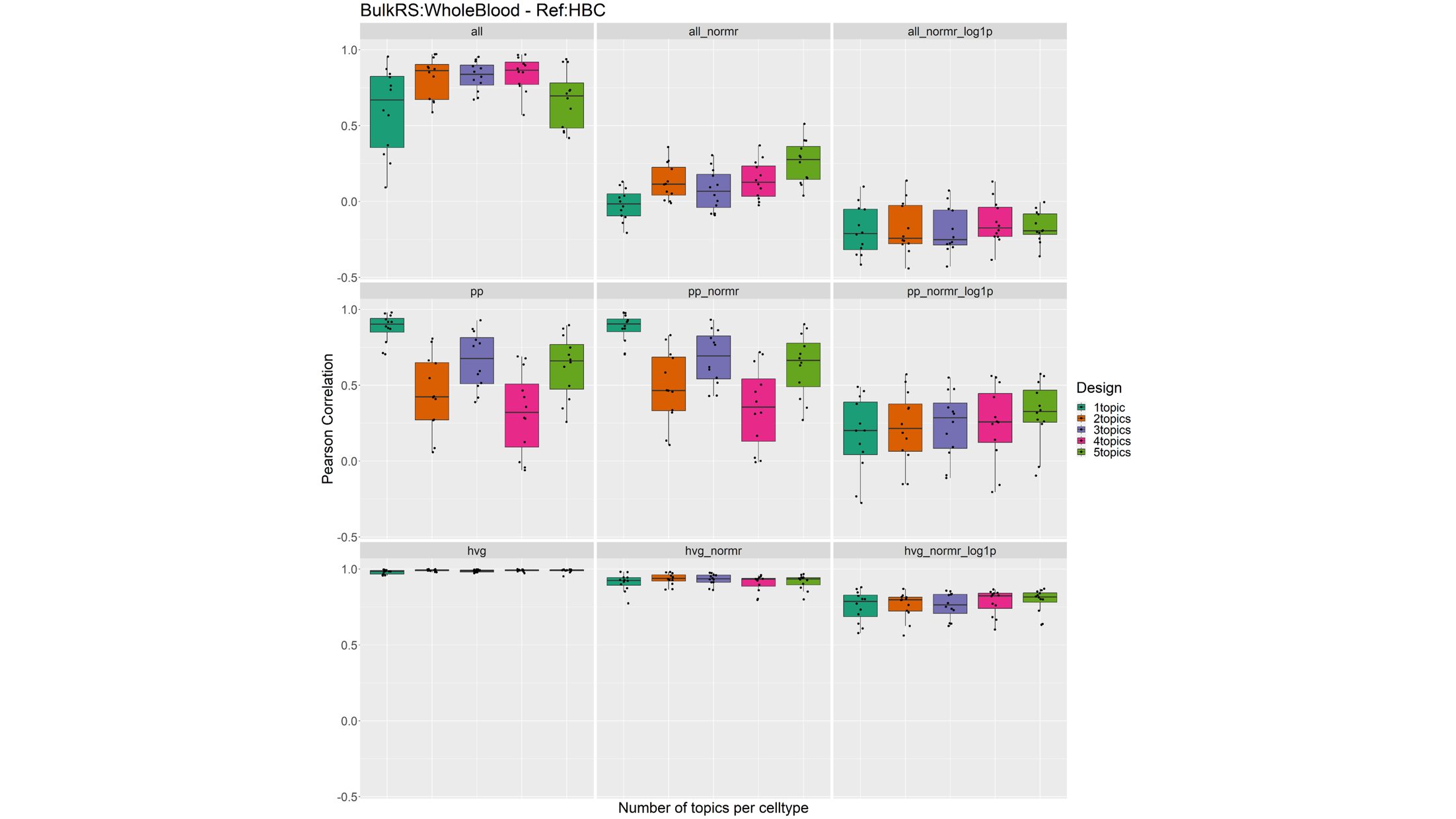


## Figure S3: Deconvolution using different normalization strategies for different gene sets: We evaluate the performance of different gene sets and their normalizations on Whole Blood RNA-seq bulk dataset. The boxplots show the deconvolution accuracy in terms of Pearson Correlation between ground truth cell type proportions estimated by flow cytometry for each sample vs. deconvolved cell type proportions inferred by GTM-decon (y-axis). The box and the whiskers in each boxplot indicate the 25%-75% quartile and min-max of the evaluation scores for each of the samples. Deconvolution performance is shown for different models using a varying number of topics (1 to 5) to model each cell type. The box plots reveal that raw counts (i.e., no normalization) performs the best for all the gene sets, whereas normalization decreases the performance to varying degrees for different gene selection strategies.​


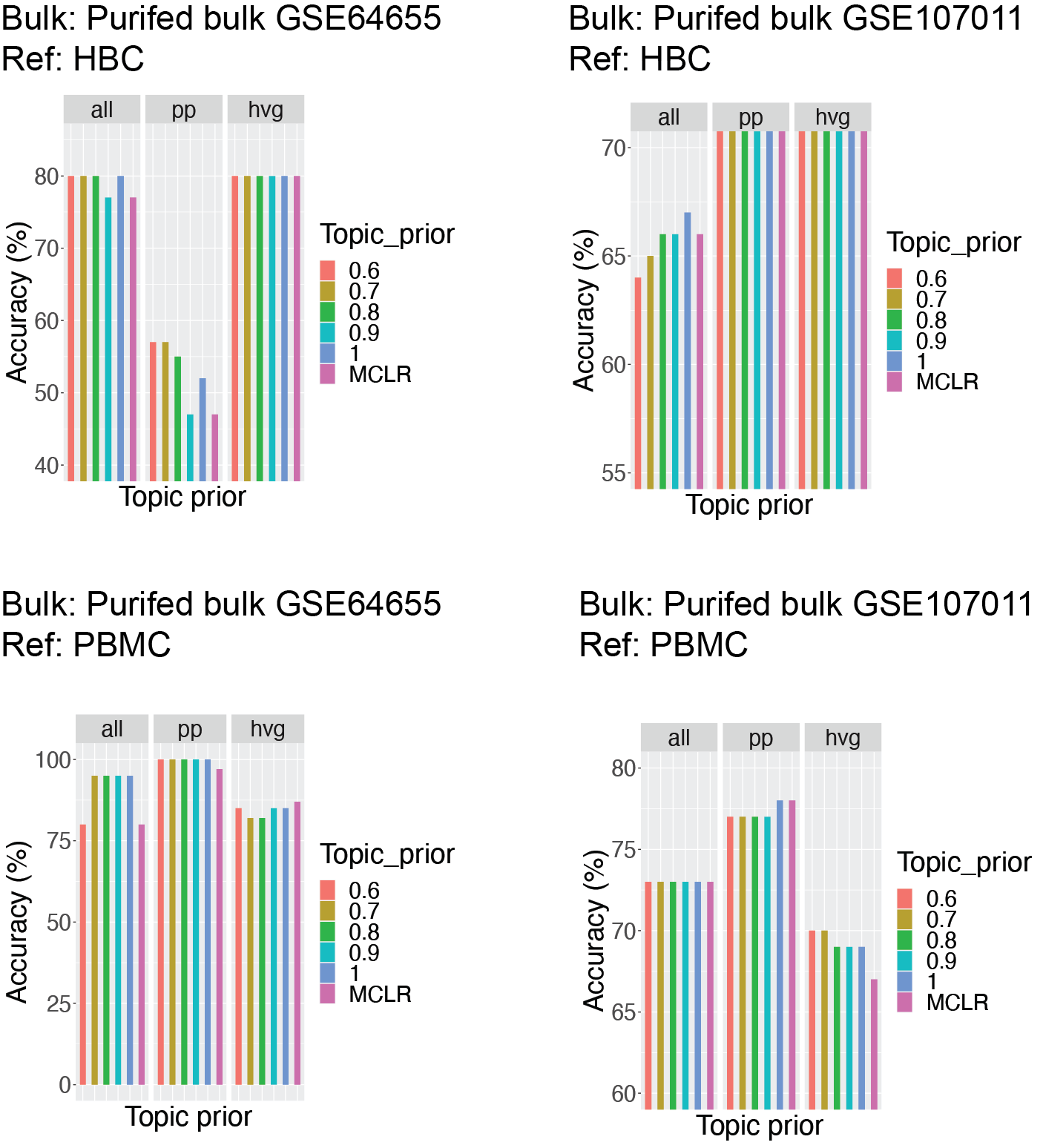


## Figure S4: Effect of varying CTS topic prior on deconvolution accuracy in purified immune bulk RNA-seq samples. We evaluated the performance of GTM-decon on GSE107011 and GSE64655 using two reference datasets, HBC and PBMC2. For each purified bulk sample, the cell type corresponding to the highest inferred cell-type proportion by each method was used as the predicted cell type. The barplots show the prediction accuracy as the percentage of the correctly predicted samples. We varied topic prior hyperparameter $\boldsymbol{\alpha}_{\boldsymbol{m,k}}$ from 0.6 to 1, as well as topic priors derived from a multi-class logistic regression model fitting the scRNA-seq data to its cell types (MCLR). Three different gene selections namely all genes (ALL), preprocessed genes (PP), and highly variable genes (HVG) were experimented. The bar plots reveal that the method is largely robust to variation in topic prior.​


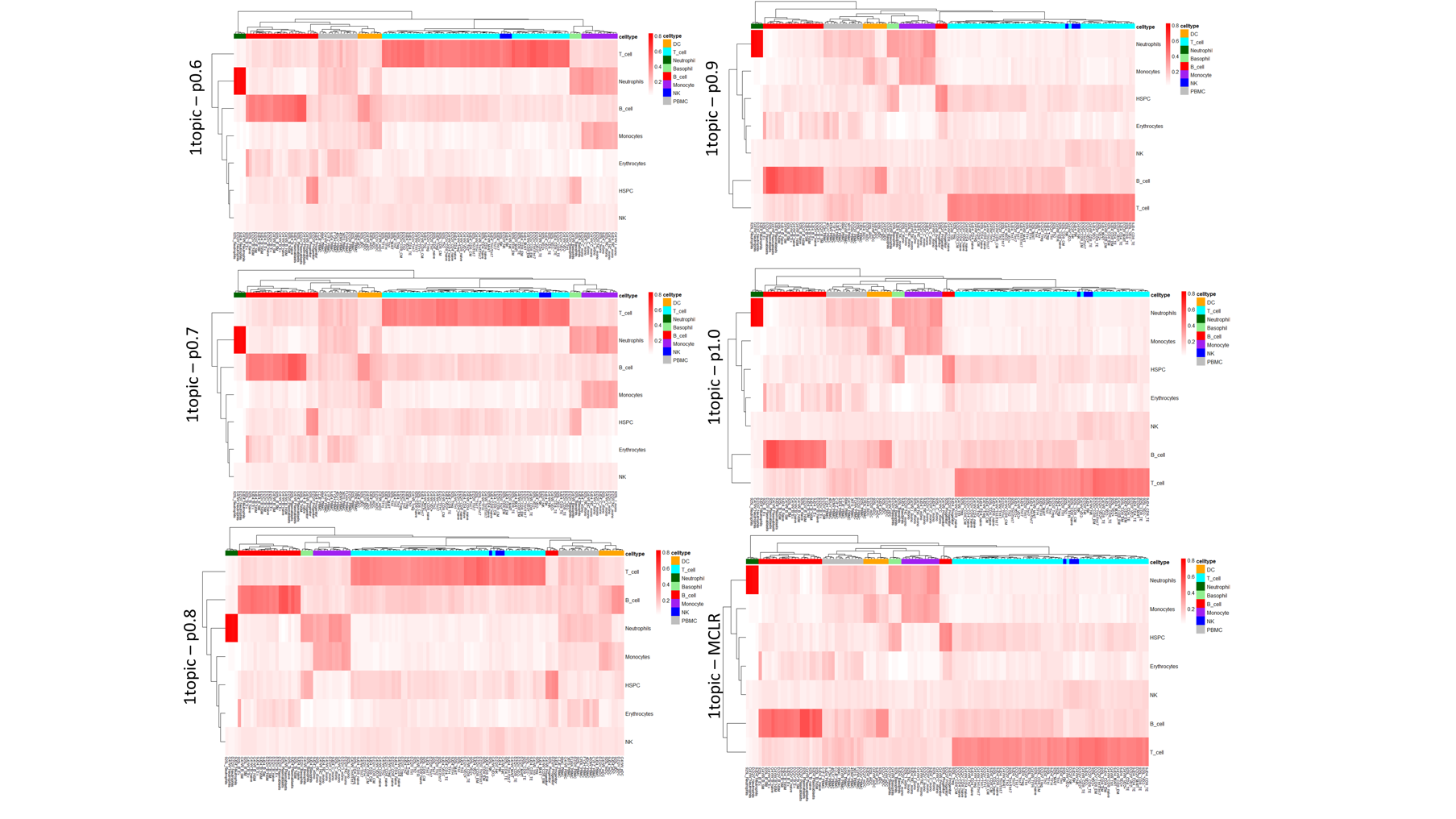


## Figure S5: Heatmaps showing deconvolved cell type proportions when CTS topic prior is varied. The heatmaps plot the deconvolved cell type proportions from GTM-decon models using different CTS topic prior values, including prior values derived from a multi-class logistic regression model (1topic-MCLR), respectively. The rows correspond to CTS topics and the columns represent bulk RNA-seq samples derived largely from purified cell-sorted immune cells (GSE107011), except for the PBMC samples. The columns are colored based on the primary cell type, and shows that in most cases, the CTS topic with the highest deconvolved proportions corresponds to the correct cell type for the sample. The heatmaps for the different priors are also largely comparable, suggesting the robustness of the approach to different priors.​


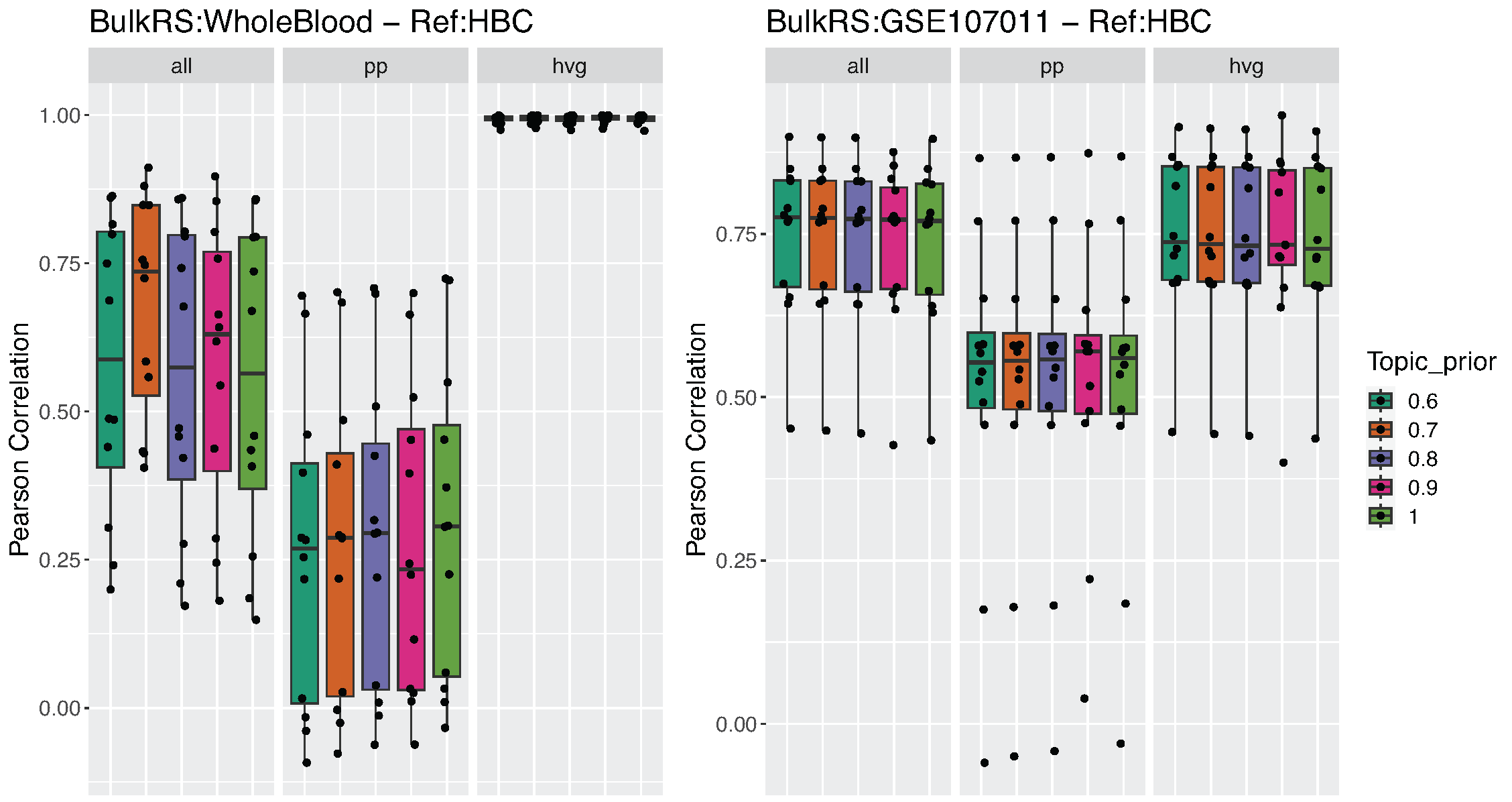


## Figure S6: Effect of varying CTS topic prior on deconvolution accuracy in two bulk RNA-seq datasets with ground truth proportions. We evaluated performance of GTM-decon on Whole Blood (WB) and GSE107011 datasets, while varying the CTS topic prior from 0.6 - 1. The boxplots show the deconvolution accuracy in terms of Pearson Correlation between ground truth cell type proportions estimated by flow cytometry for each sample vs. deconvolved cell type proportions inferred by GTM-decon (y-axis). The box and the whiskers in each boxplot indicate the 25%-75% quartile and min-max of the evaluation scores for each of the samples. The bar plots reveal that the method is largely robust to variation in topic prior, with all genes and highly variable genes performing better than pre-processed genes.​


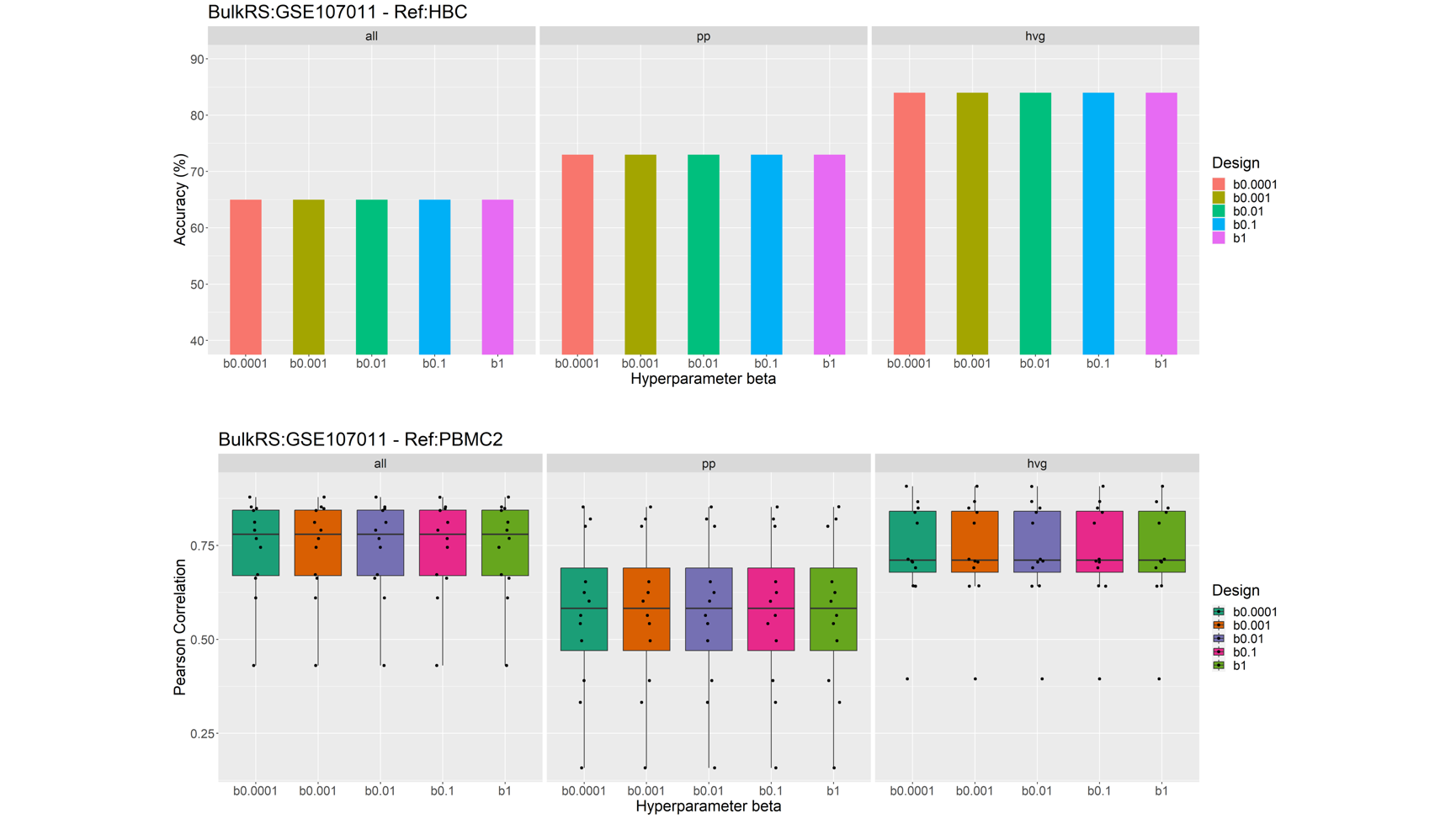


## Figure S7. Effect of varying hyperparameter $\boldsymbol{\beta}$ on deconvolution accuracy. Deconvolution accuracy was estimated in purified bulk samples (GSE107011) using HBC as the reference, and the real bulk data PBMC S13 cohort from the same dataset. The hyperparameter $\boldsymbol{\beta}$ was varied from 0.0001 to 1. For each purified bulk sample, the cell type corresponding to the highest inferred cell-type proportion by each method was used as the predicted cell type. The barplots show the prediction accuracy as the percentage of the correctly predicted samples. Similarly, deconvolution accuracy on real bulk RNA-seq (GSE107011, S13 cohort) was estimated n terms of Pearson Correlation between ground truth cell type proportions estimated by flow cytometry for each sample vs. deconvolved cell type proportions inferred by GTM-decon (y-axis). The plots reveal that the method is robust to variations in hyperparameter $\boldsymbol{\beta}$.


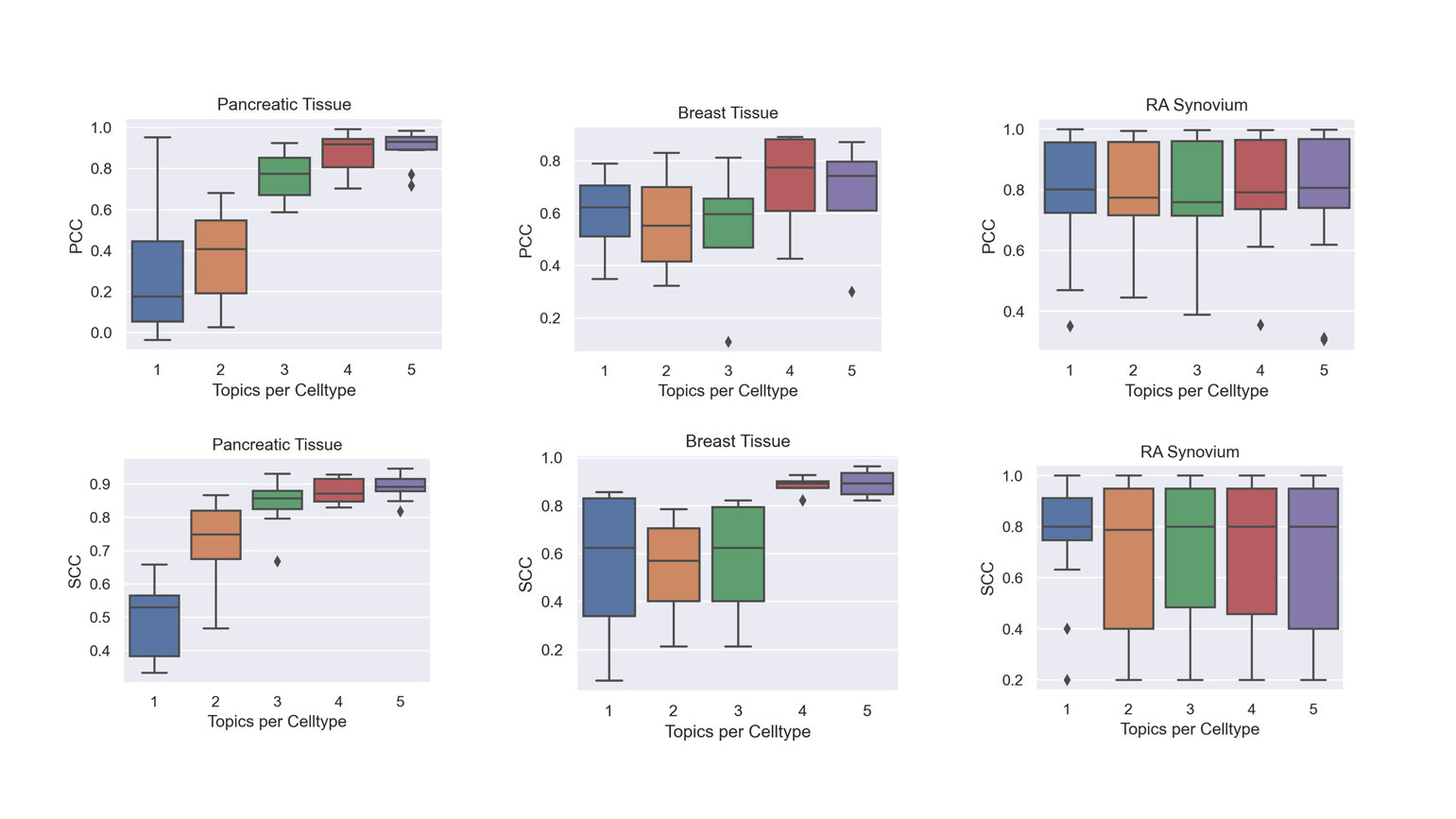


## Figure S8: Deconvolution accuracy for different topics per cell type. Each GTM-decon model was trained by modelling a cell type using K number of topics, with K varying from 1 to 5, using all genes. We evaluated the deconvolution accuracy of these models on simulated bulk data from three datasets: Pancreatic (Segerstolpe), Breast (Normal), and RA Synovium. We simulated the bulk data by summing up counts per gene for all cells per individual. For unbiased evaluation, we adopted a leave-one-out cross-validation (LOOCV) design. Each model was trained on N-1 individuals and used to deconvolve the simulated bulk transcriptome from the held-out individual for validation. The inferred cell proportions by each model were evaluated by Pearson correlation coefficient (PCC) (top row) and Spearman correlation coefficient (PCC) (bottom row). The box and the whiskers in each boxplot indicate the 25%-75% quartile and min-max of the evaluation scores over the LOOCV individuals, respectively.


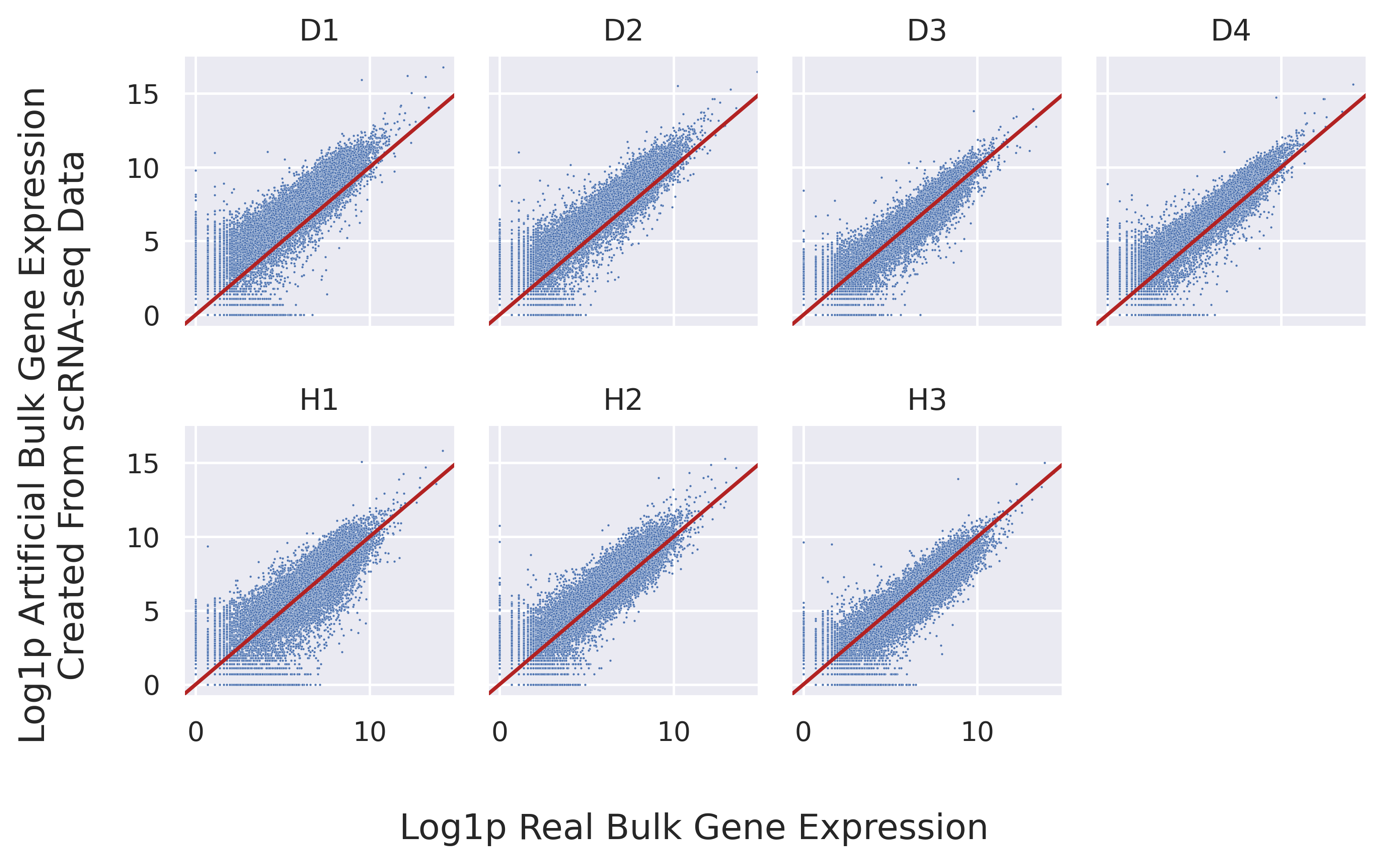


## Figure S9: Correlation between real bulk gene expression and log1p artificial bulk gene expression. The scatter plots show the correlation between log1p normalized values of real bulk gene expression (x-axis) versus log1p normalized values of artificially constructed bulk gene expression values (y-axis) for each gene. The plots are shown for each of the individuals in the Segerstolpe datasets E-MTAB5061 for scRNA-seq, and E-MTAB5060 for bulk RNA-seq, respectively. H1, H2, H3 denote samples from healthy individuals and D1, D2, D3, D4 denote samples from diabetic individuals.


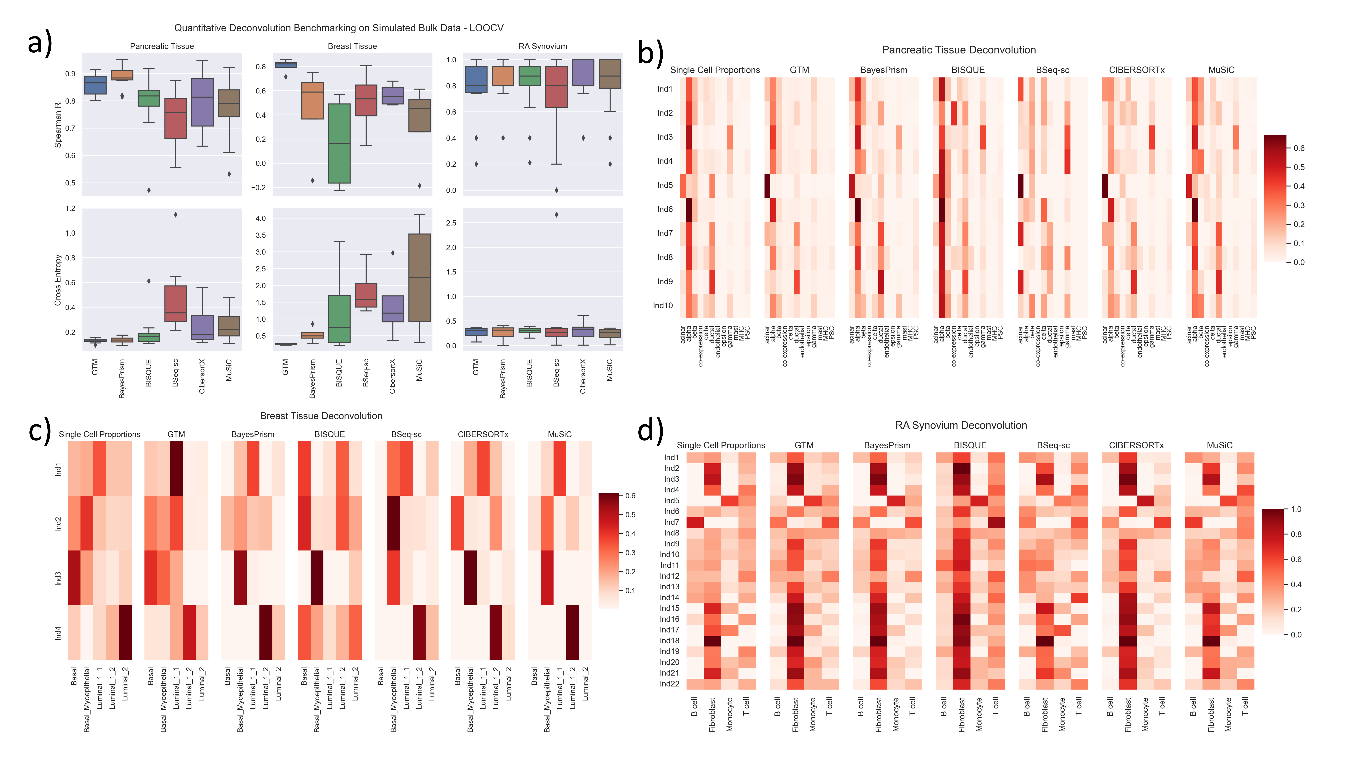


## Figure S10: Evaluation of deconvolution accuracy of simulated bulk data. a) Evaluation of cell-type deconvolution accuracy. We simulated the bulk data by summing up counts per gene for all cells per individual. For unbiased evaluation, we adopted a leave-one-out cross-validation (LOOCV) design. Each deconvolution method was trained on N-1 individuals and deconvolved the bulk transcriptome simulated using the scRNA-seq data from the held-out individual for validation. The inferred cell proportions by each method were evaluated by Spearman correlation and Cross Entropy. The box and the whiskers in each boxplot indicate the 25%-75% quartile and min-max of the evaluation scores over the LOOCV individuals, respectively. b-d) Ground truth and Inferred cell-type proportions by each method on Pancreatic, Breast and RA Synovium Tissues simulated bulk data, respectively. In each heatmap, the rows are individuals, and the columns are cell types. The color intensity is proportional to the inferred cell-type proportions.


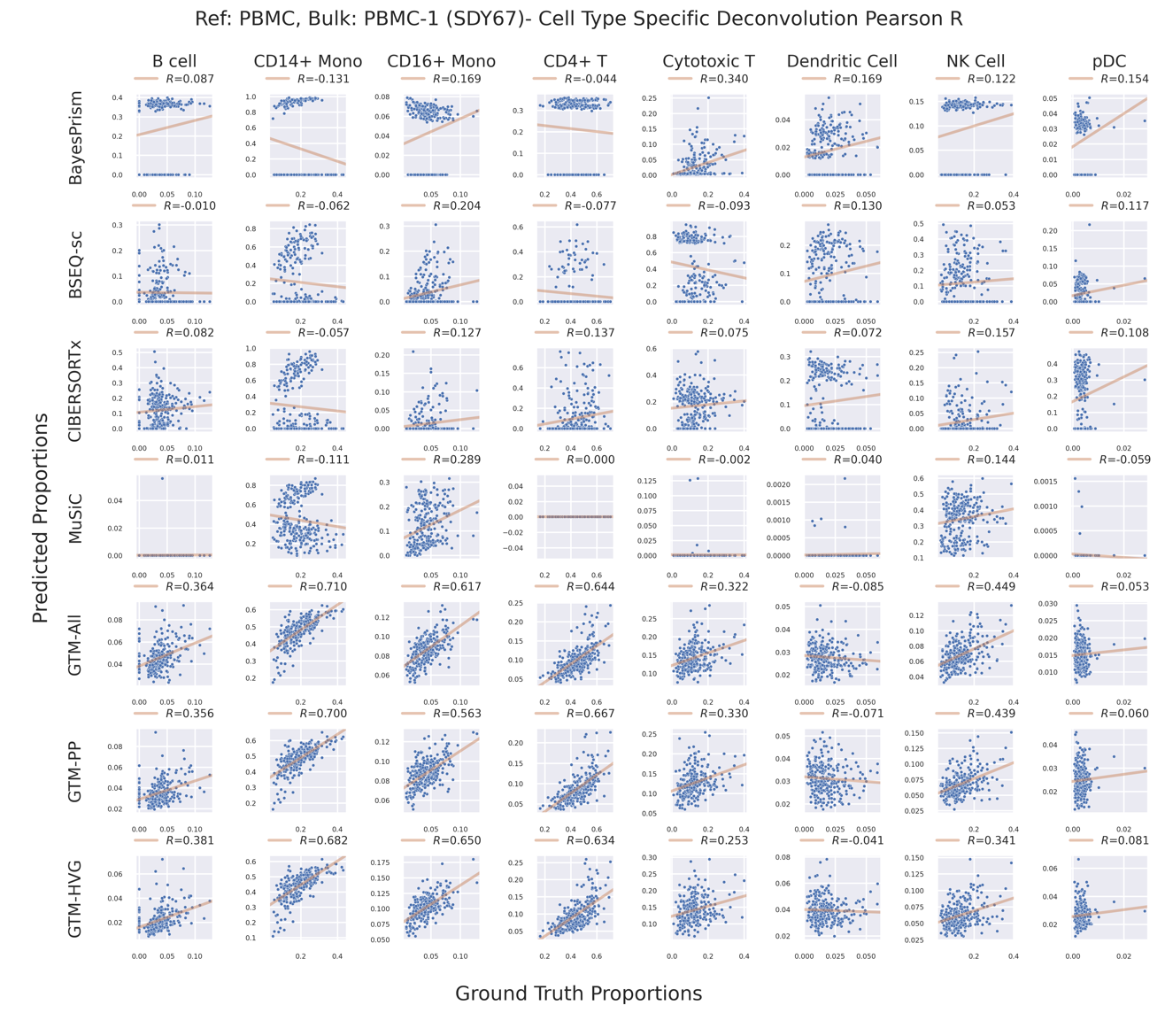


## Figure S11: Scatterplot of inferred cell-type proportion and ground-truth true proportion of real bulk data from PBMC-1 (SDY67 in Table S2) based on a separate scRNA-seq reference of PBMC2.


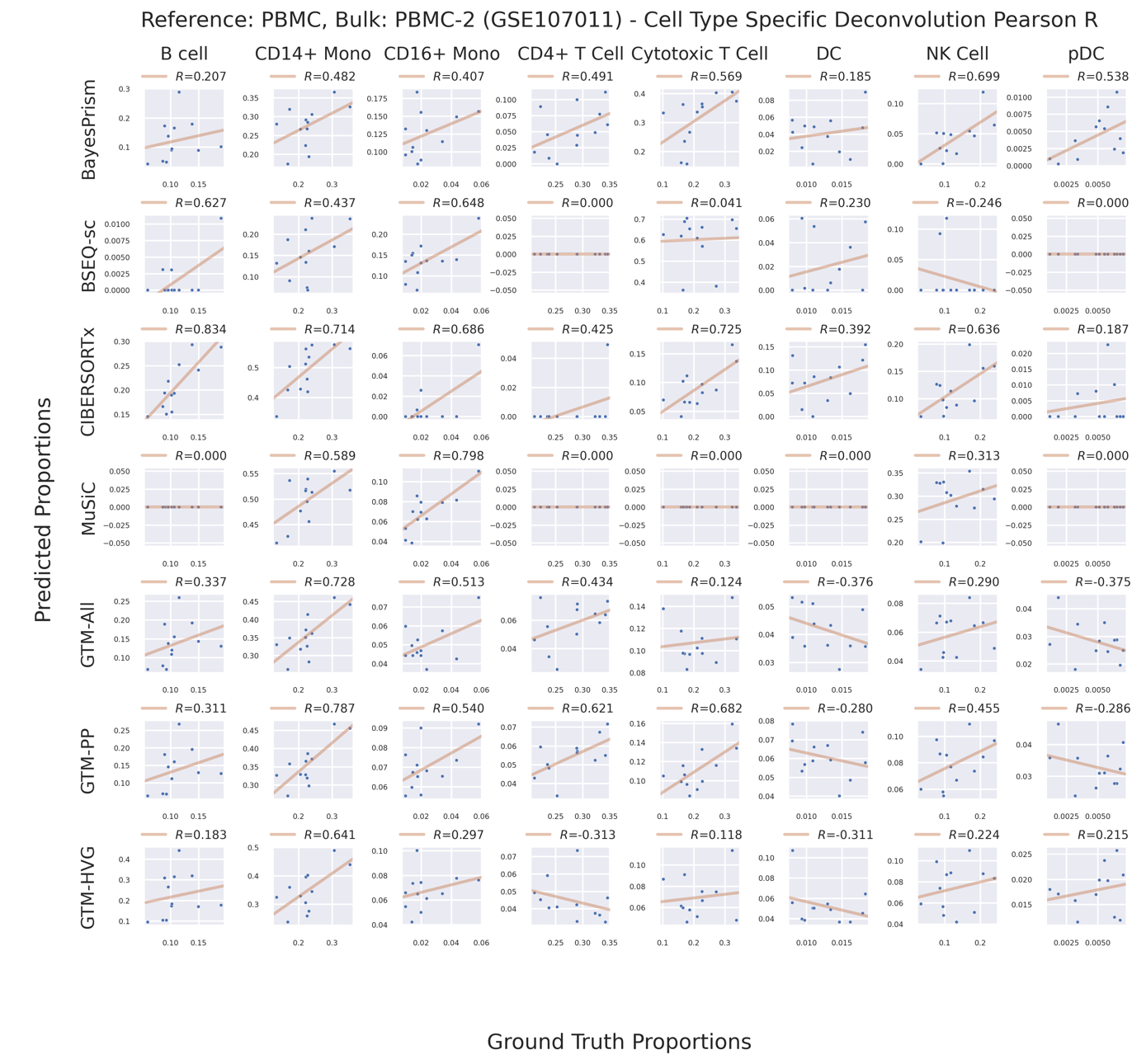


## Figure S12: Scatterplot of inferred cell-type proportion and ground-truth true proportion of real bulk data from PBMC-2 (GSE107011 in Table S2) based on a separate scRNA-seq reference of PBMC2 (Table S1).


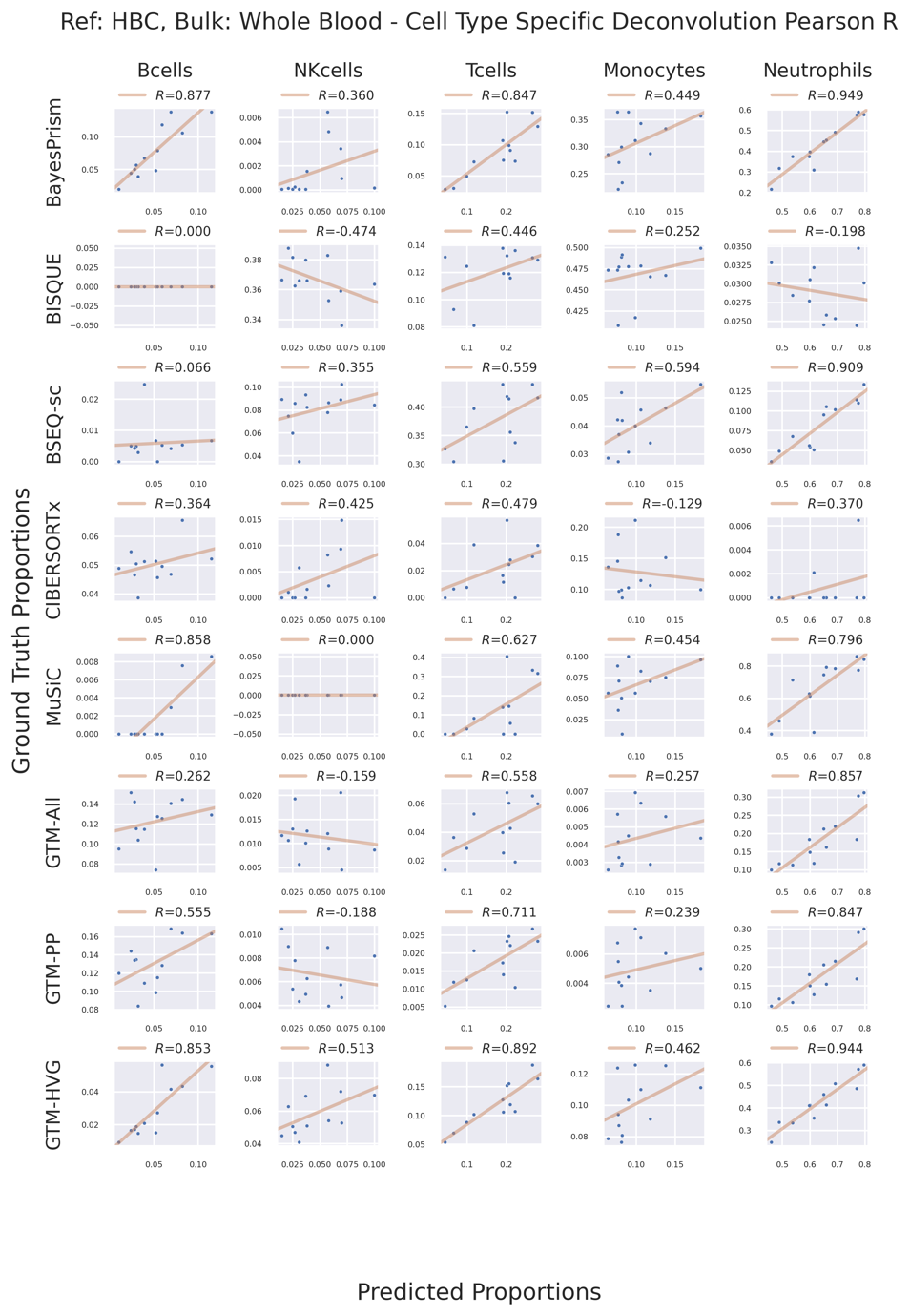


## Figure S13: Scatterplot of inferred cell-type proportion and ground-truth true proportion of real bulk data from Whole Blood (WB in Table S2) based on a separate scRNA-seq reference of Human Blood Cell (HBC in Table S1).


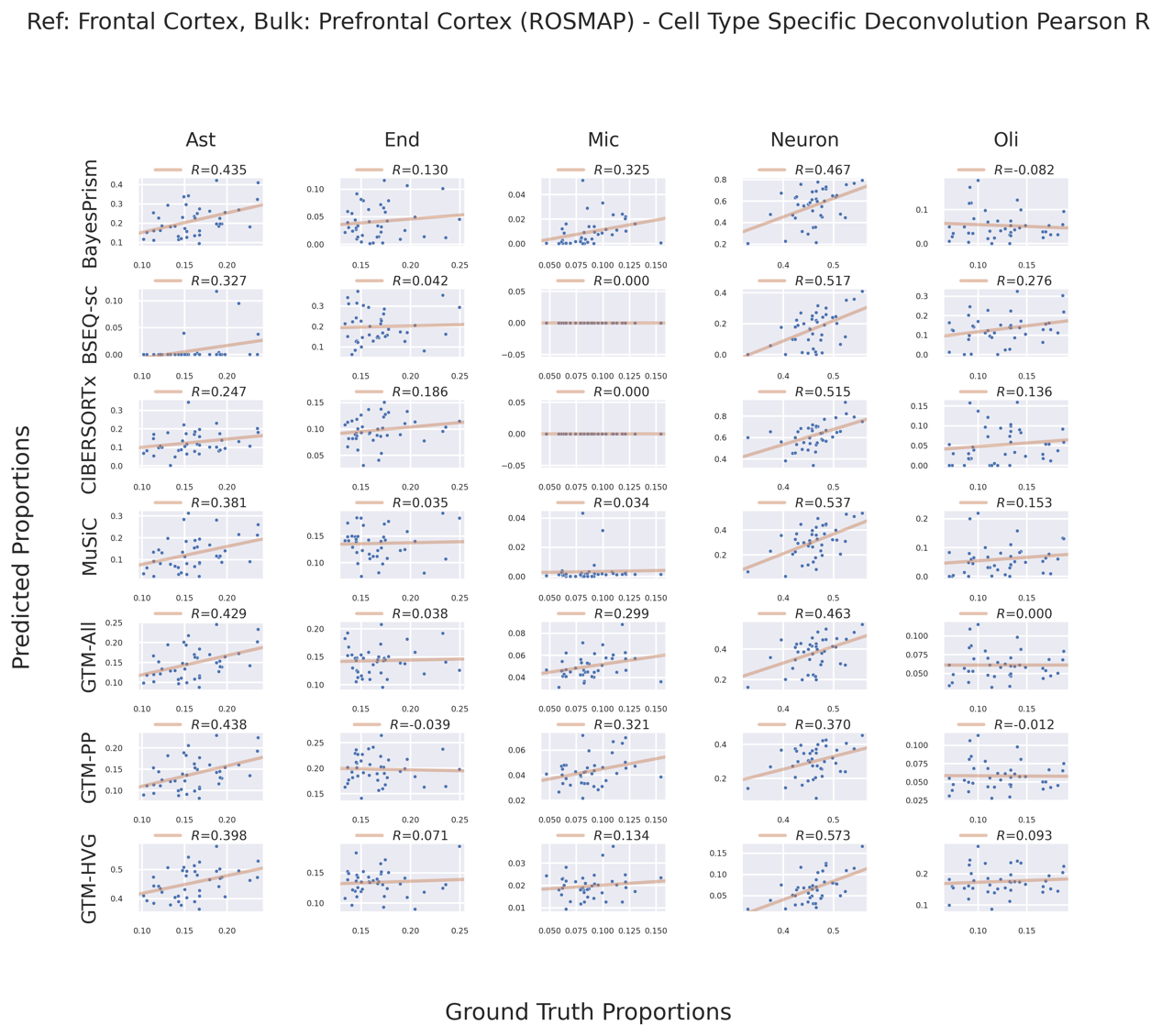


## Figure S14: Scatterplot of inferred cell-type proportion and ground-truth true proportion of real bulk data from Prefrontal cortex (ROSMAP in Table S2) based on a separate scRNA-seq reference of Frontal Cortex (Table S1).


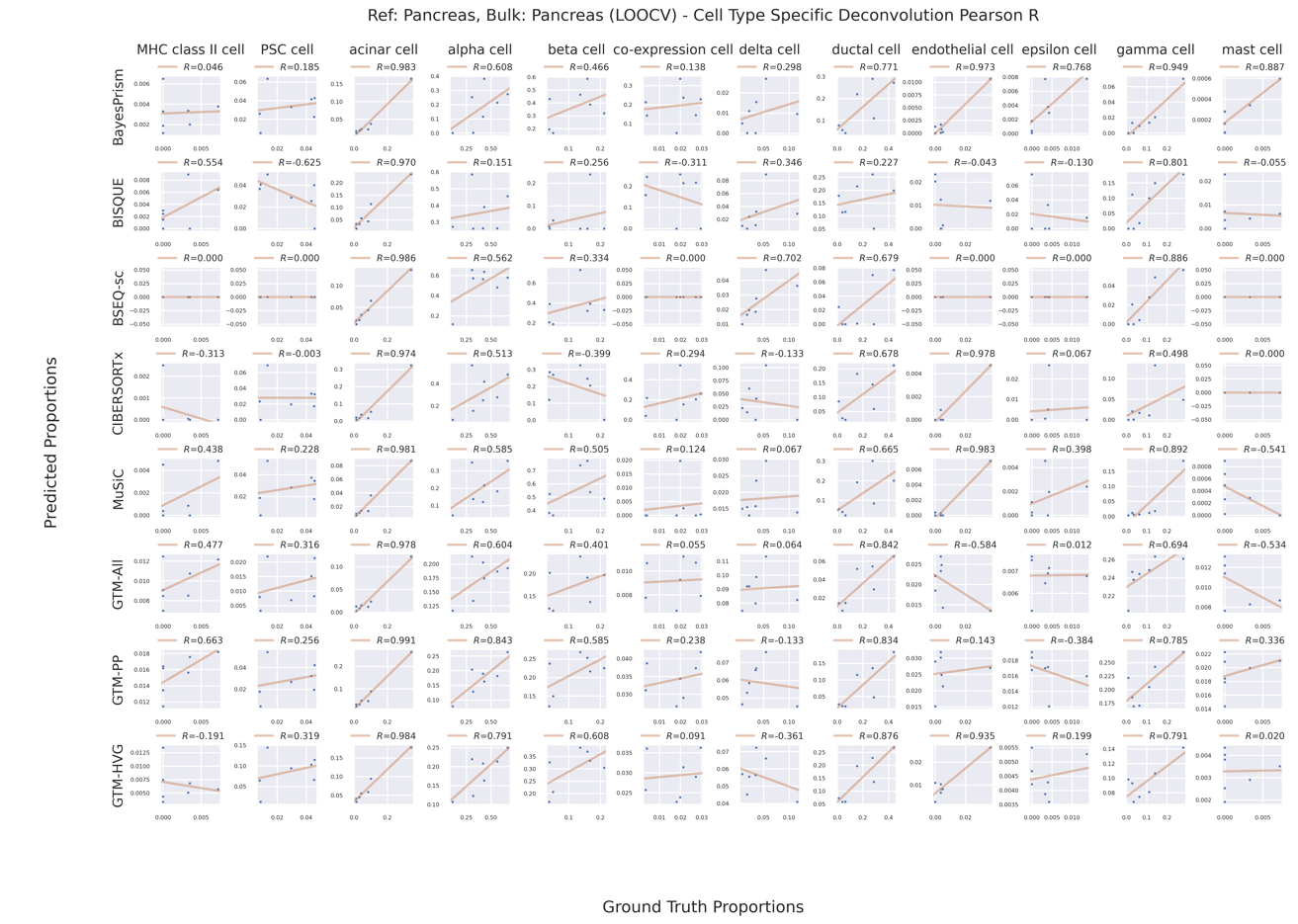


## Figure S15: Scatterplot of inferred cell-type proportion from real bulk data from Pancreas (Segerstolpe in Table S2) and estimated proportion from the paired scRNA-seq data in the same held-out subject. The scRNA-seq reference used to train each model came from the other subjects (Segerstolpe in Table S1).


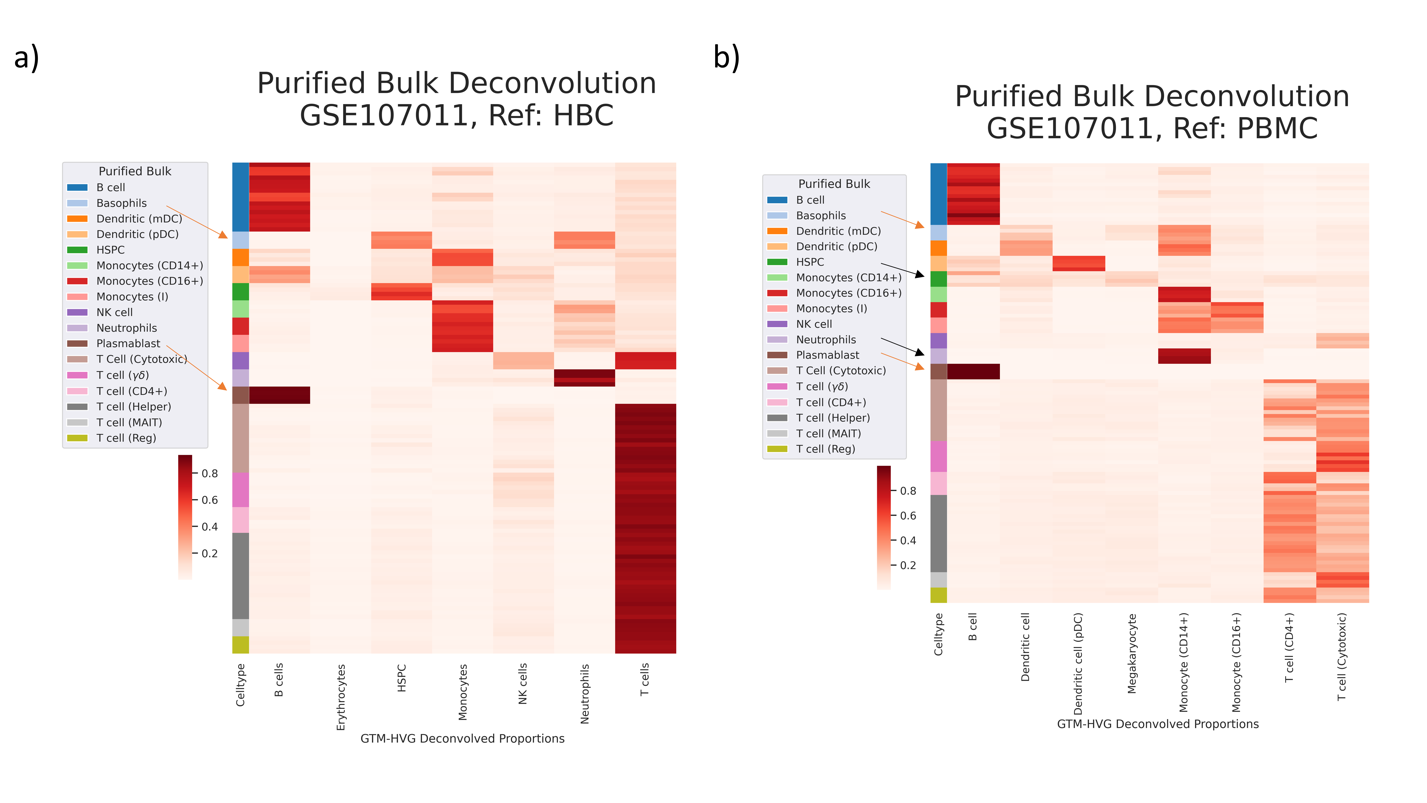


## Figure S16: Inferred cell-type proportion over purified bulk samples of immune cell types (GEO access: GSE107011). The CTS topics were inferred separately from two distinct scRNA-seq references with incomplete cell types with respect to the target bulk samples. We also experimented using either all genes or highly variable genes (HVG). The heatmaps show a qualitative comparison of the combinations of the reference and gene selection, where the rows are the bulk samples with color legend indicating their cell types and the columns are the cell types from the scRNA-seq HBC reference (left panel) and PBMC2 reference (right panel).


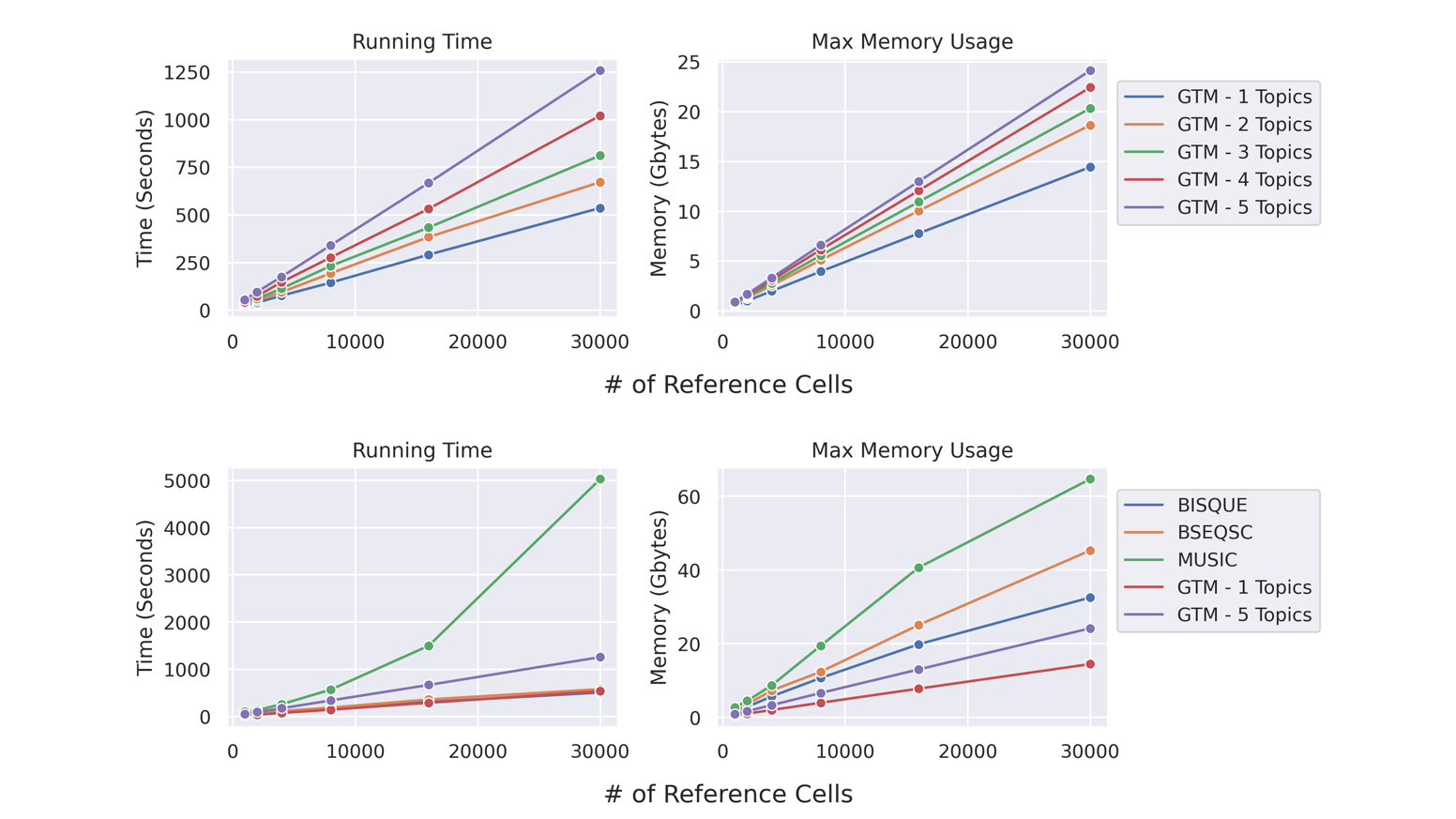


## Figure S17: Benchmark of time and memory usage of the deconvolution methods. We benchmarked the time and memory behavior or GTM-decon as a function of both input reference size and number of topics per cell-type. We trained on 1e3, 2e3, 4e3, 8e3, 1.6e4, 3e4 cells, with 33694 genes and deconvolved the same purified bulk PBMC data (GSE64655). BISQUE, BSEQ-sc, and MuSiC were all run with their default settings. All methods were run on a server with 62Gb RAM, 29Gb swap, with 20 Intel Xeon CPU e5-2650 v4s at 2.20Ghz. GTM-decon time and memory cost increase with the number of topics, but not exponentially, and GTM-decon at 1 topic per cell type has the lowest max memory usage and comparable run times to BSEQ-sc and BISQUE, and GTM-decon with 5 topics per cell-type still achieves much higher speed than MuSiC, the slowest model.


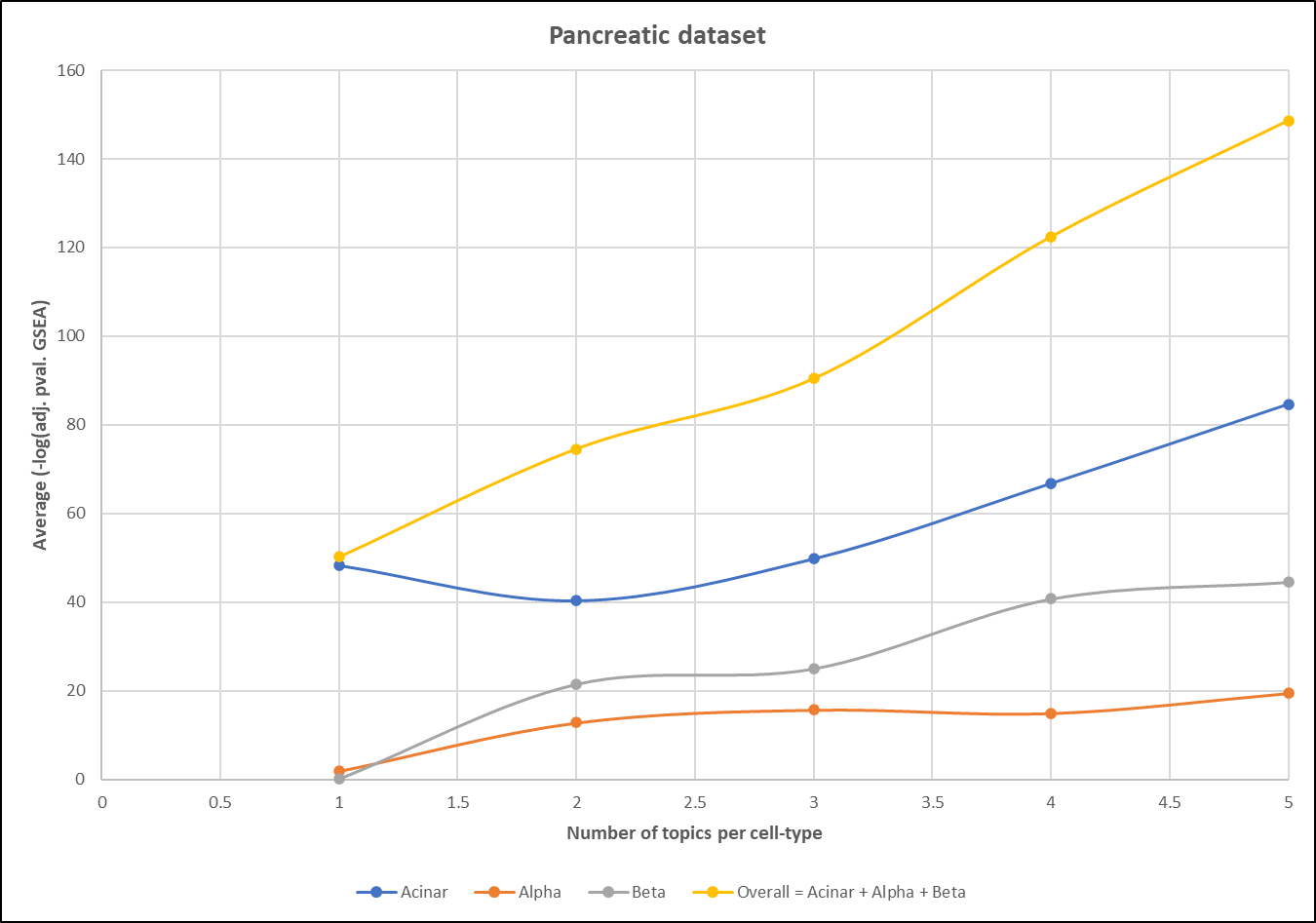


## Figure S18: Evaluating the number of topics per cell-type based on GSEA of known marker genes.  Cell-type-specific topics for Acinar, Alpha, and Beta cell types from Pancreatic Islet dataset from Segerstolpe et al. were evaluated based on whether the top genes are enriched for the known marker genes (from CellMarkerDB) under that cell type using gene set enrichment analysis. Each model was trained with varying numbers of topics per cell type (from 1 to 5). The plot shows the average (-log10 adjusted p-value) for the cell type of interest for the different topics. An overall value is calculated by summing up the values for the three cell types.


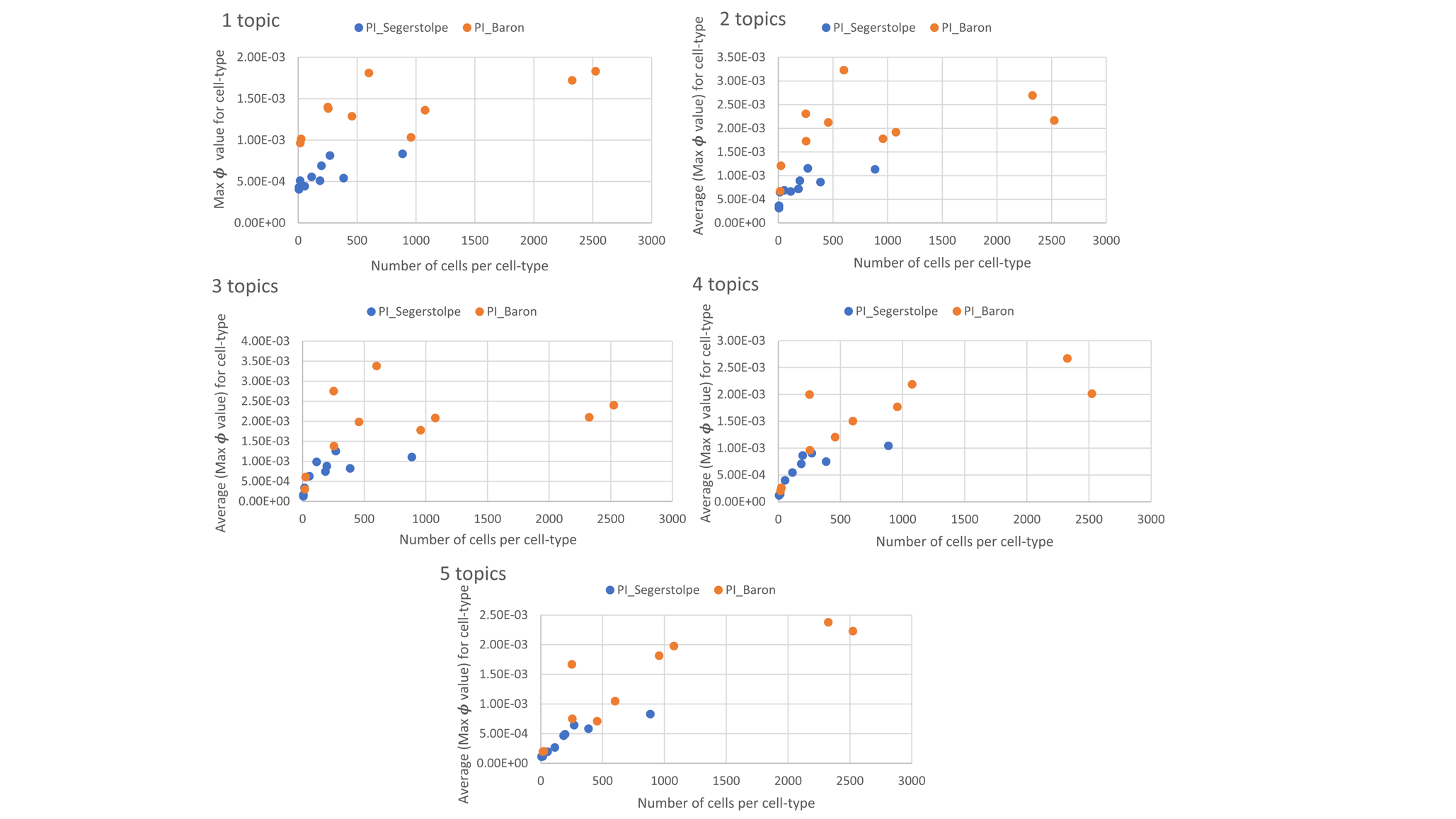


## Figure S19: Evaluating topic confidence in terms of number of cells per cell type. We evaluated topic confidence by correlating the topic confidence scores with the number of cells for each cell type available for training. The topic confidence scores were calculated by the first taking the maximum probability from the G-by-1 column vector $\boldsymbol{\Phi}_{\boldsymbol{k}}$ for each cell type $\boldsymbol{k\in1,\ldots,K}$ and then averaging over all K cell types. The resulting topic confidence scores were plotted as a function of number of cells per cell type. Each panel correspond to the number of topics per cell type (varying from 1 to 5) for two pancreatic datasets, Segerstolpe and Baron, generated using different approaches, Smart-seq2 and Drop-seq, respectively.


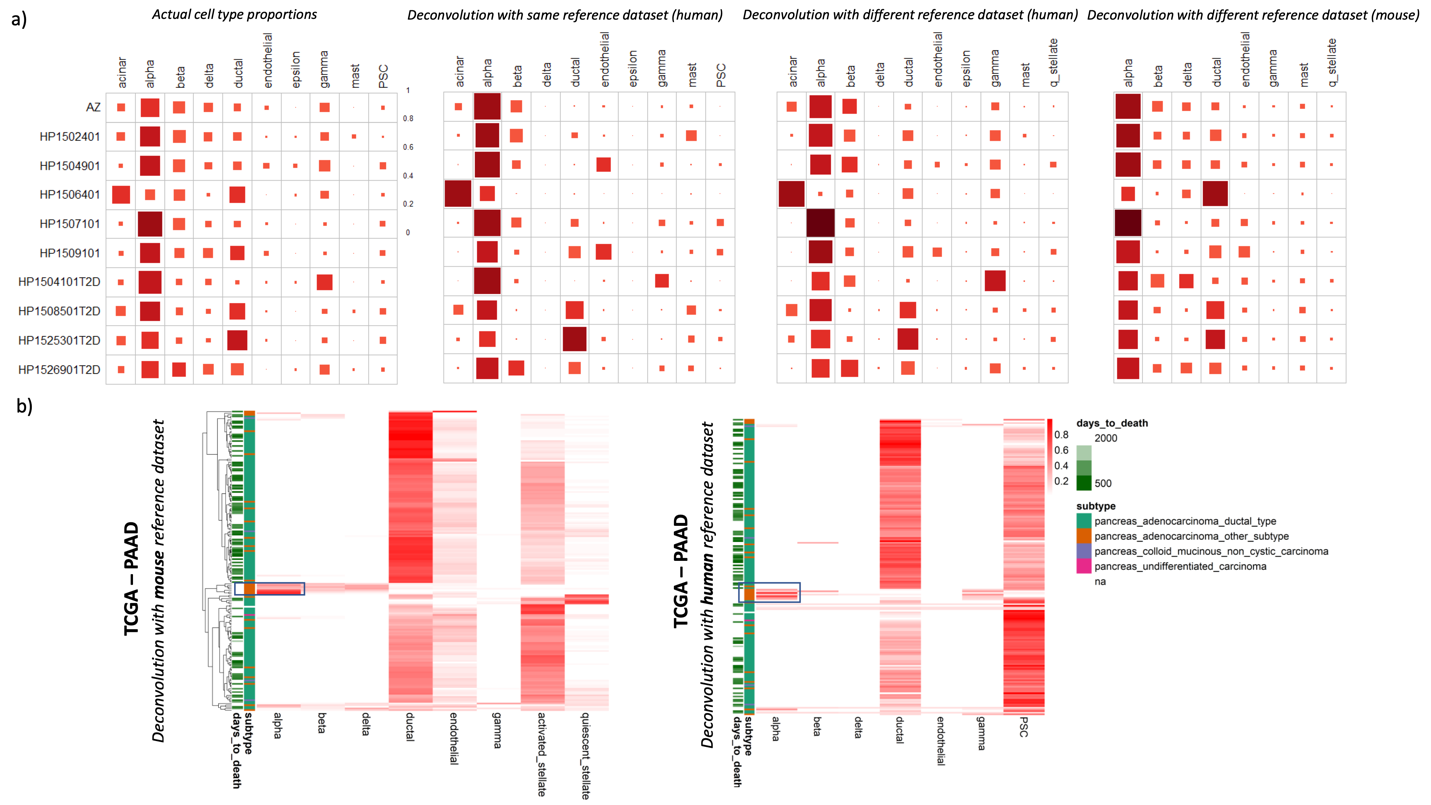


## Figure S20: Cross-reference and cross-species deconvolution. a) Deconvolution of PI-Segerstolpe human pancreas samples using different scRNA-seq reference data. The four Hinton plots from left to right display the ground truth cell-type proportions, the deconvolved proportions using the same reference dataset as target (i.e., Segerstolpe pancreas islet dataset), the deconvolved proportions using Baron pancreas islet scRNA-seq data as the reference, the deconvolved proportions using mouse pancreas scRNA-seq data as the reference. The rows are subjects and columns are cell types. b) Deconvolution of TCGA-PAAD dataset using human and mouse pancreatic datasets as reference. The samples (rows) were clustered by agglomerative clustering based on the GTM-decon model trained on the mouse reference (i.e., left heatmap). The same row order was applied to the heatmap based on human reference on the right. PSC in human dataset represents pancreatic stellate cells, which are represented as two different subtypes in mouse dataset. Four clinical phenotypes were shown as color bar on the left of each heatmap. Highlighted region for the alpha cell type was described in the main text.


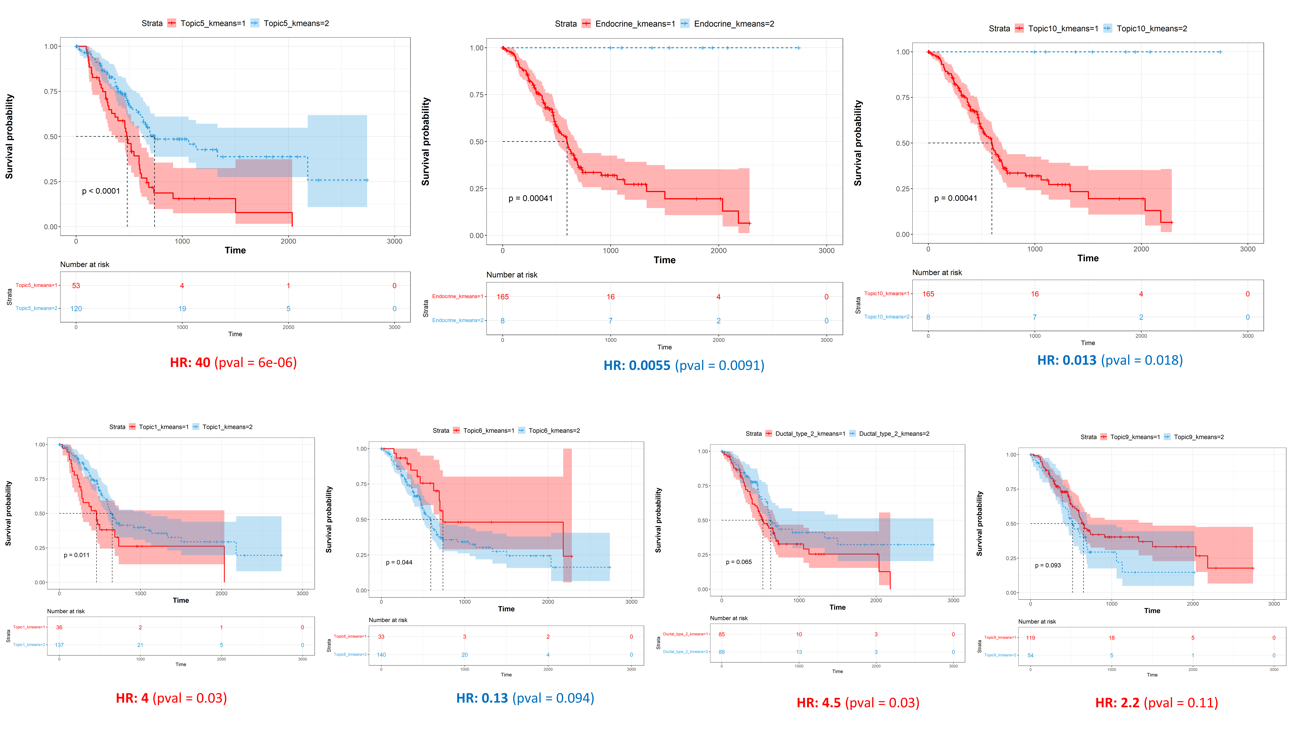


## Figure S21: Pancreatic cancer survival analysis of CTS and *de novo* bulkRS topics. To explore the marginal effect of individual cell type proportions on survival, we performed Kaplan Meier analysis by separating patients into two groups based on K means clustering. This analysis was performed on both CTS and *de novo* topics. Figure 4c lists the topics with the most significant differences (p-value < 0.1; log-rank test). The Kaplan-Meier curves for all those topics are shown here. The curve and shaded area represent the mean and standard deviation of the cell type proportions in the two groups, respectively. The number of subjects for each cluster were indicated below each Kaplan-Meier plot. The hazard ratios are shown at the end, with values < 0 indicating good outcomes, and values >0 suggesting poor outcomes.


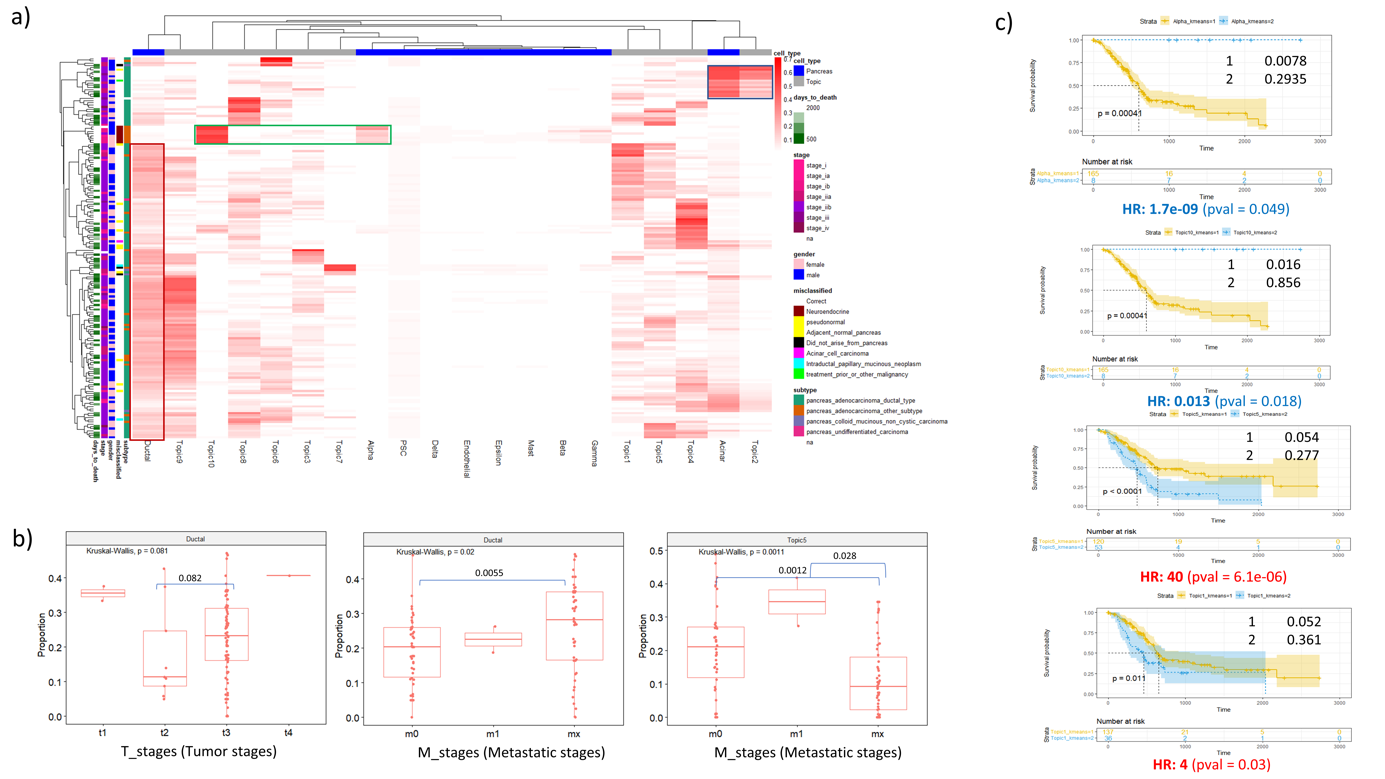


## Figure S22: Deconvolution of bulk RNA-seq samples for pancreatic cancer from TCGA-PAAD using healthy pancreatic scRNA-seq. a) Inferred cell-type proportions of TCGA-PAAD tumor samples. GTM-decon was trained an scRNA-seq dataset from individuals with pancreatic cancer. The trained GTM-decon model was then used to deconvolve the 174 TCGA-PAAD bulk RNA-seq profiles. In addition, we also ran unguided topic model (i.e., LDA) on the TCGA-PAAD bulk RNA-seq profiles directly to detect *de novo* topics that are not present in scRNA-seq reference. The above heatmap visualizes the combined deconvolution results based on the 10 pancreatic cell types, and 10 *de novo* topics (i.e., columns). Each of the 174 rows represents a subject. Five types of demographic or clinical phenotypes were shown in the legend to aid result interpretation. These include days to death, cancer stage, sex, misclassified status, and cancer subtype. The regions in highlighted boxes are described in the main text. c) Survival analysis using inferred cell-type proportions. The 174 subjects were divided into two groups based on K*-means cluster with K* set to 2 (not to be confused with the K cell types or topics). Kaplan-Meier curves for cell types and the *de novo* topics that resulted in significant differences in terms of their hazard ratios were displayed. The curve and shaded area represent the mean and standard deviation of the cell-type proportions in the two groups, respectively. The number of subjects for each cluster were indicated below the Kaplan-Meier plot.


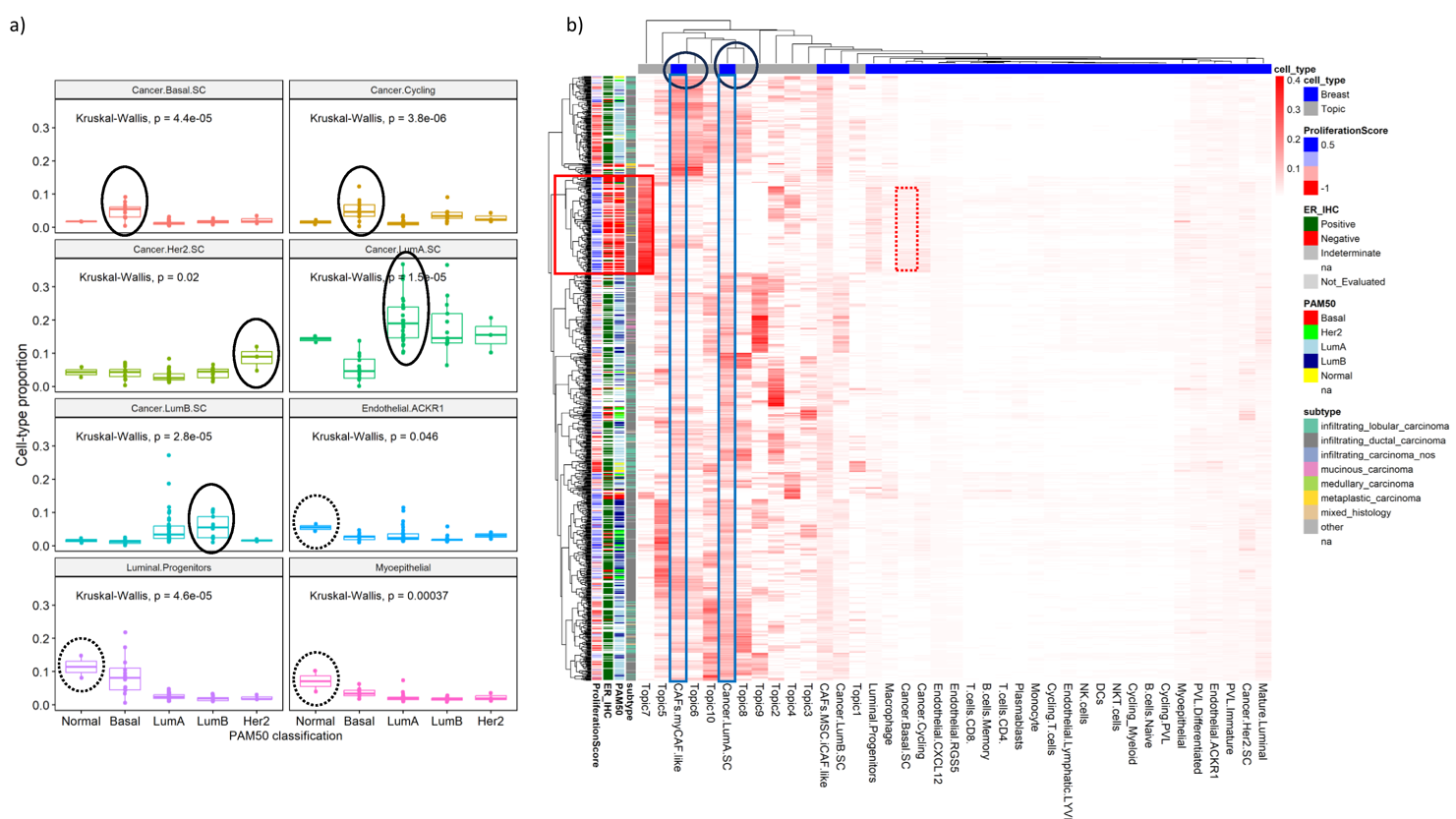


## Figure S23. Deconvolving breast cancer bulk transcriptome based on single-cell transcriptomes from breast tumors. a) Inferred cell-type proportions matched the cancer subtypes. We trained GTM-decon on the scRNA-seq breast cancer dataset using the 8 cell types as a guide. Each boxplot shows the distribution of the cell-type proportion for the samples separated by the cancer subtypes as defined by the PAM50 system. P-values based on the Kruskal-Wallis were shown. The subtype that exhibits the highest cell-type proportion in each panel was circled. b) Detailed visualization of the deconvolved proportions. The consolidated heatmap illustrates the cell type and *de novo* topic proportions from GTM-decon and unguided LDA separately trained on the scRNA-seq breast cancer data and TCGA-BRCA data, respectively. These two sets of topics were colored in grey and blue, respectively. The rows represent the 1212 tumor samples and the columns represent the cell types and *de novo* topics. The clinical phenotypes were annotated in the left color bar to help interpret the topics. Abbreviations of cell-types: CAFs – cancer associated fibroblasts; myCAF – myofibroblast; iCAF – inflammatory CAF; DCs – dendritic cells; NK – natural killer; NKT – natural killer T cells; PVL – perivascular-like.


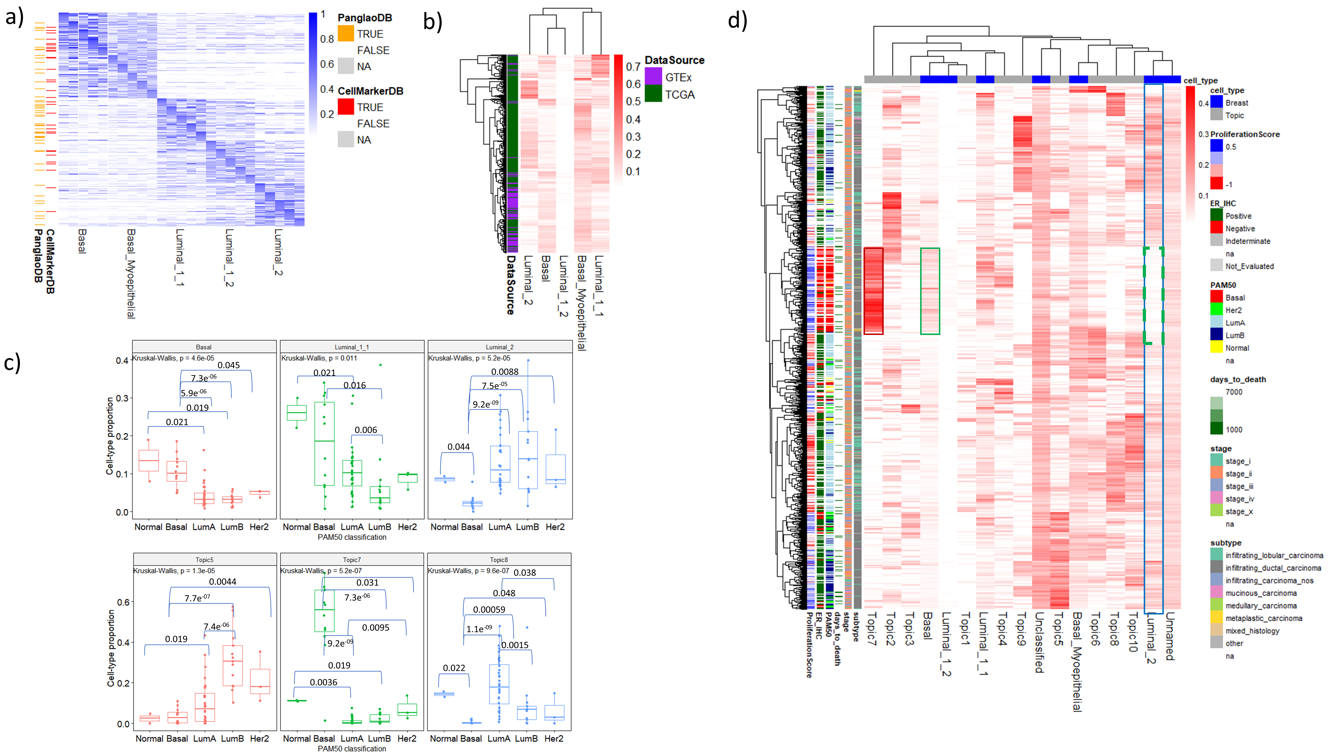


## Figure S24. Cell-type-specific inference and deconvolution of breast transcriptomes using HVG gene set from scRNA-seq data of healthy breast tissue. a) Gene signatures of cell-type-specific topics in breast tissue. We trained GTM-decon on a scRNA-seq reference of normal breast tissue [73] using 5 topics per cell type, on the set of highly variable genes. Same as Figure 3, the heatmap visualizes the top 20 genes per topic for the 5 main cell types in normal breast tissue (i.e., 25 topics in total for the 5 cell types). The heatmap intensity is proportional to the gene topic probabilities. Whenever available from CellMarkerDB and PanglaoDB, cell-type marker genes are indicated on the left. For the cell types, where marker genes are not available, “NA” were indicated on the left. b) Heatmap of deconvolved cell-type proportions for the main cell types for GTEx (normal samples) and TCGA-BRCA (cancer samples). The rows represent samples and columns represent the reference cell types. The heat intensity is proportional to the inferred cell-type proportions. Agglomerative clustering was applied to both the rows and columns to cluster samples and cell types respectively using Euclidean distance metric and complete linkage clustering. c) Comparison of inferred cell type or *de novo* topic proportions across breast cancer types. Each boxplot displays the distribution of cell-type proportions or topic proportions (y-axis) for the breast tumor samples separated by their cancer classes based on Prediction Analysis of Microarray 50 (PAM50) (x-axis). In each panel, p-value based on Kruskal-Wallis test was indicated on the top left corner and p-values based on Wilcox Rank Sum test for the significant pairwise comparison between PAM50 classes were also indicated. d) Comprehensive visualization of deconvolution results for the 1212 breast tumor samples from TCGA. The deconvolution results were consolidated from the inferred topics of three topic models, namely GTM-decon trained on breast scRNA-seq reference (i.e., the same model from panel a), GTM-decon trained on immune scRNA-seq reference, and unguided 10-topic LDA directly trained on the TCGA-BRCA. The three sets of topics were indicated by blue, green, and grey colors, respectively on the top color bar. The row represents the 1212 tumor samples with 6 types of clinical phenotypes annotation on the left color bar to aid result interpretation. The regions in the highlighted boxes were discussed in the main text. Deconvolution results using all genes were shown in Figure S25.


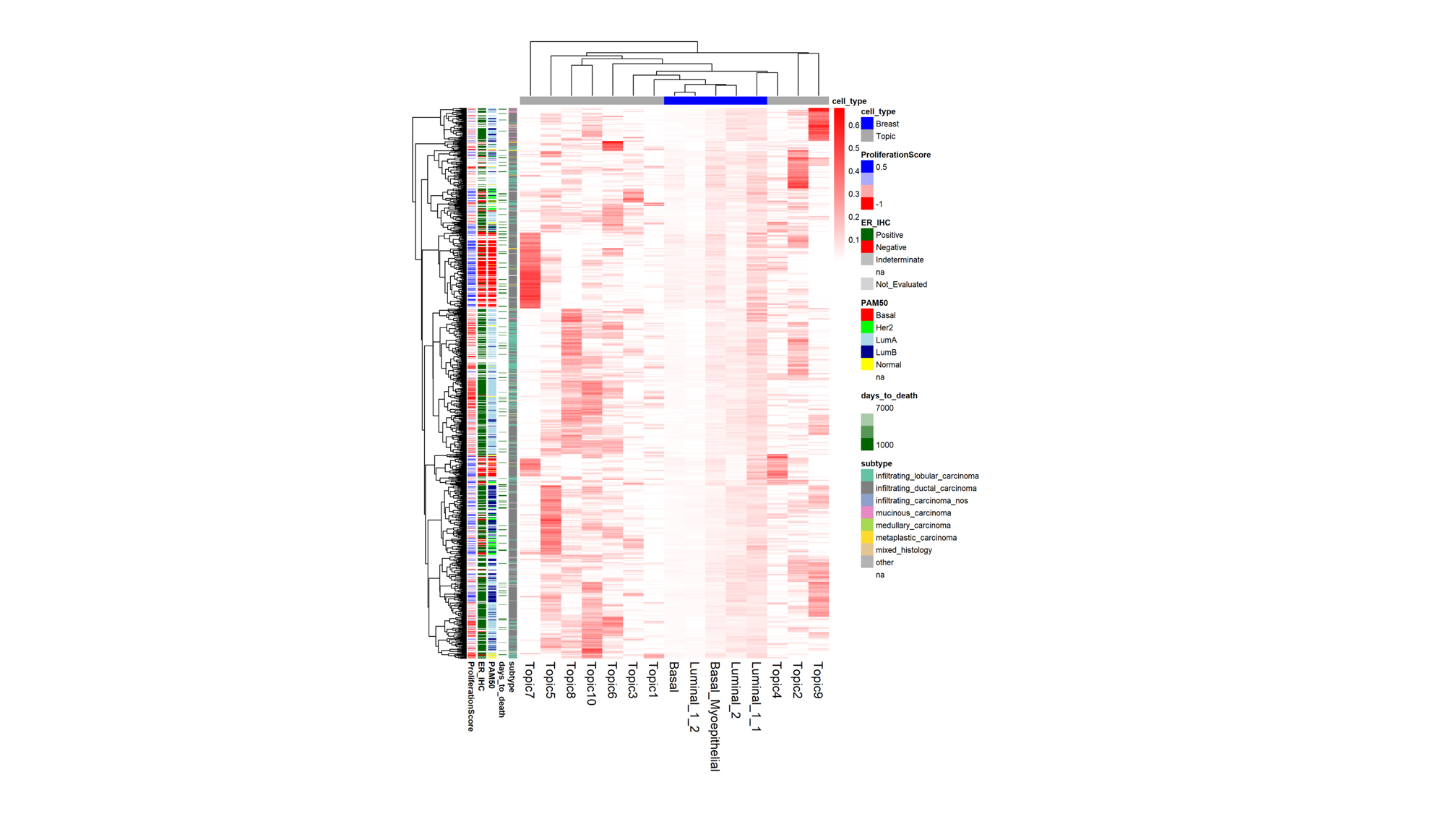


## Figure S25: Deconvolution of breast cancer transcriptomes from TCGA using *all genes*. Visualization of deconvolution results for the 1212 breast tumor samples from TCGA. The deconvolution results were consolidated from the inferred topics of three topic models, namely GTM-decon trained on breast scRNA-seq reference using all genes with 5 topics per cell type, GTM-decon trained on immune scRNA-seq reference using all genes with 5 topics per cell type, and unguided 10-topic LDA directly trained on the TCGA-BRCA. The three sets of topics were indicated by blue, green, and grey colors, respectively on the top color bar. The row represents the 1212 tumor samples with 6 types of clinical phenotypes annotation on the left color bar to aid result interpretation.


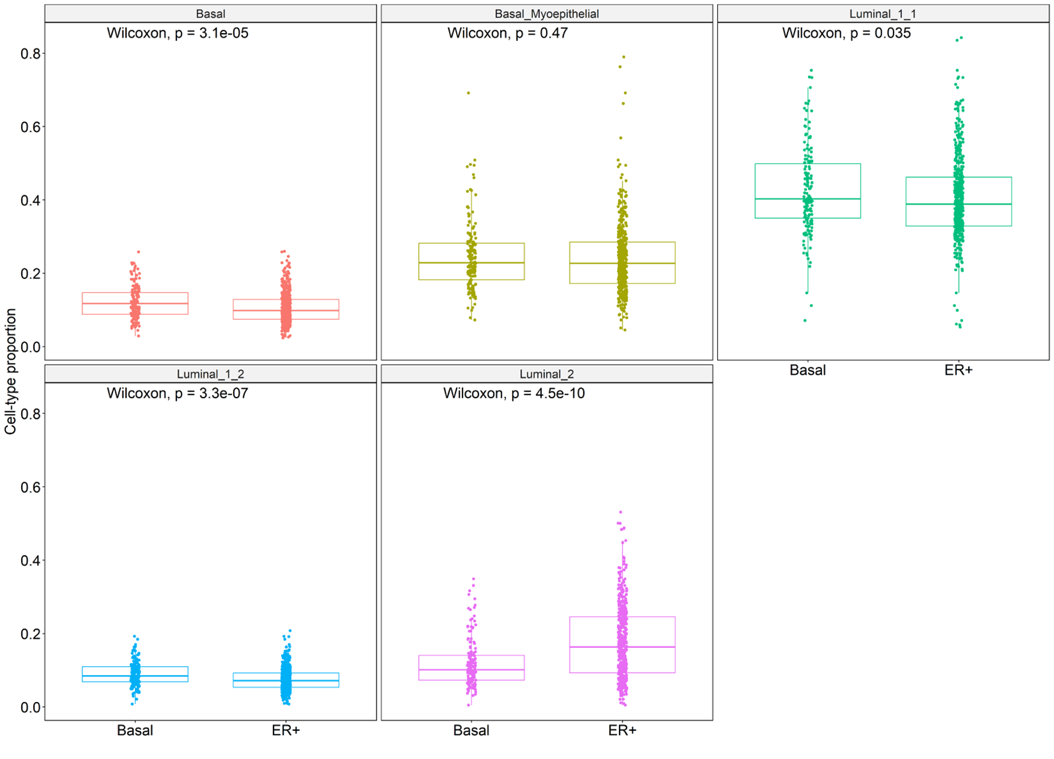


## Figure S26: Differential analysis of cell-type proportions by breast cancer subtypes of Basal and ER+. The box plots show the distribution of inferred cell type proportions for the five cell types in terms of the breast cancer subtypes – Basal and ER+. The box and the whiskers in each boxplot indicate the 25%-75% quartile and min-max of the evaluation scores for each of the samples. Statistical significance calculated using the Wilcoxon test suggests that four of the cell types show significant differences in proportions across the subtypes, with Luminal_2 exhibiting the highest difference.


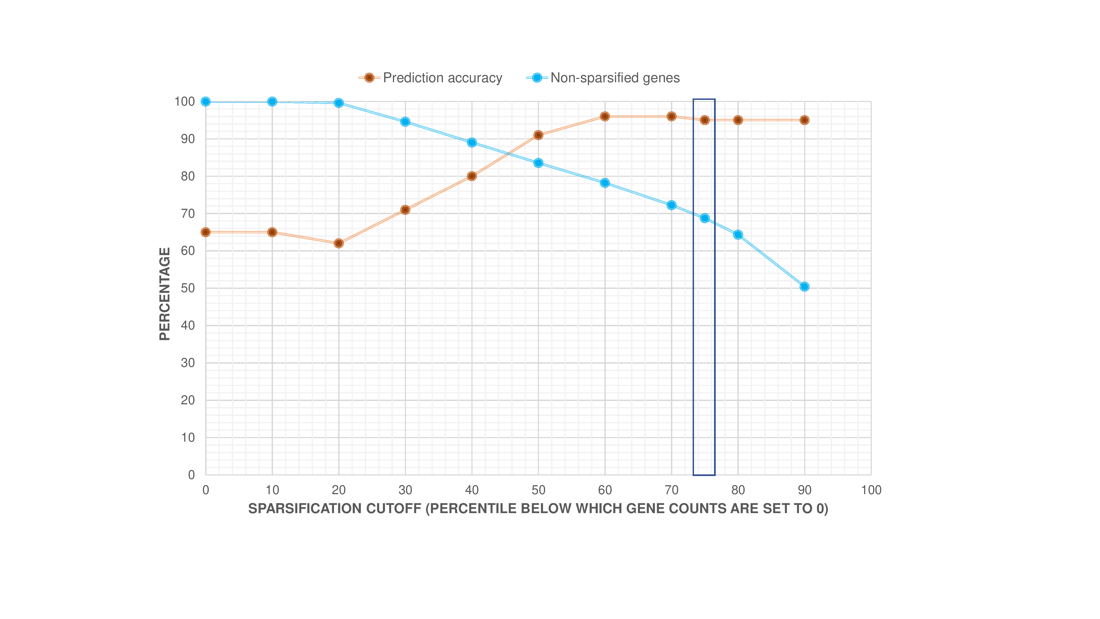


## Figure S27: Effect of sparsification rate on prediction accuracy of ER+ versus Basal subtype and percentage of non-zero genes. In order to effectively capture phenotype signatures directly from the bulk RNA-seq data using our topic modeling approach, the TCGA-BRCA RNA-seq data were sparsified to varying degrees by setting the values below the “n-th” percentile to be zero. We varied the percentiles from 10 to 90, with the 75-th percentile representing the value chosen based on scRNA-seq datasets. The model performance was evaluated as the phenotype prediction accuracy comparing the phenotype-specific topic probabilities with the groundtruth ER+ and Basal on the held-out 20% subjects. The percentage of the non-zero genes (i.e. genes which have non-zero counts in at least one sample after the sparsification procedure) was also shown in the blue curve as a function of sparsification rate.


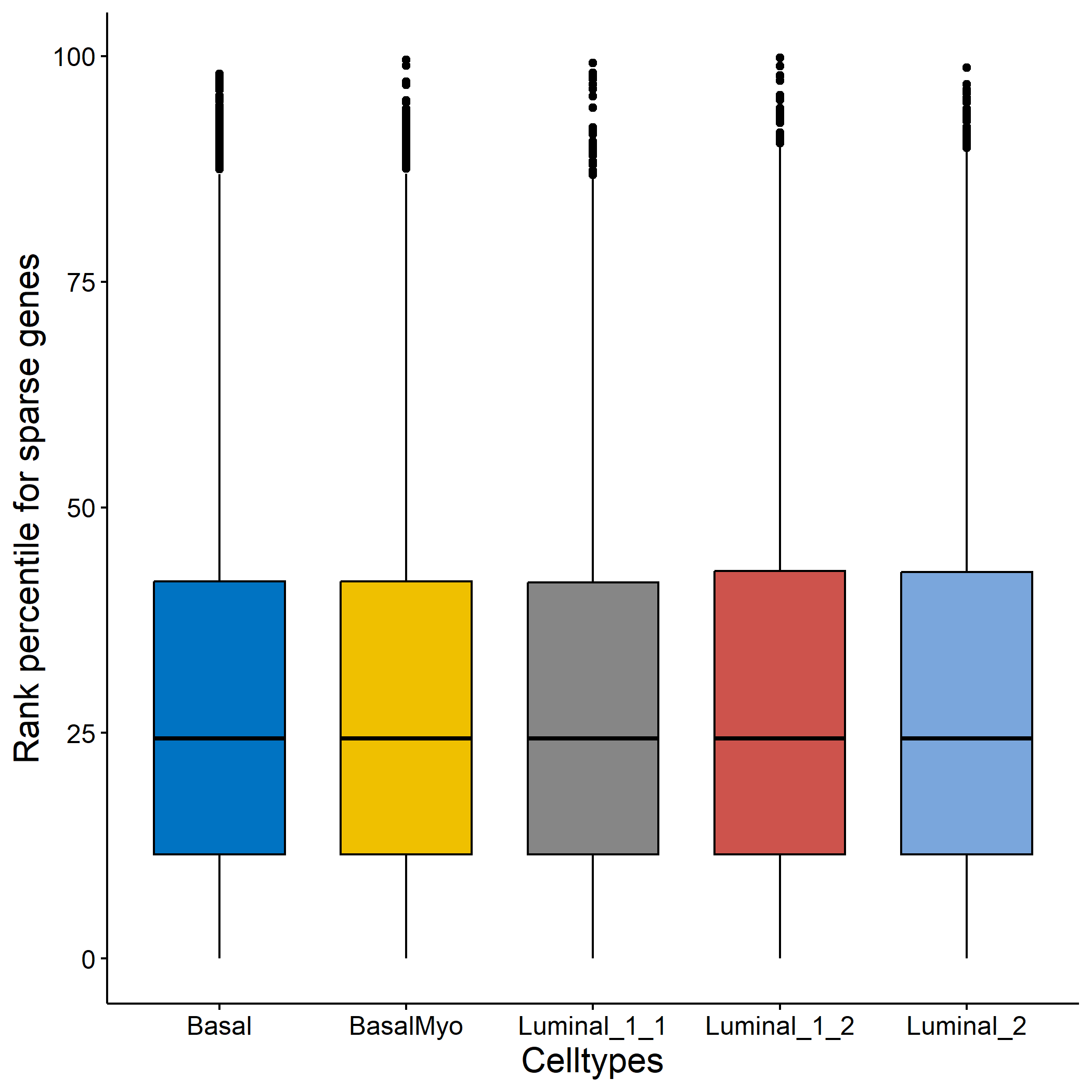

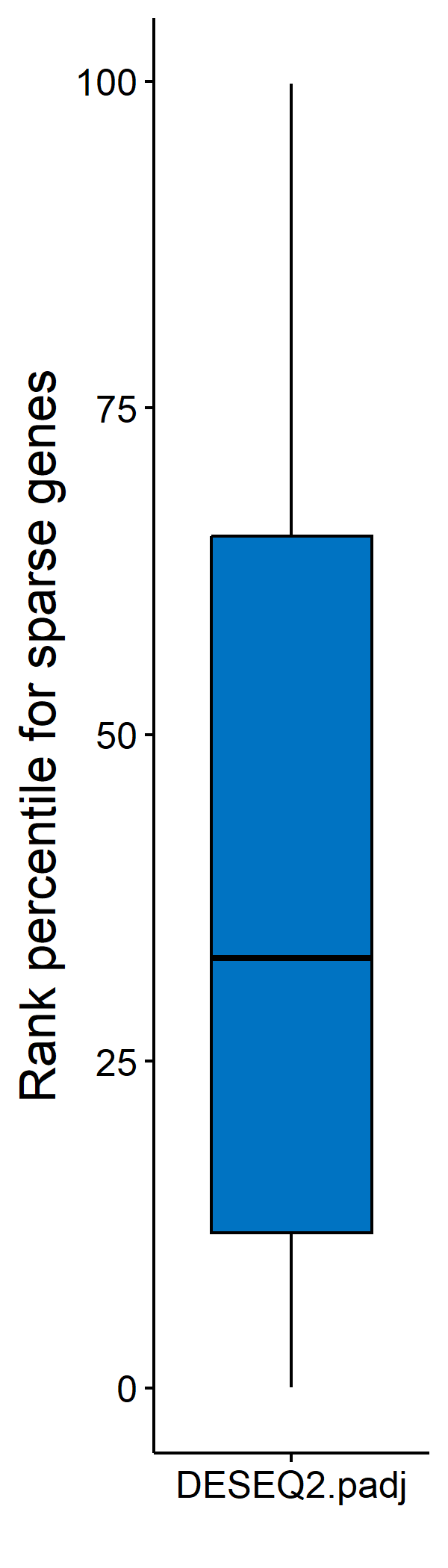


## Figure S28. Marginal contribution of zero genes to the CTS topics and DE analysis. Left panel: the boxplots show the distribution of rank percentiles of the genes-by-CTS topic probabilities of the zero genes due to 75-percentile sparsification in the entire gene list for the 5 cell types from the scRNA-seq data of breast tissues. The gene with the highest probability for that cell type is set at 100-th percentile rank and the gene with the lowest probability at the 0-th percentile. Right panel: The boxplot shows the distribution of the rank percentiles of adjusted p-values of DE genes identified by DESeq2 from whole bulk. The gene with the lowest p-value was set at 100-th percentile rank and the gene with the highest p-value at the 0-th percentile.


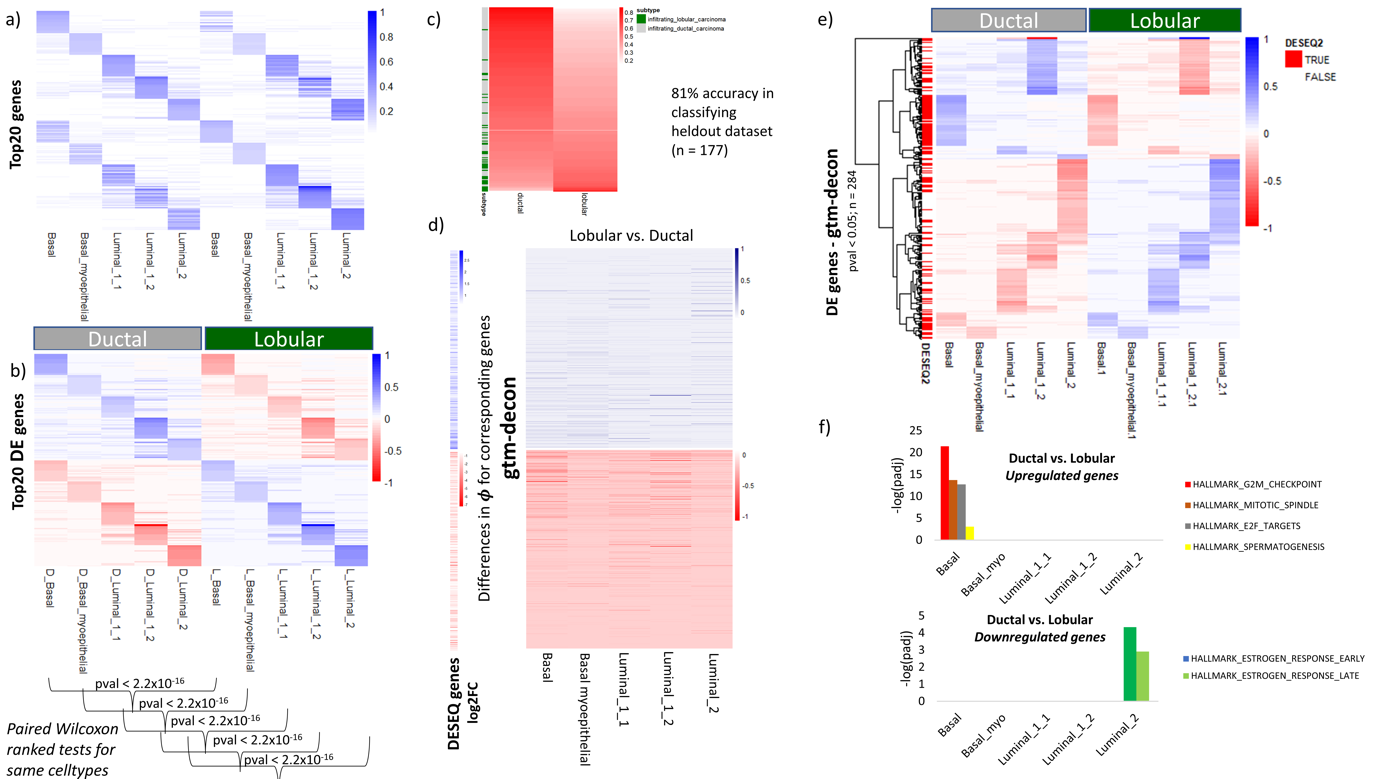


## Figure S29: Identification of cell-type-specific differentially expressed genes from bulk RNA-seq data comparing between ductal and lobular breast carcinoma subtypes. a) Bulk RNA-seq samples of ductal and lobular subtypes of breast carcinoma from TCGA were analyzed using the same approach described in Fig. 6 to identify cell-type-specific differentially expressed (DE) genes corresponding to these two subtypes. a) Top cell type specific genes in each subtype. Visualization of top cell-type-specific gene signatures for Ductal and Lobular carcinoma in terms of gene-by-cell-type proportions for each subtype. b) Top DE genes in each subtype. Visualization of top predicted differentially expressed (DE) genes for each cell type between Ductal and Lobular carcinoma in terms of gene-by-cell-type proportions for each subtype. Paired Wilcoxon-ranked tests for same cell types in the two subtypes reveals significant differences in gene-cell-type probabilities for all cell types (p-value < 2.2x10^-16^ for all comparisons). c) Classification of subtypes based on phenotype probabilities. Visualization of phenotype probabilities inferred for ductal and lobular subtypes for the held-out set. The plot is ordered based on decreasing values of inferred phenotype probabilities for ductal subtype (rows), with the phenotype labels indicated on the left color bar. d) Comparison with DE genes from DESeq2. Comparison of gene-topic scores inferred by our approach for DE genes detected by DESeq2 in a Lobular versus Ductal comparison. On the left, the log_2_ fold change in expression of upregulated genes (top half) and downregulated genes (bottom half) identified by DESeq2 are arranged in decreasing order of the adjusted p-values. On the right, a heatmap of differences in gene-by-topic proportions for the same cell type, inferred by our method for a lobular versus ductal comparison, is shown for corresponding genes. e) DE genes identified by our method. Heatmap of 284 statistically significant DE genes (p-value < 0.05) identified by our method (rows), depicting difference in gene-topic-scores for the same cell types. The top cluster shows the upregulated genes in ductal subtype while the bottom cluster shows the downregulated genes. DE genes corresponding to those identified by DESeq2 are indicated in the left color bar. f) ORA analysis of differences in gene-topic proportions. Statistically-significantly enriched HALLMARK pathways identified in an ORA analysis based on differences in gene-topic-scores per cell type for ductal versus lobular comparison are indicated, for both upregulated and downregulated genes. Their significance level is indicated by the -log (adjusted p-value) on the y-axis, for the different cell types on the x-axis.


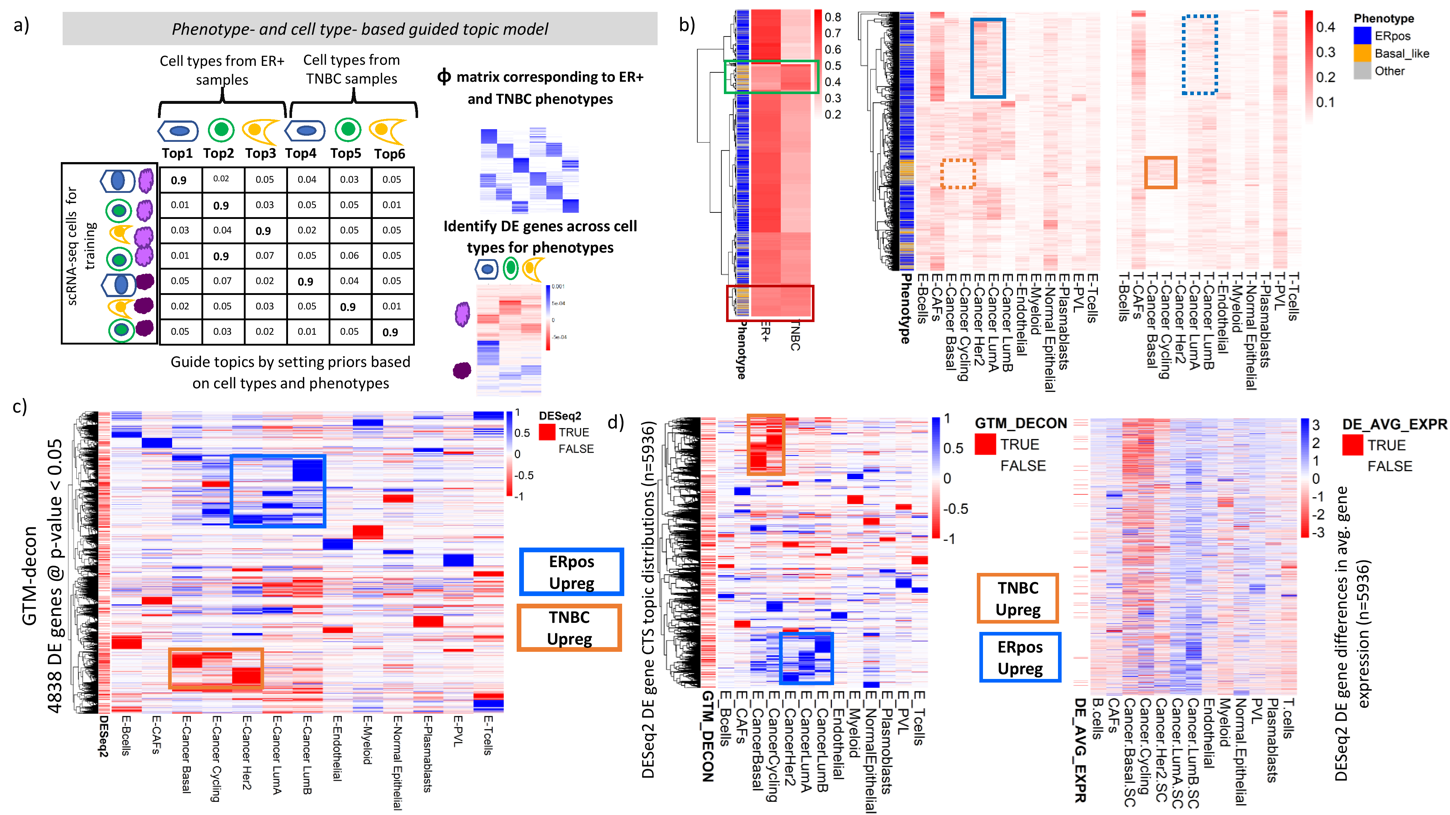


## Figure S30: Phenotype-CTS guided topic inference of ER+ and TNBC breast cancer scRNA-seq data.

**a**) Briefly, we inferred the phenotype-CTS topics by assigning one topic to each combination of cell type and phenotype. During GTM-decon training, the topic designated for the observed cell type and phenotype is endowed with a topic prior value of 0.9, and the rest of the topics were set at a random value between 0.01 and 0.1. As a result, these priors guide the topic model to infer phenotype-CTS topic distributions. All the genes in the experiment were used as features.

**b**) Phenotype and phenotype-CTS deconvolution of TCGA-BRCA samples (n=1212). In both heatmaps, each row represents a bulk sample. For  the left heatmap, the columns indicate the phenotype-specific topics, and for the right heatmap the columns indicate phenotype-CTS topics, where the prefixes ‘E’ and ‘T’ before the cell type names indicate ER+ and TNBC cancer subtype, respectively. Enrichment (solid rectangle) and depletion (dash rectangle) for known cell types for the cancer subtypes were indicated using blue rectangles (for ER+) and orange rectangles (for TNBC), respectively.

**c**) Genes-by-CTS topic proportions for the 4838 statistically significant DE genes at the empirical p-value < 0.05 based on 100,000 permutation tests, out of which 1687 genes overlap with the 5936 DE genes identified from bulk TCGA-BRCA via DESeq2. The rows correspond to DE genes, and the columns to the change of CTS topics for ER+ with respect to TNBC (therefore indicated as columns with prefix ‘E’). The differences for TNBC minus ER+ comparison are the mirror image of this and hence not shown.

**d**) Differential signals of the TCGA-BRCA DE genes from the DESeq2 analysis of the TCGA-BRCA data. We visualized the differential signals of the DE genes in two ways:

(i) Differential CTS topic probabilities. The left heatmap shows the change of the genes-by-CTS topic probabilities in ER+ w.r.t. Basal-like subtype from the single-cell breast cancer transcriptomes for the 5936 DE genes detected by DESeq2 for ER+ cancer subtype w.r.t. Basal-like cancer subtype from the bulk TCGA-BRCA transcriptomes. Among these 5936 DE genes, 1687 genes were among the 4838 DE genes detected by 100,000 permutation tests from the scRNA-seq breast cancer data based on the differences in CTS-topic probabilities between the ER+ and TNBC phenotypes for each cell type. These genes are shown by the row annotations on the left side of the heatmap.

(ii) Differential average gene expression for ER+ subtype w.r.t. TNBC subtype per cell type. The right heatmap shows the average gene expression differences between the ER+ subtype and TNBC subtype calculated directly from the observed single-cell data. For the ease of comparison, the rows were ordered according to the clustering pattern from the left heatmap. Among the 5936 DE genes from DESeq2, 592 genes were among the 1892 DE genes detected by 100,000 permutation tests based on the differences in average gene expression between the ER+ and TNBC phenotypes for each cell type. These genes are shown by the row annotations on the left side of the heatmap.


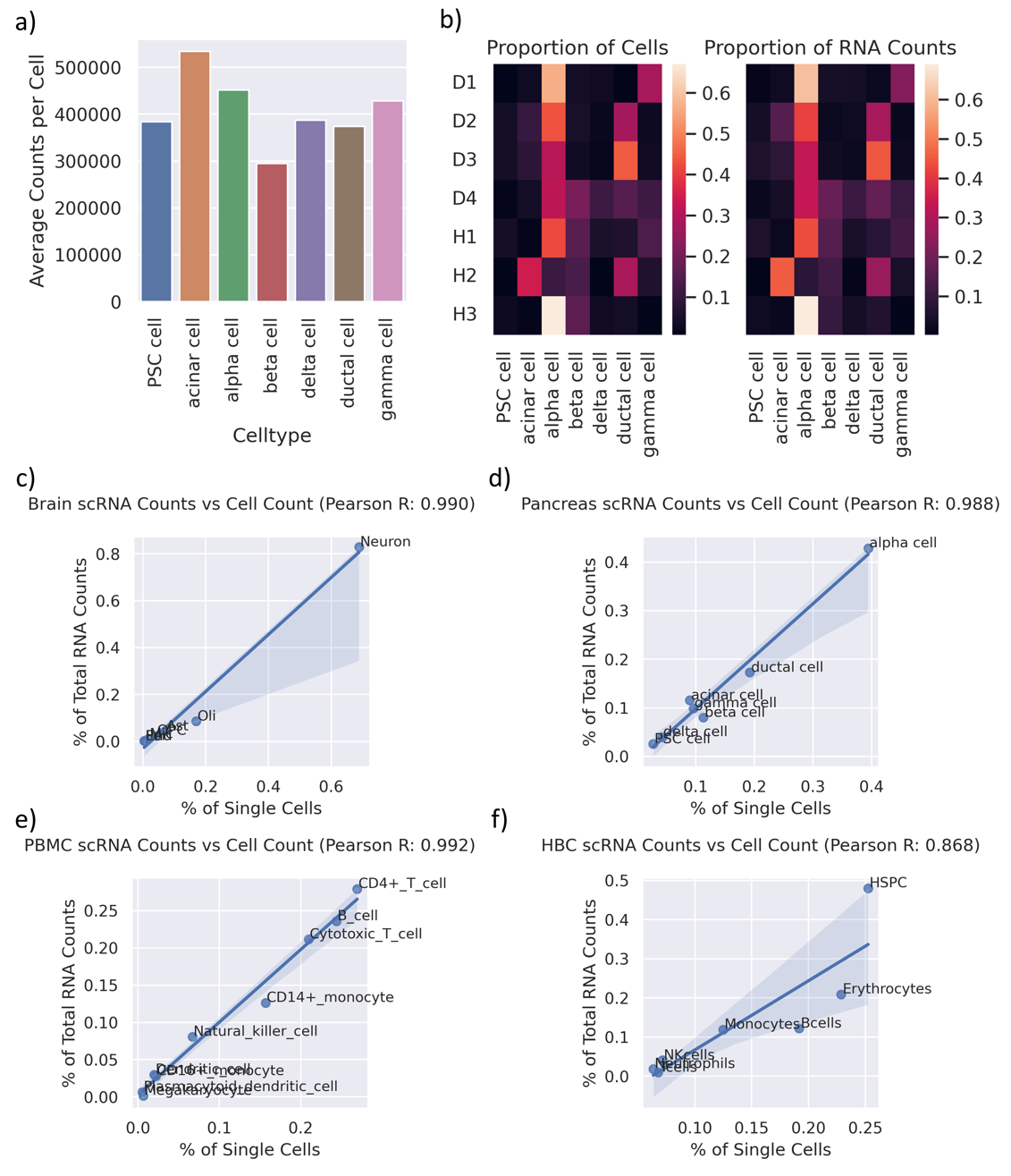


## Figure S31. Correlation between Cell Fraction and Cell-type Specific RNA Fraction. a) Average number of RNA read counts per cell-type in single-cell Pancreas Data (cell-types with >40 cells available). b) Heatmaps comparing cell fraction and RNA fraction per cell-type for Pancreas single-cell Data across 7 patients. c-f) Scatterplots comparing the total percentage of RNA read counts for a given cell-type vs the fraction of single cells of that given cell type for the Prefrontal Cortex, Pancreas, PBMC, and HBC single-cell references respectively.
