# Supplementary material for: GTM-decon: guided-topic modeling of single-cell transcriptomes enables sub-cell-type and disease-subtype deconvolution of bulk transcriptomes
Source: Genome Biol. 2023 Aug 18;24:190. doi: 10.1186/s13059-023-03034-4 (PMC10436670; doi:10.1186/s13059-023-03034-4)
Supplement: Supplementary file 2 — Additional file 2. Review history. [file 13059_2023_3034_MOESM2_ESM.docx]

**1^st^ round**

**Reviewer 1**

This study highlights the importance of heterogeneity in studying the progression of several diseases including cancer. It also highlights the high cost, lower throughput and difficulties in sample preparation challenges involved using experimental approaches at the patient population scale motivating the need for computational deconvolution methods such as GTM-decon. The authors claim GTM-decon is a highly efficient and interpretable Bayesian approach for cell-type deconvolution, based on guided topic modeling which learns celltype-specific gene signatures in the form of categorical distributions (i.e., latent topics) from scRNA-seq datasets.

Many computational methods have been developed to infer cell type proportions from bulk transcriptomics data including benchmarking of these methods guided by scRNA-seq signatures. While the authors try to make the case that GTM-decon is highly interpretable compared to traditional methods and the fact that single-cell research is increasingly getting cheaper and mainstream, I find the proposed methodology, results and the significance of the entire study limited in justifying this claim among others including innovation, clarity, reproducibility and generalizability in advancing the field.

Major issues:

Interpretability as a selling point of the paper needs to be properly defined with clear literature and metric supporting how this feature has been investigated in the field. Furthermore, given that that the performance of GTM-decon depends on sensitive labeled scRNA-seq data, whose processing workflow involves challenges such as cell sorting, normalization, gene dropouts, feature selection and transformation, matching cell types during disease progression etc. The weak sensitivity or benchmarking analysis and lack of clarity highlighted below in generating the tumor-immune reference signature data, the poor labeling of the tumor immune cell types and the proposed simulation results based only on high variable genes demonstrates bias in the performance results and interpretability by the method.

For example, labeling fibroblasts as immune cells show fundamental lack of the understanding of tumor-immune biology. Furthermore, using independent breast and RA tissues from different patients to validate the deconvoluted tumor immune cell types without a clear analysis of between and within variability is bias. There is no clear justification why immune cells from the same tumor microenvironment or tumor region is not used as reference given joint single-cell transcriptional/protein and imaging data can be obtained. It is also not clear how the proposed method performs with missing important cell types in the reference data or how the differential gene analysis takes into account the changes in proportions of cell types between normal and disease states. Furthermore, the proposed approach of simulating bulk from single-cell data lacks statistical or biological validation and the effect of using different forms of normalization and batch corrections for various datasets need to be properly investigated.

Other issues:

In page 6 line 14-16, the authors use all gene expression profiles as input to build a reference model. By all genes do they mean all the genes in the original raw data? If not how were these genes selected?

On figure 2a, the authors claim GTM-decon performs better than other models on the Pancreas and Breast Tissue datasets in terms of both Spearman R and Cross Entropy and conferred comparable performance on the RA Synovum dataset which also seem to produce smaller variance for both the pancreatic and breast tissue datasets. The better performance observed is mostly breast and the spread of points are overlapping in these applications. Comparison of multiple datasets is needed to support such superior performance of GTM-decon including the validation of ground truth proportions.

GTM-decon uses a strong prior (0.9) to anchor each topic to a specific cell type and subsequently guide the inference of the global topic distributions over genes to automatically prioritize cell-type marker genes. What happens if the prior is varied from (0.6-0.8) which is still better than chance.

More detail about the harmonization of both pancreatic tissue and immune tissue to the same numerical scale needs to be provided with more sensitivity analysis to support the results.

In figure 4a the authors suggests that the bulkRS topics and the reference capture complementary information. What does this mean biologically?

They claim "GTM-decon is not sensitive to different reference scRNA-seq datasets from the same tissue even if they were generated from different platforms (e.g., Smart-seq and Drop-seq)". Making such a statement from a small replicate analysis is bias given that several studies have shown that different protocols are optimized for different scientific objectives and differ in terms of number of sequenced genes produced.

It is hard to follow the model description and annotations in the caption of Figure 1 with the figures.

Furthermore, the instructions in the GitHub link provided with python code is difficult to follow. Generating a single script to test simple examples will help. It is not possible to know if the results presented can be reproduced or not.

Minor concerns

1) The authors use a the word "guided topic modeling ", "topic" or "phenotype-specific topic" throughout the text without a clear definition especially given these are not standard methodology in the field.

2) Full definitions of MixEHR-Guided model including MixEHR and sureLDA models should be defined

3) It seems figure 1b-c not referenced in the text

4) There are some typo omissions here and there; Line 5-9 page 6 "cell type tends provided better performance", …. We also assessed the effects of using different genes selection strategies, Line 34-36 page 10 …Notably, the pancreas adenocarcinoma other subtype is predicted to originate from alpha cells, as observed in humans, Line 19-21 Page 13 needs review.

**Reviewer 2**

The authors present a new Bayesian method "GTM-decon" based on guided topic modeling, which aims at automatically learning cell-type-specific gene signatures from scRNA-seq data to deconvolve bulk RNA-seq data. They validate their method through simulated and real data experiments. Interestingly, they also extend their method to learn phenotype-guided topics and identify cell-type-specific differentially expressed genes. I have a few questions about this work.

1. The authors use a guided topic modeling mechanism to learn cell-type-specific gene signatures. What are the advantages of using this mechanism over calculating average gene expression from scRNA-seq data directly?

2. The authors assess the effects of using different data transformations on the scRNA-seq data. However, a more common transformation is ignored by the authors, which is to normalize counts first and then perform logarithmic transformation. In addition, after preprocessing the data, the data will no longer be integers, what way do the authors use to make it fit the topic model?

3. The prior distribution of Φ_k is a Dirichlet distribution with a fixed hyperparameter β. How do the authors set this hyperparameter? How would different settings of it affect the results?

4. Could the authors provide a more detailed description on how to use genes-by-topics estimates to deconvolve bulk RNA-seq data? For example, does bulk RNA-seq data need to be preprocessed? What is the data generative process for the gene expression of each sample in bulk RNA-seq data? What are the specific calculations and formulas?

5. When using standard LDA for deconvolution, how do authors determine whether a detected topic corresponds to a known cell type or a new cell type? Are there any metrics? Since there is no prior information, the detected topics may be a combination of multiple cell types, or may be independent of cell types. How to better explain these topics?

6. When deconvolving bulk RNA-seq datasets using multiple scRNA-seq datasets, the authors first estimate the genes-by-topics matrix independently for each dataset and then combine them. Compared with combining the multiple datasets first and then estimating the genes-by-topics matrix, what are the advantages of this way?

7. As can be seen from Figure 3a, no statistically significant genes are found for three cell types, including endothelial, epsilon, mast. How to explain this phenomenon? Will this affect the subsequent deconvolution of the bulk RNA-seq data?

8. The authors deconvolve cancer samples using scRNA-seq reference from noncancerous tissue. The cell types they contain may be different. Does this have an effect on deconvolution? Will the results of deconvolution be biased?

9. The authors need to carefully check the writing of mathematical formulas and give definitions of all symbols involved. For example, the authors define n_(m,.,k)^(-(i,m) ) and n_(.,g,k)^(-(i,m) ) to be the total number of scRNA-seq reads allocated for topic k for cell m and the total read counts for gene g under topic k across all of the M cells without counting the current i^th read for cell m, however, according to the formulas written by the authors, the former excludes all reads from gene g and the latter excludes all reads from cell m.

**Reviewer 3**

Adapted from their prior work MixEHR-Guided, Swapna et al. proposed a guided topic model for cellular deconvolution (GTM-decon). Using single-cell RNA-seq and guided by the cell cluster labels, they estimated a cell type-specific gene expression profile matrix and used it to estimate cellular fractions from bulk gene expression data. While they did a lot of data analyses, the method was not sufficiently presented, and thus it's hard to understand how the cellular fractions were exactly estimated. The method is also not rigorous and comprehensively benchmarked. Below I list some comments that may improve the manuscript.

My main concern is about the clarity of the methods.

1) The notations in Fig 1a do not match that on Page 12. In Fig 1a, there is X_{i,m}, where i denotes the scRNA-seq read count. On page 12, there is only x_{g,m}, where g stands for a gene.

In Fig 1a, there is \beta_k, while on page 12, "a fixed hyperparameter 𝛽 across all K dimensions" was used.

In Fig 1a, there is prior + 0.1, which is not described in Methods.

In Fig 1a, "n = 1, …, Nj; j = 1, …, D." Those notations are not used in Methods. A clear description of the methods with precise notations is needed.

2) In methods, "Given topic 𝑧_{i,m} = 𝑘, we then draw a gene index 𝑥_{g,m} from a G-dimensional categorical distribution: x_{g,m} ~𝐶𝑎𝑡(𝛟_k), where 𝛟_𝒌 follows a G-dimensional Dirichlet distribution with a fixed hyperparameter 𝛽 across all K dimensions: 𝛟_k~𝐷𝑖𝑟(𝛽)." Can you explain how z_{i.m} plays a role in x_{g,m}? Aren't the read counts mapped to each gene?

3) I think the main confusion comes from the fact that there is no idea where the observed scRNA-seq comes into the posterior solution shown on page 13. All seem to be parameters.

4) How sensitive are the results to the hypermeters? E.g., 0.9 was used for a cell assigned to a cell type. Why use 0.9? How does that differ from using 1? If to account for cluster uncertainty, some clustering methods have estimated those multi-type probabilities (e.g., using Dirichlet distribution). Using that estimated information may be more accurate than those arbitrary probabilities of 0.9 and others sampled uniformly from 0.01 and 0.1.

5) While the paper's main focus is estimating cellular fractions from bulk data, it was only briefly described on Page 13, following an algorithm similar to assigning single cells to a topic. However, the single-cell algorithm used prior about cell labels in alpha. How was that used in bulk data? A specific algorithm should be described for bulk data without referring to other references.

The benchmarking is short, on a paragraph on page 4. In Fig 2a, the authors calculated the correlation for each bulk sample across cell types. It is more important to calculate the correlation across samples for each cell type, and also assess the bias. The simulation is a simple combination of single-cell data and would be optimistic. Real data benchmarking is more useful, e.g., Framingham Heart Study collected white blood cell counts and gene expression data and can be used as a good real benchmarking dataset.

In "Preprocessing the reference scRNA-seq data," "all: simple count data preprocessed to remove outliers (highly expressed genes -found in ≥ 80% of cells, lowly expressed genes - found in ≤ 5 cells)."

Did you filter out genes based on the percentage of nonzero expression? How many genes are filtered out? It seems improper to call "all" genes, given some genes are filtered out.

Software computation time and memory requirements need to be discussed.

In Discussion, "Our results suggests that most of the top genes under the cell-guided topics correspond to known marker genes (Figure 3a, Figure 5a)."

I cannot see that most top genes correspond to known marker genes from the two figures. It is also unclear why there are NA in marker legends. In the two marker databases, a gene is either claimed to be a marker or not, right?

The authors need a more thorough literature review. For example, in the Discussion, "The latter allows us to identify cell-type-specific differential expression of genes directly from bulk RNA-seq data (Figure 9). This feature is not available in most approaches developed for cell-type deconvolution, except CIBERSORTx …"

Several other methods can also do this, like CellDMC, TOAST, and bMIND.

The tables in Fig 1 need to be improved. All unassigned probabilities = 0.05.

The paper is too long, and some figures can be condensed and summarized. For instance, the heatmaps of cellular fractions in Fig 2 are hard to compare. Using scatterplots by cell type would make it clearer.

The paper needs more editing. A quick reading would find the following issues.

Page 4, second line, "tends provided"

Page 5, last line, "into into"

Page 10, line 25, "results suggests"

Page 12, second line, ", ."

Some references are duplicated. Ref 21 = 25; Ref 41 = 48.

**Authors’ response**

In summary,

1. We have performed benchmarking on 7 real bulk datasets, where the cell-type proportions are available as the gold standards;
2. We have experimented the hyperparameter for the cell-type-specific topic prior values (i.e., $\alpha$) as well as the hyperparameter for the CTS topic distributions (i.e., $\beta$);
3. We have not only provided clear description of our method in the inference algorithm for the deconvolution but also highlighted our methodological contributions more prominently;
4. We also revised the text and the figures to incorporate reviewers’ suggestions, simplify the presentation, and to adhere to Genome Biology’s formatting guidelines;
5. We created **Additional file 1** that consolidates the Supplementary sections, figures, and tables.

The revised contents are highlighted in the main text with track changes.

# Reviewer #1:

**This study highlights the importance of heterogeneity in studying the progression of several diseases including cancer. It also highlights the high cost, lower throughput and difficulties in sample preparation challenges involved using experimental approaches at the patient population scale motivating the need for computational deconvolution methods such as GTM-decon. The authors claim GTM-decon is a highly efficient and interpretable Bayesian approach for cell-type deconvolution, based on guided topic modeling which learns cell type-specific gene signatures in the form of categorical distributions (i.e., latent topics) from scRNA-seq datasets.**

## Many computational methods have been developed to infer cell type proportions from bulk transcriptomics data including benchmarking of these methods guided by scRNA-seq signatures. While the authors try to make the case that GTM-decon is highly interpretable compared to traditional methods and the fact that single-cell research is increasingly getting cheaper and mainstream, I find the proposed methodology, results and the significance of the entire study limited in justifying this claim among others including innovation, clarity, reproducibility and generalizability in advancing the field.

**Our response:**

Thank you for the critical feedback. We hope that our revision has highlighted our contributions more prominently. We agree with the reviewer that the problem of cell-type deconvolution has been studied extensively in recent literature. However, we brought in two main methodological contributions to the community.

First, to the best of our knowledge, there are few reference-free methods like ours that can infer *de novo* (sub-)cell-type-specific gene expression distributions over *all genes* from a single-cell reference data distribution without marker gene information. Furthermore, our approach can infer multiple topics per cell type in a simple and elegant design via configuring the Bayesian prior hyperparameters for the Dirichlet cell-type mixture variable. Because each cell-type-specific gene expression variable follows a Dirichlet distribution, it enjoys the interpretability of the topic models. In particular, we can directly examine the top genes under each sub-cell-type topic to identify known and novel markers.

Second, the nested phenotype and cell-type guided topic inference allows us to perform a novel style of cell-type-specific (CTS) differential expression (DE) analysis over the bulk samples in a case-control study design. While several methods such as bMIND (Wang et al., Genome Research 2021) and TOAST (Li and Wu, Genome Biology 2019) can perform CTS DE analysis as well, they often rely on existing deconvolution methods to estimate the cell-type proportion from the bulk and therefore its performance depends on the accuracy of those deconvolution methods in use, which have assumptions on their own. In contrast, our GTM-decon performs both deconvolution and DE in a single unified topic-model framework.

We have substantially revised the method description to not only highlight our contributions but also improve its clarity as a standalone section. To further demonstrate the utility of our method, in this revision, we have added benchmark experiments on seven real bulk datasets, where the groundtruth cell-type proportions are available and evaluated the deconvolution performance in comparison with the state-of-the-art methods.

Please see our specific response to each comment below.

## 1. Interpretability as a selling point of the paper needs to be properly defined with clear literature and metric supporting how this feature has been investigated in the field. Furthermore, given that the performance of GTM-decon depends on sensitive labeled scRNA-seq data, whose processing workflow involves challenges such as cell sorting, normalization, gene dropouts, feature selection and transformation, matching cell types during disease progression etc. The weak sensitivity or benchmarking analysis and lack of clarity highlighted below in generating the tumor-immune reference signature data, the poor labeling of the tumor immune cell types and the proposed simulation results based only on high variable genes demonstrates bias in the performance results and interpretability by the method.

**Our response:**

Thank you for the thoughtful feedback. There are two main points to address in this comment.

### (1) Interpretability

From a machine learning perspective, probabilistic models can be thought of as inferring plausible models to explain observed data (Ghahramani, Nature 2015). Topic models are a family of such probabilistic frameworks, which exploit the sparsity of the vocabulary observed in a single document and distills a set of topic distributions that characterize them (Blei et al., JMLR 2003; Blei, ACM 2012). These topics often capture a coherent set of themes based on post-hoc inspection. For instance, the top 5 words with the highest topic probability under the same topic, say “school”, “classroom”, “students”, “teacher”, “classmates”, may suggest a theme of education, and the top 5 words for another topic, say “senate”, “president”, “campaign”, “votes”, “debate”, implies a theme of presidential election.

In our context, model interpretability refers to the ability to understand and explain how the inference of the cell-type mixing proportion is made. To this end, the generative process of the GTM-decon model assumes that each scRNA-seq read comes from a specific cell-type and maps to a specific gene with the probabilities dictated by the cell-type-specific (CTS) topic distribution over all genes. As a result, we can directly examine the inferred CTS gene topic probability distribution for each cell type to make sense of them.

However, the latent topics themselves from a vanilla latent Dirichlet allocation (LDA) are not identifiable, which means we cannot directly associate each topic with a specific cell type without manual post-hoc inspection of the top genes under each topic. This compromises their interpretability. In GTM-decon, we solved this problem by guiding the topic inference with cell-type information. This enables us to identify each topic with exactly one cell type. We can then systematically evaluate the model performance based on its deconvolution accuracy in comparison with the existing methods.

Regarding the interpretability of the inferred CTS topic distributions themselves (i.e., the genes-by-CTS-topics matrix), common metrics in evaluating the quality of the inferred topics include topic diversity (i.e., the uniqueness of the top terms under each topic), topic coherence (i.e., the co-occurence of the top terms among in the same documents) (Mimno et al., EMNLP 2011), and perplexity score (i.e., the negative log likelihood of the held-out data). While quantitative, these metrics are not directly relevant in our context because they don’t convey whether the inferred topic model captures the biological signatures of the cell types. Instead, we performed gene set enrichment analysis to show that the top ranked genes under each cell type are indeed enriched for known markers for the corresponding cell type (**Fig. R1a,b** and **Fig. R2**). This underscores the remarkable interpretability of our model especially because it does not use cell-type markers during the training (i.e., reference-free). In contrast, supervised methods such as Scaden (Menden et al., 2020) (i.e., a feedforward neural network) or multi-class logistic regression are only designed to predict cell-type proportions. Their parameters are not as interpretable as the CTS topic distributions.

Lastly, the extensions of our method to phenotype-guided modeling and nested-guided phenotype-CTS topic inference contribute an substantially additional avenue to interpretability in terms of identifying phenotype-specific gene distributions and CTS differentially expressed (DE) genes with respect to the phenotype states (e.g., breast cancer subtypes) (**Fig. 5** and **6** in the main text, respectively).

**Figure R1. Cell-type-specific topic inference and deconvolution of pancreatic tissue.** Please see the detailed captions in **Figure 3** in the main text of the manuscript.

**Figure R2. Gene signatures of cell-type-specific topics in breast tissue.** [This was **panel a** in the original **Figure 5** and has been moved to **Supplementary Figures (Figure S24)** in the revision] We trained GTM-decon on a scRNA-seq reference of normal breast tissue [43] using 5 topics per cell type, on the set of highly variable genes. Same as Figure 3, the heatmap visualizes the top 20 genes per topic for the 5 main cell types in normal breast tissue (i.e., 25 topics in total for the 5 cell types). The heatmap intensity is proportional to the gene topic probabilities. Whenever available from CellMarkerDB and PanglaoDB, cell-type marker genes are indicated on the left. For the cell types, where marker genes are not available, “NA” were indicated on the left.

### (2) The usage of cell-type labels

We agree that the cell-type labels depend on several technical aspects of scRNA-seq methodology employed as well as the data quality after the preprocessing. However, our model does **not** completely rely on the cell-type labels. Instead, we incorporate the cell-type label information in the form of the *Bayesian prior* of the topic mixture. By default, we set 0.9 to the hyperparameter alpha value(s) for the Dirichlet variable for the CTS topic(s) and randomly sampled values from 0.01-0.1 to the rest of the hyperparameter values for other topics (as illustrated in the revised **Figure 1a**). We also explored the effects of setting different values for the hyperparameter (please see our response to **Comment 5**).

As a result, while the topic mixture value θ_m,k_ corresponding to the observed cell-type label y_m_ = k will have a high expected value, other mixture values will have low but non-zero expected value as well. The final posterior distribution of the cell-type topic assignment depends on both the topic prior and the likelihood on how well they can explain the scRNA-seq data. This is reflected in the cells-by-CTS-topics distribution, where each cell still has non-zero probabilities for the cell types that are not assigned to them (**Figure R3**). Therefore, the cell-type mixture **θ**_m_ reflects the uncertainty of the noisy cell-type label y_m_ for cell m.

**Figure R3.** **Heatmap of cells-by-CTS-topics distributions inferred by GTM-decon during training from the PBMC dataset.** The heatmap shows the inferred cell-type proportions for each cell in the training data as rows and the CTS topics annotated by the corresponding cell types as columns. The values indicate the inferred probabilities for the cell to be represented by each of the CTS topics. The row annotations are colored according to the true cell type label of the training data. We observe that although the highest probability for each cell is assigned to the correct CTS-topic, other topics are also assigned non-zero probabilities.

Moreover, the statistical uncertainty is also reflected in the genes-by-CTS-topics matrix. For example, in **Fig. R1a**, we showed that endothelial, epsilon, and mast cell-types hardly have any clear topic-specific genes, due to lesser training data, and the lower confidence ensures that these cell-types are not spuriously predicted during the deconvolution step (**Fig. R1c and R1e**).

We also acknowledge that reference-based approaches such as CIBERSORTx (Newman et al., Nat Biotech 2019) tend to have more accurate deconvolution accuracy than the reference-free approaches like ours when the carefully constructed set of marker genes are of high discriminative performance. But these methods are either not applicable or have poor performance when the marker genes are not available or less well defined, respectively.

On the other hand, our reference-free approach can automatically infer the gene contributions to each cell-type without relying on the incomplete marker genes information. This also enables identification of novel marker genes. In contrast to hard-thresholded feature selection in other well-known reference-free methods such as TOAST (Li and Wu, GB 2019), our method can express uncertainty by assigning probabilities to each gene in terms of their contribution to each cell-type.

We respond to the comment regarding the tumor-immune next.

**References in the above response:**

1. Ghahramani, Z. Probabilistic machine learning and artificial intelligence. Nature 521, 452–459 (2015).
2. Blei, D. M., Ng, A. Y. & Jordan, M. I. Latent dirichlet allocation. The Journal of Machine Learning Research 3, 993–1022 (2003).
3. Blei, D. M. Probabilistic topic models. Communications of the ACM 55, 77–84 (2012).
4. Mimno, D., Wallach, H. M., Talley, E., Leenders, M. and McCallum, A. Optimizing semantic coherence in topic models. In Conference on Empirical Methods in Natural Language Processing. (2011)
5. Wu, H. TOAST: improving reference-free cell composition estimation by cross-cell type differential analysis. Genome Biology 20, 1–17 (2019).
6. Wang, J., Roeder, K. & Devlin, B. Bayesian estimation of cell type-specific gene expression with prior derived from single-cell data. Genome Res 31, gr.268722.120 (2021).
7. Menden, K. et al. Deep learning–based cell composition analysis from tissue expression profiles. Sci Adv 6, eaba2619 (2020).
8. Newman, A.M., Steen, C.B., Liu, C.L. et al. Determining cell type abundance and expression from bulk tissues with digital cytometry. Nat Biotechnol 37, 773–782 (2019).

## 2. For example, labeling fibroblasts as immune cells show fundamental lack of the understanding of tumor-immune biology. Furthermore, using independent breast and RA tissues from different patients to validate the deconvoluted tumor immune cell types without a clear analysis of between and within variability is bias. There is no clear justification why immune cells from the same tumor microenvironment or tumor region is not used as reference given joint single-cell transcriptional/protein and imaging data can be obtained. It is also not clear how the proposed method performs with missing important cell types in the reference data or how the differential gene analysis takes into account the changes in proportions of cell types between normal and disease states. Furthermore, the proposed approach of simulating bulk from single-cell data lacks statistical or biological validation and the effect of using different forms of normalization and batch corrections for various datasets

**Our response:**

There are multiple great points to address in this comment and we organize our response into five sections.

### (1) Using a tumor-immune scRNA-seq reference for tumor deconvolution

We agree with the reviewer. During our initial analysis, we did not see a tumor-immune scRNA-seq dataset for pancreatic cancer and hence sought to combine pancreatic and immune tissues from different sources. For breast cancer (shown in Figure 6 of the original manuscript), we used a breast cancer specific scRNA-seq reference dataset since it comprehensively covered all breast cancer subtypes.

With a more careful search, we have found a better matched scRNA-seq reference dataset for the target tumor types, including one for pancreatic cancer (Peng et.al., Cell Res, 2019). Therefore, we no longer need to combine datasets from multiple sources and instead utilize these appropriate references for the tumor deconvolution task. Consequently, we have removed the section titled “Deconvolving bulk RNA-seq datasets using multiple scRNA-seq references'' in the **Methods** section.

We also modified the **Figure 4** in the original manuscript and moved it to the **Additional file 1**. In particular, we used only a single reference scRNA-seq data from the matched normal tissues for analyzing the pancreas cancer datasets to draw qualitative comparison with the new results based on the better matched reference (**Figure R4**). In the interest of space, we also moved **Figure 6** (which was based on breast cancer scRNA-seq reference) to the **Additional file 1**.

We agree that if we were to combine multiple external references, inter and intra study variability needs to be rigorously assessed, which is tangential to the aim of the current study and can be explored as a separate topic in future work.

The modified section is rewritten in the manuscript: *“****Deconvolving pancreatic cancer transcriptome s identified tumor cell type origin****”*

**Figure R4. Deconvolution of bulk RNA-seq samples for pancreatic cancer from TCGA-PAAD using scRNA-seq reference from pancreatic cancer subjects.** Please refer to **Fig. 4** caption in the main text for detailed description.

We also acknowledge the reviewer’s point that several datasets of different modalities are currently available for many tissues (Hao et al.,Cell, 2015; Efremova and Teichmann, Nat. Methods 2020) and indicate this in the manuscript, and note that our model is not limited to single cell RNA-seq, and can be extended to other modalities (e.g., joint RNA and protein data) – as future directions (as noted in **Discussion**).

### (2) Impacts of missing cell types

Indeed, it is possible that some cell types in the bulk data are not present in the single-cell reference data that the model is trained on. To address this, we directly trained an unsupervised topic model on the sparsified bulk data, and denoted these topics as “*de novo* or bulkRS topics”. We note that these topics capture the most abundant cell types (**Figure R4**), which were annotated with the aid of the CTS topics. To examine the impact of missing cell types, we analyzed the effect of deconvolution using two scRNA-seq datasets with two different sets of cell types, namely human blood cells (HBC) (GSE149938; Xie et al., Natl Sci Rev, 2021) and peripheral blood mononucleocytes (PBMC) (Single Cell Portal / GSE132044; Ding et al, Nat Biotech, 2020) (**Additional file 1: Table S2**). We deconvolve purified bulk from immune cells using these references and compare their results (**Figure R5**). Interestingly, HBC doesn't have Basophils, their signal is split between HSPCs (generic progenitors), and Neutrophils (another granulocyte cell type); the PBMC2 reference does not have HSPC cell type, the signal is distributed across all cell types. Therefore, when some cell types are missing, GTM-decon is still able to either find the closest match to the missing cell type or appropriately express its uncertainty. We added a more detailed analysis in subsection “**Evaluation of deconvolution of real bulk with ground-truth cell-type proportions**” under **Results**.

**Figure R5. Evaluation of missing cell types in reference.** We inferred cell-type proportion over purified bulk samples of immune cell types (GEO access: GSE107011) based on CTS topics inferred separately from two distinct scRNA-seq references (HBC - Xie et. al., Natl. Sci. Rev, 2021 and PBMC - Nat Biotech, 2020) with incomplete cell types with respect to the target bulk samples. We also experimented using either all genes or highly variable genes (HVG). The heatmaps show a qualitative comparison of the combinations of the reference and gene selection while using HVG, where the rows are the bulk samples with color legend indicating their cell types and the columns are the cell types from the scRNA-seq HBC reference (left panel) and PBMC2 reference (right panel). The same figure is shown as **Additional file 1**: **Figure S16**.

### (3) How does cell-type proportion change affect the differential expression analysis

As described in the revised “**Identifying CTS DE genes from bulk transcriptomes from nested-guided topics**” subsection in **Methods**, our model infer cell-type proportions and phenotype-specific gene topic distributions simultaneously on a set of the bulk samples, thereby capturing the global changes in gene expression for the same cell-type across phenotypes. The change could be caused due to proliferation of that cell type in a phenotype as well as higher expression in one of the phenotypes for same cell type proportions, and both cases would be biologically relevant.

In our CTS DE analysis on breast cancer subtypes (**Figure 6**), we identified CTS DE genes across the Basal, Basal myoepithelial, Luminal (1-1, 1-2, 2) cell types via the changes of the global CTS topics for Basal versus ER+ subtypes.

We then examined how the cell-type proportions differ between Basal and ER+ subtypes at the level of each individual breast tumor sample (i.e., $\theta_{j}$ for each sample $j\in\{1,...,D\}$) (**Figure R6**, also **Additional file 1**: **Figure S26**) shows the distributions of cell-type proportions inferred for the two subtypes by GTM-decon. We observe that several of the cell types show significant differences in proportions; however, Luminal_2 exhibits the largest difference between the two subtypes, with an increase in the ER+ subtype (**Figure R6**). Notably, the same cell type also exhibits the largest number of DE genes (**Figure R7**).

We note that while this analysis provides pointers on the cell-types that are highly associated with the cancer subtypes, it does not directly reveal the causal effect of the cell-type proportion changes on the individual CTS DE genes. The latter one is an exciting direction to be explored in future study.

**Figure R6:** **Differential analysis of cell-type proportions by breast cancer subtypes of Basal and ER+.** The box plots show the distribution of inferred cell type proportions for the five cell types in terms of the breast cancer subtypes – Basal and ER+. The box and the whiskers in each boxplot indicate the 25%-75% quartile and min-max of the evaluation scores for each of the samples. Statistical significance calculated using the Wilcoxon test suggests that the four of the cell types show significant differences in proportions across the subtypes, with Luminal_2 exhibiting the highest difference. The same figure is shown as **Additional file 1**: **Figure S26**.

**Figure R7:** The heatmap shows the 630 cell-type specific DE genes identified by GTM-decon. The number of DE genes identified in each of the cell types is listed on the side. The same figure is shown as **Fig. 6e** in the main text.

### (4) Validation of simulated bulk data in comparison with the real bulk data

We examined whether simulated bulk samples preserve the gene expression pattern as in the real bulk using paired bulk and single cell RNA-seq data from the same individuals. Indeed, we observe a high correlation between the log-transformed gene expression of each artificial bulk pancreatic sample and the gene expression from the real bulk RNA-seq data collected from the pancreatic tissue of the same subject. The correlations are consistently high (0.927-0.963) across all 7 individuals (3 are healthy and 4 are T2D subjects) (**Figure R8**). Similar validation of the artificial bulk versus real bulk was done by Wang et al., Nat Comm. (2019) (i.e., the MuSiC paper), and our data are consistent with theirs (please refer to their Supplementary Figure 1b). Moreover, we have also added a new set of benchmark experiments on evaluating deconvolution accuracy on real bulk samples using the groundtruth cell-type measured by flow cytometry. Please refer to our next response as well as response to **Comment 4**.

**Figure R8.** Comparison of log transformed relative abundance levels between real bulk tissue RNA-seq data and artificially constructed bulk RNA-seq data for the same subject. Single-cell and bulk tissue RNA-seq data are both from Segerstolpe et al. Each dot represents a gene and the red line is x=y. The same figure is shown as **Additional file 1**: **Figure S9**.

### (5) Effect of normalization and batch correction

In our original manuscript, we had considered the effects of normalization on deconvolution performance by considering two normalization strategies: normalizing the counts by the total sum in each cell, and scaling it by multiplying by 10,000 (normr), and log-transformation - by taking the log of the normr values (normr-log1p). Although we had utilized them, we did not show their performance except for one figure (Figure S2 in the original manuscript).

In the revised version, we systematically evaluated their performance on a real bulk dataset of whole blood with known ground truth proportions measured by the flow cytometry data (**Figure R9**). We also experimented the normalization effects on 3 different gene selection strategies namely all genes, preprocessed (pp) genes (i.e., removing frequently expressed genes in more than 80% of the cells and infrequently expressed genes fewer than 5 cells), and highly variable genes (hvg) (processed by scanpy). We observe that raw counts perform the best for all the gene sets, whereas normalization decreases the performance to varying degrees for different gene selection strategies. This is consistent with the underlying discrete categorical distribution assumption by the topic model. The methods for normalization as well gene selection were now detailed in the subsection “**Preprocessing the reference scRNA-seq data**” under **Methods**.

As for the batch correction, since we no longer integrate multiple scRNA-seq data from different sources of studies as described above, it has a lesser effect on our results. We agree that additional batch correction procedures need to be considered when leveraging multiple scRNA-seq references and recognize the benefits of having a larger number of cells and cell types by doing so. We will explore that in future work.

**Figure R9. Effect of different normalization strategies for whole blood RNA-seq bulk dataset.** The boxplots show the deconvolution accuracy in terms of Pearson correlation between ground-truth cell-type proportions estimated by flow cytometry for each sample and the inferred cell-type proportions by GTM-decon (y-axis). The box and the whiskers in each boxplot indicate the 25%-75% quartile and min-max of the evaluation scores for each of the samples. Deconvolution performance is shown for different models using a varying number of topics (1 to 5) to model each cell type. The boxplots reveal that raw counts perform the best for all the gene sets, whereas normalization decreases the performance to varying degrees for different gene selection strategies. The same figure is shown as **Figure S3** in the **Additional file 1**.

**References in the above response:**

1. Peng, J. et al. (2019). Single-cell RNA-seq highlights intra-tumoral heterogeneity and malignant progression in pancreatic ductal adenocarcinoma. Cell research, 29(9), 725–738.
2. Hao, Y., et al. (2015). Integrated analysis of multimodal single-cell data. Nature 521, 452–459.
3. Efremova, M. & Teichmann, S. A. Computational methods for single-cell omics across modalities. Nat Methods 17, 14–17 (2020).

**Other Issues:**

## 3. In page 6 line 14-16, the authors use all gene expression profiles as input to build a reference model. By all genes do they mean all the genes in the original raw data? If not how were these genes selected?

**Our response:**

We agree that the terminology of “all genes” is misleading. We apologize for the confusion. In our original manuscript, we had used gene expression profiles from a leniently pre-processed gene set, which excludes likely housekeeping genes (i.e., genes that expressed in >=80% cells) as well as infrequently expressed genes (i.e., genes that expressed in < 5 cells). We now refer to this set as **pre-processed (PP) genes** in the manuscript. These genes correspond to ~60% to ~80% of all genes, depending on the datasets (**Table R1,** also **Table S3** in the Supplementary **Information**). In the revised version, along with the PP genes, we also consider *the entire set of genes* (without any pre-filtering) in the scRNA-seq reference data, and refer to that as the “**all genes**” set in the manuscript. We have modified the text in the “**Preprocessing the reference scRNA-seq data**” section under the **Methods** section to elaborate on the different gene sets and their transformations.

**Table R1** (**Table S3**): Summary of gene selection for scRNA-seq datasets

| **Dataset** | **Total genes** | **PP genes** | **HVG** | **%PP genes** | **%HVG** |
| --- | --- | --- | --- | --- | --- |
| **Segerstolpe** | **26178** | **17486** | **5027** | **66.8** | **19.2** |
| **Baron** | **20130** | **15502** | **1667** | **77** | **8.28** |
| **Breast – Normal** | **33694** | **20721** | **2060** | **61.5** | **6.11** |
| **Breast - Cancer** | **29736** | **26169** | **2355** | **88** | **7.92** |
| **PBMC2** | **33693** | **20830** | **8225** | **61.8** | **24.4** |
| **HBC** | **19815** | **17577** | **4389** | **88.7** | **22.1** |

## 4. On figure 2a, the authors claim GTM-decon performs better than other models on the Pancreas and Breast Tissue datasets in terms of both Spearman R and Cross Entropy and conferred comparable performance on the RA Synovum dataset which also seem to produce smaller variance for both the pancreatic and breast tissue datasets. The better performance observed is mostly breast and the spread of points are overlapping in these applications. Comparison of multiple datasets is needed to support such superior performance of GTM-decon including the validation of ground truth proportions.

**Our response:**

Thank you for this excellent point. To further establish the deconvolution accuracy of our method, we have undertaken benchmarking on 7 real bulk datasets from 3 tissues for which the groundtruth cell-type proportions are available (**Additional file 1**: **Table S2**):

a) Three bulk RNA-seq datasets of peripheral blood mononuclear cells (Whole Blood, PBMC S13 cohort, SDY67), with ground truth proportions calculated from flow cytometry
b) Two bulk RNA-seq datasets of cell-sorted purified immune cells (GSE107011, GSE6465)
c) Bulk RNA-seq samples from human brain tissue of a subsample from the Religious Orders Study and Memory and Aging Project (ROSMAP) study, for which ground-truth cell composition information is available from immunohistochemistry.
d) A bulk RNA-seq dataset from pancreatic tissue for 7 individuals, whose scRNA-seq gene expression profiles are also available (E-MTAB-5060 and E-MTAB-5061). The cell-type proportions in the scRNA-seq are used as a surrogate for cell-type proportions in bulk.

We compared the performance of GTM-decon using three gene selection strategies (ALL, PP, HVG) on these datasets with other existing deconvolution methods - MUSIC, CIBERSORTx, BSEQ-sc, BISQUE (**Figure R10**, also **Figure 2** in main text, **Table R2**). We evaluated deconvolution performance on sample deconvolution using Spearman R and Root Mean Square Error (RMSE), by comparing the inferred proportions for the matching cell types against the ground truth proportions for each sample. GTM decon conferred on par or superior performance compared to the existing methods for all the datasets (**Figure R10a**, also **Fig 2a** in main text).

We also undertook an additional analysis - by evaluating deconvolution performance on individual cell types (**Figure R10b**, also **Fig 2b** in main text). We observe that GTM-decon performs distinctly better than the other methods for deconvolving each cell type in the PBMC bulk samples (SDY67) (**Figure R10b**, also **Fig 2b** in main text), and comparably for other datasets (**Figure S11**-**S15** in **Additional file 1**).

Additionally, we evaluated the deconvolution accuracy for purified bulk RNA seq data of immune cells (GEO accession numbers: GSE107011 and GSE64655; **Table S2**) using two different independent references HBC and PBMC2 (**Table S1**). Overlapping cell types were used to evaluate the performance on the purified bulk samples, whereby the highest deconvolution proportion was used as the predicted cell type label for computing the prediction accuracy. GTM HVG achieved the highest accuracy across all four experiments (**Figure R10c**, also **Fig. 2c** in main text). Detailed explanation of the results is provided in the section “***Evaluation of deconvolution of real bulk with ground truth cell type proportions***” under **Results**.

**Table R2.** Spearman correlation between the ground-truth and inferred cell-type proportions on real bulk samples.

| **Method** | **Ref: PBMC, Bulk: PBMC-1** | **Ref: PBMC, Bulk: PBMC-2** | **Ref: Blood Cells Bulk: Whole Blood** | **Ref: Frontal Cortex, Bulk: Prefrontal Cortex** | **Ref: Pancreas, Bulk: Pancreas (LOOCV)** |
| --- | --- | --- | --- | --- | --- |
| **GTM-All** | **0.742557** | **0.69246** | 0.366667 | **0.831707** | 0.570884 |
| **GTM-PP** | 0.704029 | 0.535714 | 0.391667 | 0.802439 | 0.786839 |
| **GTM-HVG** | 0.64798 | 0.386905 | **0.875** | 0.109756 | **0.859543** |
| **BSEQ-sc** | 0.057754 | 0.202781 | 0.400000 | 0.501448 | 0.763868 |
| **CIBERSORTx** | -0.041038 | 0.357156 | -0.278897 | 0.827131 | 0.758431 |
| **MuSiC** | 0.309026 | 0.193214 | 0.862283 | 0.762179 | 0.801137 |
| **BISQUE** | N/A | N/A | -0.116667 | N/A | 0.583751 |

**Figure R10** (**Fig. 2** in the revised main text). Evaluation of deconvolution performance on real bulk data. Please refer to figure caption for Figure 2 for detailed description.

## 5. GTM-decon uses a strong prior (0.9) to anchor each topic to a specific cell type and subsequently guide the inference of the global topic distributions over genes to automatically prioritize cell-type marker genes. What happens if the prior is varied from (0.6-0.8) which is still better than chance.

**Our response:**

We thank the Reviewer for raising this important point. We experimented with varying the values of the priors from 0.6 to 1. We also derived a data-driven prior from a multi-class logistic regression (MCLR) model that was trained to predict cell types of each cell. Specifically, from the fitted MCLR model, we use its fitted values in terms of the predicted probabilities as the prior values for each topic. The fitted values for all the correct cell types are above 0.8, suggesting a high confidence of the MCLR fit.

We experimented these prior settings on several datasets:

a) On two bulk RNA-seq datasets of cell-sorted purified immune cells, GSE107011 (114 samples) and GSE64655 (48 samples), we evaluated the effect of varying topic prior as deconvolution accuracy (**Figure S4**). **Figure R11** shows the deconvolution accuracy derived from two different scRNA-seq datasets as reference using all genes, and highlights that the accuracy is largely similar while varying the priors from 0.6-1, as well as while using priors derived from multi-class logistic regression. The results are similar when using different gene sets as features - all genes, preprocessed genes, highly variable genes (**Additional file 1**: **Figure S4**). Since the prior of 0.9 also enables the modeling of uncertainty, we choose to continue with this prior as the default. Visualizing the results as heatmaps confirms that varying the topics recovers a similar pattern of clustered samples, largely clustered based on their primary cell type (**Figure S5**).

b) On a bulk RNA-seq dataset of whole blood samples of 12 patients with ground truth cell proportions determined by flow cytometry, we evaluated the effect of varying topic prior on deconvolution by pearson correlation between ground truth cell type proportions and the corresponding deconvolved proportions by GTM-decon (**Figure R12**).

These results show that GTM-decon is robust to the different prior values assigned as long as the matching CTS topic prior values are greater than values for the rest of the topics (i.e., 0.01-0.1).

**Figure R11**. **Effect of varying CTS topic prior on deconvolution accuracy in purified immune bulk RNA-seq samples for GSE107011, GSE64655.** The bar plot shows the deconvolution accuracy (y-axis) as the % purified immune bulk RNA-seq samples whose highest deconvolved CTS topic proportion corresponded to its true cell type when using *all* genes as features. The plots are shown as a variation of topic prior from 0.6 – 1, as well as topic priors derived from a multi-class logistic regression model fitting the scRNA-seq data to its cell types (1topic_MCLR), for all the gene sets evaluated in the study. The bar plots reveal that the method is robust to variation in topic prior. Plots for the different gene selection strategies are shown in **Figure S4**..

**Figure R12 (Figure S6). Effect of varying CTS topic prior on deconvolution performance in real bulk datasets with ground truth proportions.** We evaluated the variation in two bulk RNA-seq datasets with ground truth proportions - WholeBlood and GSE107011. The box plots show the deconvolution accuracy in terms of Pearson Correlation between ground truth cell type proportions estimated by flow cytometry for each sample vs. deconvolved cell type proportions inferred by GTM-decon (y-axis). The box and the whiskers in each boxplot indicate the 25%-75% quartile and min-max of the evaluation scores for each of the samples. Deconvolution performance is shown as a variation of topic prior from 0.6 – 1. The bar plots reveal that the method is largely robust to variation in topic prior, with all genes and highly variable genes performing better than pre-processed genes.

## 6. More detail about the harmonization of both pancreatic tissue and immune tissue to the same numerical scale needs to be provided with more sensitivity analysis to support the results.

**Our response:**

In the original manuscript, we had detailed in the **Methods** section under the sub-section “Deconvolving bulk RNA-seq datasets using multiple scRNA-references”. We did not directly combine the two different scRNA-seq reference datasets arising from different sources. Instead, we inferred gene-topic distributions separately for each of the datasets. This mitigated the batch effects arising from directly combining the two datasets based on their raw data.

While this approach is still viable, a simpler solution is to identify the most appropriate scRNA-seq reference datasets for the target bulk tumor datasets to be deconvolved. In the revised version of this manuscript, we no longer combined CTS topics from different datasets, since we have found the scRNA-seq reference datasets that match the tumor-immune environments of the target bulk tumor samples. These scRNA-seq references constitute both the cell types of the tissue and immune cells in the specific tumor environment.

Specifically, we used the Peng et. al (Peng et.al., Cell Res, 2019) scRNA-seq dataset as the reference dataset for deconvolving pancreatic cancer samples (**Fig. 4** in the main text), and Wu et. al (2021) breast cancer scRNA-seq dataset as the reference dataset for deconvolving breast cancer samples **(Additional file 1**: **Figure S23**).

The results of these analyses are described under the sections “**Deconvolving pancreatic cancer transcriptomes identified tumor cell type origin**” and “**Deconvolving breast cancer transcriptomes reveal ed subtype specific markers**” , respectively.

**Reference:**

1. Peng, J. et al. (2019). Single-cell RNA-seq highlights intra-tumoral heterogeneity and malignant progression in pancreatic ductal adenocarcinoma. Cell research, 29(9), 725–738.
2. Wu, S. Z. et al (2021). A single-cell and spatially resolved atlas of human breast cancers. Nat Genet 53, 1334–1347.

## 7. In figure 4a the authors suggest that the bulkRS topics and the reference capture complementary information. What does this mean biologically?

**Our response:**

This is related to Comment 2 in that some bulk samples may contain cell types that are not present in the scRNA-seq reference data. Although our GTM can infer sub-cell-type topics from the reference scRNA-seq data (i.e., multiple topics per cell type), it may still not capture all the cell types in the bulk, either due to technical and/or biological factors. On the other hand, the bulk dataset itself can serve as a source for identifying de novo cell types not observed in the scRNA-seq reference.

Therefore, we sought to leverage both scRNA-seq reference profiles and bulk RNA-seq data to identify cell type proportions in the bulk data. However, classical topic modeling on bulk RNA-seq data results in topics that are not guaranteed to capture cell-type specific information. Comparing the CTS topics inferred by GTM-decon from scRNA-seq reference with the topics inferred by LDA from bulk RNA-seq modeling (i.e., *de novo* bulkRS topics), shows that the *de novo* bulkRS topics are consistent with most of the major cell types in the scRNA-seq reference (as shown in **Fig. 4b, Additional file 1**: **Figure S22a, Figure S23b)** but slightly less so when only the scRNA-seq reference was based only on the healthy tissue samples **(Additional file 1**: **Figure S22a)**.

Furthermore, we estimated whether variation in CTS topic mixture and *de novo* bulkRS topics are indicative of survival time. To explore the marginal effect of individual cell type proportions on survival, we performed Kaplan-Meier analysis (**Figure R13**). We observe that among the cell types, Endocrine cell type and Ductal cell type 2 exhibit significant hazard ratios. However, several *de novo* bulkRS topics that do not align well with CTS topics (such as Topic5 and Topic1) exhibit significant effect sizes on survival (**Figure R13**). These analyses not only reiterate the usefulness of using CTS topics in conjunction with *de novo* bulkRS topics to enable their interpretation but also underscores the importance of *de novo* bulkRS topics in capturing features of clinical significance that cannot be captured only using the CTS topics.

**Figure R13:** Survival analysis of the CTS and de novo topics using inferred cell type proportions . The 174 subjects were divided into two groups based on K means cluster with K set to 2 (not to be confused with the K cell type s or topics) Kaplan Meier curves were generated for these groups and compared using log rank test. The plot shows the log10 (p-value) from the log rank test for all the CTS and de novo topics, in decreasing order of significance. Also available as **FIg 4c** in the main text.

## 8. They claim "GTM-decon is not sensitive to different reference scRNA-seq datasets from the same tissue even if they were generated from different platforms (e.g., Smart-seq and Drop-seq)". Making such a statement from a small replicate analysis is bias given that several studies have shown that different protocols are optimized for different scientific objectives and differ in terms of number of sequenced genes produced.

**Our response:**

We agree with the reviewer and revised the text in the following way:

“*Phenotypic differences between healthy and diabetic patients were captured even when the scRNA-seq reference datasets were generated from different platforms (e.g., Smart-seq and Drop-seq)* (**Figure 3d, f**).”

## 9. It is hard to follow the model description and annotations in the caption of Figure 1 with the figures.

**Our response:**

Thank you for the comment. We took it seriously and substantially revised all sections involving the method description, including “GTM-decon model overview” under **Results**, the first five sections that detail the GTM-decon algorithm under **Methods**, and the **Figure 1** caption.

## 10. Furthermore, the instructions in the GitHub link provided with python code is difficult to follow. Generating a single script to test simple examples will help. It is not possible to know if the results presented can be reproduced or not.

**Our response:**

We have reformatted the GitHub page and streamlined the process. We provided a vignette in a Jupyter notebook with examples that can accept H5AD or DataFrames as input, and a Python wrapper around the GTM-decon C code for ease of use. The vignette can be accessed at: <https://github.com/li-lab-mcgill/gtm-decon/blob/main/vignettes/tutorial.ipynb>.

The tutorial.ipynb Jupyter Notebook walks the user through the use of the GTM-decon python wrapper with a simple test example dataset. The input reference single-cell data can be formatted as either a DataFrame, with the rows representing cells, and columns representing genes, with an additional column containing cell-type labels; or as a cells-by-genes AnnData object (h5ad format), which contains the gene labels in the AnnData.vars and the cell-type labels in the AnnData.obs. The bulk data can also be formatted either as a genes-by-samples DataFrame (each row represents a gene, each column represents a bulk sample) or as a samples-by-genes AnnData object, where the sample names are in AnnData.obs and the genes are in AnnData.vars.

The notebook includes the initialization and setup of GTM-decon, the formatting of input data expected, the model training, and bulk tissue cell-type prediction. Additional Python scripts illustrating the use of GTM-decon can also be found in the vignettes directory as well.

The fully documented Python wrapper can be accessed at: <https://github.com/li-lab-mcgill/gtm-decon/blob/main/PythonWrapper/GTM_decon.py>.

Regarding the reproducibility of the results reported in our manuscript, given the public datasets listed in **Table S1** and **Table S2**, users should be able to generate the results reported in our paper. We also show that our model is robust to the setting of the hyperparameters (**Additional file 1**: **Figures S4-S7**). We also provided scripts that can generate **Fig. 3a** and **3c** using the Segerstolpe scRNA-seq reference pancreas data (**Table S1**) and Fadista Pancreas bulk data for Type 2 Diabetic subjects (Fadista et al., 2014). These are available at <https://github.com/li-lab-mcgill/gtm-decon/tree/main/post_processing/>. The script Fig3a.R inputs the gene-by-topic probability distributions (i.e. the matrix ϕ) inferred from Segerstolpe scRNA-seq reference modeled using 5 topics per cell type, and visualizes for the top 20 genes in each topic, along with the annotations of marker genes from CellMarkerDB and PanglaoDB. The script Fig3b.R inputs the inferred samples-by-CTS topic distribution for the real bulk data from the cohort of 89 donors in Fadista et.al, and visualizes the cell type proportions in the context of the phenotypes. The required input files and expected output files for these scripts are provided in the <https://github.com/li-lab-mcgill/gtm-decon/tree/main/data/> directory to enable comparison.

## Minor concerns

## 1. The authors use the words "guided topic modeling ", "topic" or "phenotype-specific topic" throughout the text without a clear definition especially given these are not standard methodology in the field.

**Our response:**

We have clearly defined these terms in the first subsection “**GTM-decon model overview**” in **Results**.

## 2. Full definitions of MixEHR-Guided model including MixEHR and sureLDA models should be defined

**Our response:**

We have expanded the abbreviations in the subsection “GTM-decon overall algorithm” under the **Methods** section as follows (highlighted in italics):

“*We adapted GTM-decon from the MixEHR-Guided (Mixture of Electronic Health Records – Guided) [ref] (which was in turn inspired by MixEHR (Mixture of Electronic Health Records) [ref] and sureLDA (Surrogate-guided ensemble Latent Dirichlet Allocation) [ref]) to model gene expression from scRNA-seq data.*”

## 3. It seems figure 1b-c not referenced in the text

**Our response:**

Thank you for this comment. In our original manuscript, **Fig. 1b** and **1c** were referenced later in the text, as they refer to derived applications based on the main application. We now refer them in the first subsection “**GTM-decon model overview**” under **Results** the following description.

## 4. There are some typo omissions here and there; Line 5-9 page 6 "cell type tends provided better performance", …. We also assessed the effects of using different genes selection strategies, Line 34-36 page 10 …Notably, the pancreas adenocarcinoma other subtype is predicted to originate from alpha cells, as observed in humans, Line 19-21 Page 13 needs review.

**Our response:**

We thank the reviewer for bringing this to our notice. We have corrected the typos as follows (highlighted in red):

“*While in most of our applications we find that the single-topic per cell type is sufficient to capture most of the variation of gene expression, the GTM-decon with multiple topics per cell type tends to provide better performance in terms of deconvolution accuracy on simulated bulk RNA-seq data using real scRNA-seq data.*

*We also assessed the effects of using different gene selection strategies and different data transformations on the deconvolution performance.*

*Notably, the pancreas adenocarcinoma “other” subtype is predicted to originate from alpha cells, which was also observed during deconvolution with human reference data.*”

We also carefully proofread the manuscript to make sure the manuscript is free of other typos.

# Reviewer #2:

**The authors present a new Bayesian method "GTM-decon" based on guided topic modeling, which aims at automatically learning cell-type-specific gene signatures from scRNA-seq data to deconvolve bulk RNA-seq data. They validate their method through simulated and real data experiments. Interestingly, they also extend their method to learn phenotype-guided topics and identify cell-type-specific differentially expressed genes. I have a few questions about this work.**

## 1. The authors use a guided topic modeling mechanism to learn cell-type-specific gene signatures. What are the advantages of using this mechanism over calculating average gene expression from scRNA-seq data directly?

**Our response:**

Thanks for the question. First, the cell-type-specific (CTS) topic distributions capture the relative importance of the genes with respect to each cell type. If we were to take the average of gene expression over the cells of the same cell type, the top genes will be predominantly the highly expressed genes including housekeeping genes rather than the cell-type marker genes. Second, we consider the observed cell-type labels in the scRNA-seq are noisy due to the technical and biological variations and the data preprocessing and use the cell-type topic mixture to express the statistical uncertainty of the cell-type labels. Therefore, the model is robust to mislabelled cells. Third, the average CTS gene expression cannot be directly used as probability topic distribution in the subsequent deconvolution using the topic modeling mechanism. Lastly, as one of the 3 main contributions highlighted in the revised Introduction section, we can infer multiple sub-cell-type topics for the same cell type. This can not be obtained by averaging the gene expression.

## 2. The authors assess the effects of using different data transformations on the scRNA-seq data. However, a more common transformation is ignored by the authors, which is to normalize counts first and then perform logarithmic transformation. In addition, after preprocessing the data, the data will no longer be integers, what way do the authors use to make it fit the topic model?

**Our response:**

We thank the reviewer for pointing this out. We had indeed performed normalization first followed by scaling (i.e., multiplying the normalized count by a factor of 10000, a common scaling factor, which also prevents the issue of taking a log of fractional values from resulting in negative numbers) before taking the logarithmic transformation. We then rounded the resulting values to the nearest integer to make the input suitable for topic modeling.

In the original manuscript, we indicated this procedure in an inaccurate order as log1p_normr, which implies “taking log followed by normalization”. We have corrected this in the manuscript, and also expanded the subsection “**Processing the reference scRNA-seq data**” under **Methods** to detail gene selection and transformations that we have experimented, as follows:

“*The gene expression profiles of scRNA-seq data were used as training data. Since the performance of GTM-decon depends on how the scRNA-seq count data are processed, we explored different gene selection and transformations of the scRNA-seq data. Specifically, three sets of genes are analyzed: a) all genes b) preprocessed genes c) highly variable genes. Here,*

*i) all genes: raw count data;*

*ii) preprocessed genes: simple count data preprocessed to remove uninformative genes (frequently expressed genes – found in ≥ 80% of cells, infrequently expressed genes – found in ≤ 5 cells);*

*iii) highly variable genes: identified using the highly_variable_genes (HVG) function of scanpy [44].*

*For each of these gene sets, the following variations are considered:*

*i) normr (all_normr / pp_normr / hvg_normr) – normalize counts (while excluding highly expressed genes for calculating the normalization factor) as counts divided by the sum of counts per cell multiplied by a scaling factor of 10,000 (a commonly used factor for scRNA-seq data [47], and round the values to their nearest integer to make the input suitable for topic modeling.*

*ii) normr_log1p (all_normr_log1p / pp_normr_log1p / hvg_normr_log1p) : log-transform the normalized counts in ii), and round the values to their nearest integer to make the input suitable for topic modeling.*”

## 3. The prior distribution of Φ_k is a Dirichlet distribution with a fixed hyperparameter β. How do the authors set this hyperparameter? How would different settings of it affect the results?

**Our response:**

By default, we set the hyperparameter β to 0.01. Conceptually, we use a small value as default to mainly let the scRNA-seq data likelihood drive the CTS topic distribution Φ. Since $\phi_{g,k}\propto\beta+n_{g,.,k}$, where $n_{g,.,k}=\sum_{m=1}^{M} \sum_{i=1}^{N_{m}} {[x_{i,m}=g]\gamma}_{i,m,k}$ for M cells and N_m_ reads for each cell, with sufficiently large number of cells and sequencing depth, $\beta+n_{g,.,k}\approx n_{g,.,k}$. Since $\phi_{k}$ follows a G-dimensional Dirichlet prior $\phi_{k}\sim Dir(\beta)$, small hyperparameter value for $\beta$ allows encourages high density at the corners of the Dirichlet simplex, which can result in high probabilities over only a small subset of the genes (i.e., marker genes) and therefore low probabilities over the vast majority of the remaining genes (i.e., non-marker genes) (since $\sum_{g=1}^{G} \phi_{g,k}=1$). This reflects our Bayesian prior belief that only a small set of the genes are markers for each cell type (although we don’t enforce relatively high prior values for a particular set of known marker genes due the incomplete knowledge of the cell-type markers and leave it to future work). As suggested by the Reviewer, we experimented with the values of the $\beta$ hyperparameter from 0.0001 to 1. We assessed the effects of varying the hyperparameter on real bulk data from 12 whole blood samples with ground truth cell type proportions determined by flow cytometry (**Figure R14**). The results show that GTM-decon is robust to the different values of the $\beta$ hyperparameter, and we continue using our default value of 0.01.

**Figure R14. Effect of varying hyperparameter beta on deconvolution performance in GSE107011.** The hyperparameter beta was varied from 0.0001 to 1. The effect on performance was evaluated on true bulk RNA-seq with known ground-truth proportions. The box plots show the deconvolution accuracy in terms of Pearson correlation between ground-truth cell type proportions estimated by flow cytometry for each sample and the inferred cell type proportions by GTM-decon (y-axis). The box and the whiskers in each boxplot indicate the 25%-75% quartile and min-max of the evaluation scores for each of the samples. The plots suggest that the method is robust to variation in hyperparameter beta. This is a part of the **Figure S7** in Additional file 1.

## 4. Could the authors provide a more detailed description on how to use genes-by-topics estimates to deconvolve bulk RNA-seq data? For example, does bulk RNA-seq data need to be preprocessed? What is the data generative process for the gene expression of each sample in bulk RNA-seq data? What are the specific calculations and formulas?

**Our response:**

We thank the reviewer for raising this important technical point. We expanded the section “**Inferring mixing cell-type proportions in bulk transcriptome**” in the **Methods** section to provide a detailed description of the deconvolution algorithm.

## 5. When using standard LDA for deconvolution, how do authors determine whether a detected topic corresponds to a known cell type or a new cell type? Are there any metrics? Since there is no prior information, the detected topics may be a combination of multiple cell types, or may be independent of cell types. How to better explain these topics?

**Our response:**

We use standard LDA only to infer de novo topics from the bulk RNA-seq but not for deconvolution. Otherwise, as the Reviewer mentioned, we would have had unidentifiable topics from the standard LDA. The purpose of running the standard LDA on bulk data is to detect any topics that are not captured in the CTS topics inferred from the single-cell RNA-seq reference data. For deconvolution, we fix the topic distributions as described above. We hope that by clarifying the above deconvolution algorithm, this point has been made more clear.

## 6. When deconvolving bulk RNA-seq datasets using multiple scRNA-seq datasets, the authors first estimate the genes-by-topics matrix independently for each dataset and then combine them. Compared with combining the multiple datasets first and then estimating the genes-by-topics matrix, what are the advantages of this way?

**Our response:**

As we described in the **Methods** section under the sub-section “Deconvolving bulk RNA-seq datasets using multiple scRNA-references” in the original manuscript, in order to combine two different scRNA-seq datasets arising from different sources, we infer gene-topic distribution separately for each of the datasets to avoid batch effects arising from combining the two datasets based on their raw data, and then harmonize them to be on the same scale. However, in the revised version of this manuscript, we do not use this approach anymore and have removed the above subsection. That is because we have found a matching scRNA-seq dataset from pancreas and breast tumors for deconvolving the target bulk pancreas and breast cancer transcriptomes, respectively (**Figure 4** and **Figure S23**). These scRNA-seq references comprise of both the cell types of the tissue and immune cells in the specific tumor environment. Therefore, they are better matching references compared to combining the normal and immune references, whose batch effects and statistical variations need to be more thoroughly analyzed before being combined as a single reference. We will leave that to future study.

## 7. As can be seen from Figure 3a, no statistically significant genes are found for three cell types, including endothelial, epsilon, mast. How to explain this phenomenon? Will this affect the subsequent deconvolution of the bulk RNA-seq data?

**Our response:**

All these three cell types have a very low number of cells (Endothelial - 16, Epsilon - 7, Mast - 7), and the absence of statistically significant genes suggests that the model properly expresses uncertainty when non-conclusive observations are present. Indeed, we observe a positive correlation between the max gene-by-CTS-topic probabilities and the number of cells for each cell type in the training data (**Figure R15**). Because of that, those 3 cell types do not contribute much during the deconvolution and as a result, they exhibit low cell-type proportions across all cells (**Figure R16**). This ensures no spurious false positives.

**Figure R15.** **Evaluating topic confidence in terms of number of cells per cell type.** We evaluated topic confidence by correlating the topic confidence scores with the number of cells for each cell type available for training. The topic confidence scores were calculated by first taking the maximum probability from the G-by-1 column vector for each cell type and then averaging over all K cell types. The resulting topic confidence scores were plotted as a function of number of cells per cell type. The figure shows the correlation for modeling using 5 topics per cell type for two pancreatic datasets, Segerstolpe and Baron, generated using different approaches, Smart-seq2 and Drop-seq, respectively. A similar plot for modeling using the different number of topics per cell type is shown in **Additional file 1**: **Figure S19**.

**Figure R16.** The figure shows the genes-by-CTS matrix for the top20 genes per topic, depicting the lower probabilities for the endothelial, epsilon, mast cell types (panel a). The uncertainty in modeling these cell types captured as lower gene-by-CTS topic probabilities ensures that they are deconvolved with lower proportions in all samples, across different bulk datasets (panel c and e), ensuring no spurious false positives. This figure is a part of **Fig. 3** in the main text.

## 8. The authors need to carefully check the writing of mathematical formulas and give definitions of all symbols involved. For example, the authors define n_(m,.,k)^(-(i,m) ) and n_(.,g,k)^(-(i,m) ) to be the total number of scRNA-seq reads allocated for topic k for cell m and the total read counts for gene g under topic k across all of the M cells without counting the current i^th read for cell m, however, according to the formulas written by the authors, the former excludes all reads from gene g and the latter excludes all reads from cell m.

**Our response:**

Thank you for the comment. That was a typo. You are right -(i,m) implies total count except for read i in cell m. We apologize for that. We have amended the formula and also substantially improved the clarity of the method description.

Please refer to the subsection “Modeling scRNA-seq reference data via topic inference guided by cell-type labels” under **Methods**.

# Reviewer #3:

**Adapted from their prior work MixEHR-Guided, Swapna et al. proposed a guided topic model for cellular deconvolution (GTM-decon). Using single-cell RNA-seq and guided by the cell cluster labels, they estimated a cell type-specific gene expression profile matrix and used it to estimate cellular fractions from bulk gene expression data. While they did a lot of data analyses, the method was not sufficiently presented, and thus it's hard to understand how the cellular fractions were exactly estimated. The method is also not rigorous and comprehensively benchmarked. Below I list some comments that may improve the manuscript.**

**My main concern is about the clarity of the methods.**

## 1. The notations in Fig 1a do not match that on Page 12. In Fig 1a, there is X_{i,m}, where i denotes the scRNA-seq read count. On page 12, there is only x_{g,m}, where g stands for a gene.

**In Fig 1a, there is \beta_k, while on page 12, "a fixed hyperparameter across all K dimensions" was used.**

**In Fig 1a, there is prior + 0.1, which is not described in Methods.**

**In Fig 1a, "n = 1, …, Nj; j = 1, …, D." Those notations are not used in Methods. A clear description of the methods with precise notations is needed.**

**Our response:**

Thank you for the meticulous attention. You are exactly right in pointing out the typos in all of those notations. We apologize for that.

On page 12, $x_{g,m}$ should have been $x_{i,m}$ as it represents the index of the gene mapped by read i in cell m.

In **Fig. 1a**, $\beta_{k}$ should have been $\beta$ for fixed hyperparameter of $\phi_{k}$.

In **Fig. 1a**, you are right that the baseline value of 0.1 is added to all of the $\alpha_{m,k}$ values and it should have been described in **Methods**. We added this description to the “GTM-decon model overview” subsection under **Results**: “*It follows a K-dimensional asymmetric Dirichlet distribution* $\theta_{m}\sim Dir(\alpha_{m}+0.1)$” and to the “GTM-decon overall algorithm” subsection under **Methods**: “Specifically, *we cast each topic to exactly one cell type via the means of asymmetric topic prior* $\theta_{m}\sim Dir(\alpha_{m}+0.1)$.”

In **Fig. 1a**, the indices for reads and cells should have been i = 1, …, N_m_ and m = 1, …, M, respectively, where N_m_ is the total number of reads in cell m and M is the total number of cells.

We have not only fixed all of the above typos and omissions in the revision but also substantially improved the clarity of the method description throughout the manuscript.

## 2. In methods, "Given topic _{i,m} = , we then draw a gene index _{g,m} from a G-dimensional categorical distribution: x_{g,m} ~(_k), where _ follows a G-dimensional Dirichlet distribution with a fixed hyperparameter across all K dimensions: _k~()." Can you explain how z_{i.m} plays a role in x_{g,m}? Aren't the read counts mapped to each gene?

**Our response:**

The latent variable $z_{i,m}\in\{1,...,K\}$ determines which cell-type-specific (CTS) topic read *i* is originated from. Given $z_{i,m}=k$, we then sample a gene indexed by $x_{i,m}$ from the corresponding CTS topic distribution $x_{i,m}\sim Cat(\phi_{k})$. Once again, as the Reviewer pointed out, $x_{g,m}$ should have been $x_{i,m}$. We apologize for the typo that caused the confusion.

## 3. I think the main confusion comes from the fact that there is no idea where the observed scRNA-seq comes into the posterior solution shown on page 13. All seem to be parameters.

**Our response:**

The posterior topic inference is conditioned on the gene index recorded in the observed variable $x_{i,m}$, where we denote as $g$, i.e., $x_{i,m}=g$ without loss of generality. In deriving the topic inference algorithm, we followed the derivation by Minka (2000) and Griffiths and Steyvers (2004) (referenced below) by first integrating out the Dirichlet variables $\theta$ and $\phi$ because their Dirichlet priors are the conjugate to the Multinomial variables z and x, respectively.

Specifically, in the conditional probability of topic assignment, the first term is proportional to $p(z|\alpha)$ after integrating out $\theta$ (i.e., $p(z|\alpha) = \int p(\theta|\alpha)p(z|\theta) d\theta$) whereas the second term is proportional to *the data likelihood* $p(x|z)$ after integrating out the Dirichlet variables $\phi$ (i.e., $p(x|z) = \int p(x|\phi,z)p(\phi|\beta) d\phi$):

$$p(z_{i,m}=k |x_{i,m}=g, x^{-(i,m)}{, z}^{-(i,m)}) \propto p(z_{i,m}=k{, z}^{-(i,m)}|\alpha_{m,k}) p(x_{i,m}=g, x^{-(i,m)}|z)$$

$$\propto(\alpha_{m,k}+{n_{.,m,k}^{(-i)}})(\frac{\beta+{n_{g,.,k}^{-(i,m)}}}{G\beta+\sum_{g} n_{g,.,k}^{-(i,m)}})$$

Here ${n_{g,.,k}^{-(i,m)}}$= ${n_{x_{i,m},.,k}^{-(i,m)}}$ is the data evidence from the observed scRNA-seq. The above inference can be done by Gibbs sampling for the topic assignment for each read *i* and cell *m* while fixing the rest of the topic assignments $z^{-(i,m)}$. For a large number of cells in the scRNA-seq data, the collapsed Gibbs sampling approach tends to be slow in reaching equilibrium state. Therefore, we took a deterministic mean-field variational inference approach, known as the collapsed variational Bayesian (Teh et al., NeurIPS 2006).

The variational distribution $\gamma_{i,m,k}$ presented in page 13 in the original manuscript has the same expression except using the variational expectation $\gamma^{-(i,m)}$ to compute the sufficient statistics ${n^{(-i)}}_{.,m,k}=\sum_{i'\neq i} \gamma_{i',m,k}$ and ${n^{-(i,m)}}_{g,.,k}=\sum_{m'\neq m or i'\neq i} [x_{i',m'}=g]\alpha_{m,k}\gamma_{i',m',k}$.

Please refer to the revised text for a more detailed description in the subsection “Modeling scRNA-seq reference data via topic inference guided by cell-type labels” under the **Methods**.

**References**:

1. T Minka. Estimating a Dirichlet distribution. Technical report, MIT, 2000.
2. Thomas L Griffiths and Mark Steyvers. Finding scientific topics. *Proceedings of the National Academy of Sciences of the United States of America*, 101 Suppl 1(Supplement 1):5228–5235, April 2004.
3. Teh, Y. W., Newman, D. & Welling, M. A collapsed variational Bayesian inference algorithm for latent Dirichlet allocation. Advances in Neural Information Processing Systems (2006).

## 4. How sensitive are the results to the hyperparameters? E.g., 0.9 was used for a cell assigned to a cell type. Why use 0.9? How does that differ from using 1? If to account for cluster uncertainty, some clustering methods have estimated those multi-type probabilities (e.g., using Dirichlet distribution). Using that estimated information may be more accurate than those arbitrary probabilities of 0.9 and others sampled uniformly from 0.01 and 0.1.

**Our response:**

We thank the Reviewer for raising this important point. We experimented with varying the values of the priors from 0.6 to 1. We also derived a data-driven prior from a multi-class logistic regression (MCLR) model that was trained to predict cell types of each cell. Specifically, from the fitted MCLR model, we use its fitted values in terms of the predicted probabilities as the prior values for each topic. The fitted values for all the correct cell types are above 0.8, suggesting a high confidence of the MCLR fit.

We experimented these prior settings on several datasets:

a) On two bulk RNA-seq datasets of cell-sorted purified immune cells, GSE107011 (114 samples) and GSE64655 (48 samples), we evaluated the effect of varying topic prior as deconvolution accuracy (**Figure S4**). **Figure R17** shows the deconvolution accuracy derived from two different scRNA-seq datasets as reference, and highlights that the accuracy is largely similar while varying the priors from 0.6-1, as well as while using priors derived from mult-class logistic regression. **Figure S4** shows that the results are similar even while using three different gene sets as features - all genes, preprocessed genes, highly variable genes. Since the prior of 0.9 also enables the modeling of uncertainty, we choose to continue with this prior as the default. Visualizing the results as heatmaps confirms that varying the topics recovers a similar pattern of clustered samples, largely clustered based on their primary cell type (**Figure S5**).

b) On a bulk RNA-seq dataset of whole blood samples of 12 patients with ground truth cell proportions determined by flow cytometry, we evaluated the effect of varying topic prior on deconvolution by Pearson correlation between ground truth cell type proportions and the corresponding deconvolved proportions by GTM-decon (**Figure R18**).

These results show that GTM-decon is robust to the different prior values assigned as long as the matching CTS topic prior values are greater than values for the rest of the topics (i.e., 0.01-0.1).

**Figure R17**. **Effect of varying CTS topic prior on deconvolution accuracy in purified immune bulk RNA-seq samples for GSE107011, GSE64655.** For each purified bulk sample, the cell type corresponding to the highest inferred cell-type proportion by GTM-decon (1 topic per cell type) was used as the predicted cell type. The barplots show the prediction accuracy as the percentage of the correctly predicted samples as a function of topic prior values from 0.6 to 1, as well as topic priors derived from a multi-class logistic regression (MCLR) model fitting the scRNA-seq data to its cell types (1topic_MCLR), for all the gene sets evaluated in the study. The bar plots reveal that the method is robust to variation in topic prior. Plots for the different gene selection strategies are shown in **Figure S4**.

**Figure R18 (Figure S6). Effect of varying CTS topic prior on deconvolution performance in real bulk datasets with ground truth proportions.** We evaluated the variation in two bulk RNA-seq datasets with ground truth proportions – Whole Blood and GSE107011. The box plots show the deconvolution accuracy in terms of Pearson Correlation between ground truth cell type proportions estimated by flow cytometry for each sample vs. deconvolved cell type proportions inferred by GTM-decon (y-axis). The box and the whiskers in each boxplot indicate the 25%-75% quartile and min-max of the evaluation scores for each of the samples. Deconvolution performance is shown as a variation of topic prior from 0.6 – 1. The bar plots reveal that the method is largely robust to variation in topic prior, with all genes and highly variable genes performing better than pre-processed genes.

## 5. While the paper's main focus is estimating cellular fractions from bulk data, it was only briefly described on Page 13, following an algorithm similar to assigning single cells to a topic. However, the single-cell algorithm used prior about cell labels in alpha. How was that used in bulk data? A specific algorithm should be described for bulk data without referring to other references.

**Our response:**

We thank the reviewer for raising this important technical point. You are right that for deconvolving the bulk, we typically do not have any prior information about the cell-type mixtures in the bulk RNA-seq data. Therefore, a flat hyperparameter value fixed at a constant value across all K cell types (default: $\alpha_{k}=0.1\forall k$) is used. We elaborated the deconvolution algorithm in the subsection “**Inferring mixing cell-type proportions in bulk transcriptome**” in **Methods**.

## 6. The benchmarking is short, on a paragraph on page 4. In Fig 2a, the authors calculated the correlation for each bulk sample across cell types. It is more important to calculate the correlation across samples for each cell type, and also assess the bias. The simulation is a simple combination of single-cell data and would be optimistic. Real data benchmarking is more useful, e.g., Framingham Heart Study collected white blood cell counts and gene expression data and can be used as a good real benchmarking dataset.

Thank you for this excellent point. To further establish the deconvolution accuracy of our method, we have undertaken benchmarking on 7 real bulk datasets from 3 tissues for which the groundtruth cell-type proportions are available (**Additional file 1**: **Table S2**):

A. Three bulk RNA-seq datasets of peripheral blood mononuclear cells (Whole Blood, PBMC S13 cohort, SDY67), with ground truth proportions calculated from flow cytometry
B. Two bulk RNA-seq datasets of cell-sorted purified immune cells (GSE107011, GSE6465)
C. Bulk RNA-seq samples from human brain tissue of a subsample from the Religious Orders Study and Memory and Aging Project (ROSMAP) study, for which ground-truth cell composition information is available from immunohistochemistry.
D. A bulk RNA-seq dataset from pancreatic tissue for 7 individuals, whose scRNA-seq gene expression profiles are also available (E-MTAB-5060 and E-MTAB-5061). The cell-type proportions in the scRNA-seq are used as a surrogate for cell-type proportions in bulk.

We also thank the reviewer for referring us to the Framingham Heart Study. The dataset is not public and also is a microarray dataset whereas our main focus is on deconvolving RNA-seq data.

We compared the performance of GTM-decon using three gene selection strategies (ALL, PP, HVG) on these datasets with other existing deconvolution methods - MUSIC, CIBERSORTx, BSEQ-sc, Bisque (**Figure R19**, also **Figure 2** in main text, **Table R5**). We evaluated deconvolution performance on sample deconvolution using Spearman R and Root Mean Square Error (RMSE), by comparing the inferred proportions for the matching cell types against the ground truth proportions for each sample. GTM decon conferred on par or superior performance compared to the existing methods for all the datasets (**Figure R19a**, also **Fig 2a** in main text).

We also undertook a complementary analysis - by evaluating deconvolution performance on individual cell types (**Figure R19b**, also **Fig 2b** in main text). We observe that GTM-decon performs distinctly better than the other methods for deconvolving each cell type in the PBMC bulk samples (SDY67) (**Figure R19b**, also **Fig 2b** in main text), and comparably for other datasets (**Figure S11 - S15** in **Additional file 1**).

Additionally, we evaluated the deconvolution accuracy for purified bulk RNA seq data of immune cells (GEO accession numbers: GSE107011 and GSE64655; **Table S2**) using two different independent references HBC and PBMC2 (**Table S1**). Overlapping cell types were used to evaluate the performance on the purified bulk samples , whereby the highest deconvolution proportion was used as the predicted cell type label for computing the prediction accuracy. GTM HVG achieved the highest accuracy across all four experiments (**Figure R19c**, also **Fig. 2c** in main text). Detailed explanation of the results is provided in the section “***Evaluation of deconvolution of real bulk with ground truth cell type proportions***” under **Results**.

**Table R5.** Spearman correlation between the ground-truth and inferred cell-type proportions on real bulk samples.

| **Method** | **Ref: PBMC2, Bulk: SDY67** | **Ref: PBMC2, Bulk: S13 cohort** | **Ref: HBC, Bulk: CBx Whole Blood** | **Ref: Lake, Bulk: ROSMAP** | **Segerstolpe Real Bulk LOOCV** |
| --- | --- | --- | --- | --- | --- |
| **GTM-All** | **0.742557** | **0.692460** | 0.366667 | **0.831707** | 0.570884 |
| **GTM-PP** | 0.704029 | 0.535714 | 0.391667 | 0.802439 | 0.786839 |
| **GTM-HVG** | 0.647980 | 0.386905 | **0.875000** | 0.109756 | **0.859543** |
| **BSEQ-sc** | 0.057754 | 0.202781 | 0.400000 | 0.501448 | 0.763868 |
| **CIBERSORTx** | -0.041038 | 0.357156 | -0.278897 | 0.827131 | 0.758431 |
| **MuSiC** | 0.309026 | 0.193214 | 0.862283 | 0.762179 | 0.801137 |
| **BISQUE** | N/A | N/A | -0.116667 | N/A | 0.583751 |

**Figure R19** (**Fig. 2** in the main text). Evaluation of deconvolution performance on real bulk data. Please refer to figure legend for Figure 2 for detailed description.

## 7. In "Preprocessing the reference scRNA-seq data," "all: simple count data preprocessed to remove outliers (highly expressed genes -found in ≥ 80% of cells, lowly expressed genes - found in ≤ 5 cells)." Did you filter out genes based on the percentage of nonzero expression? How many genes are filtered out? It seems improper to call "all" genes, given some genes are filtered out.

**Our response:**

We agree that the terminology of “all genes” is misleading. We apologize for the confusion. In our original manuscript, we had used gene expression profiles from a leniently pre-processed gene set, which excludes likely housekeeping genes (i.e., genes that are expressed in >=80% cells) as well as infrequently expressed genes (i.e., genes that are expressed in < 5 cells). We now refer to this set as **pre-processed (PP) genes** in the manuscript. These genes correspond to ~60% to ~80% of all genes, depending on the datasets (**Table R6, Table S3** in **Supplementary Information**). In the revised version, along with the PP genes, we also consider *the entire set of genes* (without any pre-filtering) in the scRNA-seq reference data, and refer to that as the “**all genes**” set in the manuscript. We have modified the text in the “**Preprocessing the reference scRNA-seq data**” section under the **Methods** section to elaborate on the different gene sets and their transformations.

**Table R6** (**Table S3**): Summary of gene selection for scRNA-seq datasets

| **Dataset** | **Total genes** | **PP genes** | **HVG** | **%PP genes** | **%HVG** |
| --- | --- | --- | --- | --- | --- |
| **Segerstolpe** | **26178** | **17486** | **5027** | **66.8** | **19.2** |
| **Baron** | **20130** | **15502** | **1667** | **77** | **8.28** |
| **Breast – Normal** | **33694** | **20721** | **2060** | **61.5** | **6.11** |
| **Breast - Cancer** | **29736** | **26169** | **2355** | **88** | **7.92** |
| **PBMC2** | **33693** | **20830** | **8225** | **61.8** | **24.4** |
| **HBC** | **19815** | **17577** | **4389** | **88.7** | **22.1** |

## 8. Software computation time and memory requirements need to be discussed.

**Our response:**

Thank you for this comment. We have benchmarked the time cost and memory usage as a function of the number of cells in the scRNA-seq reference data for GTM-decon in comparison with 3 SOTA methods, namely BISQUE, BSEQSC, and MuSiC. We did not benchmark CIBERSORTx because it is run remotely on a hosted web server. For the scRNA-seq reference, we used a combined PMBC datasets (Additional file 1: **Table S2**) containing 30,000 cells and 33,694 genes. Each method was trained to infer the cell type proportion in the purified bulk samples (GEO accession GSE64655). While the exact type of bulk data is not important in these time/memory benchmark experiments, we expect that each algorithm converges relatively fast due to the homogeneity of the bulk data. For GTM-decon, we used 1-5 topics per celltype (i.e., 9-45 topics in total for 9 cell types in the scRNA-seq reference data). For the other 3 methods, we used their recommended or default settings. We used the GNU time command to record the time and memory usage. GTM-decon scales linearly with both the number of topics per cell type and the number of cells (**Figure R20** top row), which is what we expected since its time and space complexity are both for N cells, G genes, and K topics. For a large number of cells, we can also perform stochastic variational inference to rapidly update model parameters based on mini-batches of cells with much lower memory overhead. It also compares favorably with BISQUE, BSEQ-sc, and MuSiC in terms of running time and memory usage (**Figure R20** bottom row).

**Figure R20.** We benchmarked the time and memory behavior or GTM-decon as a function of both input reference size and number of topics per celltype. We trained on 1e3, 2e3, 4e3, 8e3, 1.6e4, 3e4 cells, with 33,694 genes and deconvolved the same purified bulk PBMC data (GSE64655). BISQUE, BSEQ-sc, and MuSiC were all run with their default settings. All methods were run on a server with 62Gb RAM, 29Gb swap, with 20 Intel Xeon CPU e5-2650 v4s at 2.20Ghz. GTM-decon time and memory cost increase with the number of topics, but not exponentially, and GTM-decon at 1 topic per cell type has the lowest max memory usage and comparable run times to BSEQ-sc and BISQUE, and GTM-decon with 5 topics per celltype still achieving much higher speed than MuSiC, the slowest model. This figure is **Additional file 1: Figure S17**.

## 9. In Discussion, "Our results suggests that most of the top genes under the cell-guided topics correspond to known marker genes (Figure 3a, Figure 5a)." I cannot see that most top genes correspond to known marker genes from the two figures. It is also unclear why there are NA in marker legends. In the two marker databases, a gene is either claimed to be a marker or not, right?

**Our response:**

We thank the reviewer for bringing this valid point. For the cell types for which we do have marker gene information, we observe that the majority of the top genes are known marker genes (i.e., **Figure 3a**, **Figure S24a**). Therefore, we revise the sentence in the manuscript as follows:

“Through our analysis of *the pancreatic and breast tissue datasets, we observe that for those cell types where marker gene information is available, most of the top genes under the cell-guided topics correspond to known marker genes (****Fig. 3a****; Additional file 1:* ***Figure S24a****).*”

Regarding the usage of “NA”, this term is used to indicate cell types where marker gene information is not available in those databases, and hence it is not possible to evaluate whether the top genes are markers or not. We clarified this further in the figure legend for **Fig. 3a** and **Figure S24a**:

“*Whenever available from CellMarkerDB and PanglaoDB, cell-type marker genes are indicated on the left. For the cell types, where marker genes are not available, “NA” were indicated on the left.*”

## 10. The authors need a more thorough literature review. For example, in the Discussion, "The latter allows us to identify cell-type-specific differential expression of genes directly from bulk RNA-seq data (Figure 9). This feature is not available in most approaches developed for cell-type deconvolution, except CIBERSORTx..”. Several other methods can also do this, like CellDMC, TOAST, and bMIND.

**Our response:**

Thank you for the references. What we meant was that most methods can either perform deconvolution or cell-type-specific (CTS) differential expression analysis (DEA) but rarely both. For example, in order to estimate CTS gene expression from the bulk samples, both TOAST and bMIND need an external deconvolution method (i.e., TOAST used *deconf* by default whereas bMIND used Cibersort NNLS or Bisque by default) to first estimate the cell-type proportion from the bulk. Therefore, their accuracy hinges on the accuracy of the external deconvolution methods. Conversely, most common deconvolution methods are not able to estimate CTS gene expression themselves. Therefore, our GTM-decon is a unified framework that performs both deconvolution and CTS-DEA in bulk samples.

That being said, we can still draw salient conceptual differences when comparing GTM-decon with TOAST and bMIND.

While our GTM-decon is also a reference-free method as TOAST, it leverages the much richer single-cell transcriptome data by modeling its distribution via our guided topic model algorithm. In contrast, TOAST relies on purified bulk samples as the reference. GTM-decon properly expresses uncertainty in the inference of cell-type-specific marker genes. Unlike TOAST, GTM-decon does not use arbitrary threshold to choose the feature list (i.e., step 2 in the TOAST algorithm) but rather infers the CTS relative gene expression distribution in the form of Dirichlet topic distribution. Moreover, GTM-decon can infer sub-cell-type distribution by dedicating more than 1 topic per known cell type. We are also aware of a more recently developed method called CeDAR (Chen et al., *Genome Biology* 2023) that uses known cell-type hierarchy as prior to infer cell-type-specific expression in bulk data as opposed to our de-novo sub-cell-type inference and will leave a more detailed comparison with CeDAR as future work.

Similar to TOAST, bMIND does not model scRNA-seq distribution but rather uses an estimated CTS expression as the mean of the CTS expression Gaussian variable, whose posterior distribution is being inferred from the target bulk sample. bMIND also treats each gene separately (as shown in Equation 1 of the paper) by assuming all genes are independent. In contrast, GTM-decon models all genes simultaneously and properly express uncertainty in inference of the CTS gene expression.

We briefly discussed TOAST and bMIND in the **Introduction** and CeDAR in the **Discussion**. Since CellDMC was designed for differential methylation analysis and also bMIND was compared with CellDMC and showed better performance, we will leave more systematic comparison with CellDMC as potential future direction as well.

## 11. The tables in Fig 1 need to be improved. All unassigned probabilities = 0.05.

**Our response:**

We thank the reviewer for bringing this to our notice. We have modified the cartoon table in **Fig. 1** to reflect the random probabilities assigned by the method.

## 12. The paper is too long, and some figures can be condensed and summarized. For instance, the heatmaps of cellular fractions in Fig 2 are hard to compare. Using scatterplots by cell type would make it clearer.

**Our response:**

Thank you for the suggestion. We moved Figures 2, 4, 5, and 6 in the original Manuscript to **Additional file 1** (i.e., supplementary information) and removed the subsections in Results and Methods on the deconvolution analysis of combining two external scRNA-seq references for deconvolution due to the use of a better matched tumor single-cell reference data. We added the scatterplots to **Figure 2** along with evaluation on real bulk deconvolution. While we added a few more highlighted paragraphs on our methodological contributions and clarified the method description, we hope that the paper is now a lot more succinct in terms of its take-home message as a Method paper. We can also move more sections to **Additional file 1** if needed.

## 13. The paper needs more editing. A quick reading would find the following issues.

**Page 4, second line, "tends provided"**

**Page 5, last line, "into into"**

**Page 10, line 25, "results suggests"**

**Page 12, second line, ", ."**

**Some references are duplicated. Ref 21 = 25; Ref 41 = 48.**

**On p. 8 when talking about the hyperparameters it is not clear at that point what is meant by the "hyperparameters" (unless you have read the supplement).**

**Our response:**

We thank the reviewer for pointing out the edits. We have addressed each of the mentioned edits, and also proofread the entire manuscript.

**2^nd^ round**

**Reviewer 2**

The authors have responded to my comments and I have no further concerns.

**Reviewer 3**

The manuscript has been substantially improved, and the method description is clear now. Below I list some remaining questions.

In the "Learning phenotype-specific gene signatures from bulk RNA-seq data" section, more justification is needed to interpret the "sparsifying" process. It seems like this sparsification is just to suit the topic model better. However, what does the sparsified bulk data mean? Would GTM-decon fail for dense bulk data or performs worse than sparsified bulk data?

The rationale for sparsifying bulk data is based on the (75%) zero expression in single-cell data. It makes sense to have zero in single-cell data, but when bulk data measure many cells, the bulk expression should be dense, not sparse.

Would sparsifying make a lot of genes with all zero expression in bulk data, and thus they do not contribute to the deconvolution?

Also, this sparsifying applies to both cellular deconvolution and phenotype/disease-subtype deconvolution. Putting the description of sparsifying only in the phenotype section may be misleading as it only applies to phenotype-subtype deconvolution.

Would the downstream CTS DE analysis lose many genes because of sparsifying?

Some deconvolution methods considered cell size since larger cell types produce more RNA molecules and thus would overestimate cell fractions as RNA fractions. I noticed that cell size is not discussed. Are the estimated fractions RNA fractions or cell fractions?

It is great that multiple existing methods have been compared in real data benchmarking. Can you compare GTM-decon with BayesPrism as well? As discussed in the paper, BayesPrism also uses a topic model.

In Fig 2a, the correlation is calculated across cell types per sample. As I commented in the first review, this is over-optimistic since it is easy to maintain the relative fractions within a sample, but this is not directly related to cross-sample analysis. Instead, reporting the cross-sample correlation for each cell type is more reasonable. For instance, the differential fraction analysis comparing cases and controls directly uses this information. So I suggest reporting this in Fig 2a by aggregating and moving those CTS correlations in the supplement to the main figure.

Also, should the R2 in Fig 2b R since there are negative values?

"There are few reference-free methods like ours that can infer de novo (sub-)cell-type-specific gene expression distributions over all genes from a single-cell reference data distribution without marker gene information."

The commonly used definition of reference-free deconvolution is not based on using marker genes not. MuSiC does not use marker genes but is still a reference-based method. Reference-free deconvolution instead does not use the cell type-specific (CTS) gene expression signature/profile, like TOAST which estimates reference as shown in its Fig 1. The proposed GTM-decon uses the genes by CTS topics estimates inferred from the scRNA-seq reference data, so it should be defined as a reference-based method. The term "reference" has been used in the text. E.g., "We evaluated the deconvolution accuracy for purified bulk RNA-seq data of immune cells using two different independent *references* (HBC and PBMC2)."

Minor comments:

Ref 33 is invalid. Ref 62 lacks information.

Some figures in Fig 3b need to be bigger.

Fig 4a, the 3D pie chart is notorious since the areas do not look proportional to the real proportions. A 2D pie chart works.

**Authors’ response**

We would like to thank all reviewers once again for the thoughtful and constructive comments. We have made further revision to address Reviewer 3’s comments. In summary,

1. We clarified that the sparsification procedure was done on the bulk data only when we directly inferred gene-topic distributions from them, which pertains to inferring *de novo* topics from the bulk data by LDA, the phenotype-guided topic inference from the bulk, and nest-guided topic inference. All of the deconvolution experiments, where we first inferred gene-topic distributions from single-cell data and then applied the inferred topics to deconvolve bulk data, does not involve sparsification (i.e., deconvolving the original bulk transcriptomes as they are). We added the subsection “**Sparsification of the bulk data to directly infer topic distributions from them**” under Methods to clarify it.
2. We also experimented the effect of sparsification by observing the phenotype prediction accuracy as a function of the sparsification rates between 0 to 90 (**Figure R1**) and by examining the rank distribution of the genes that were removed from the bulk samples due to 75-percentile sparsification (**Figure** **R2**). The details were presented in our response to Reviewer 3’s comment 1 and added to **Additional file 1 S7**.
3. To further demonstrate the phenotype-guided and phenotype+cell-type guided functionality without any sparsification on the bulk data, we applied GTM-decon to the **scRNA-seq data** from breast cancer tumors using both the **phenotypes (i.e., ER+ and TNBC)** and **cell-type labels** as guides and then used the inferred topics to deconvolve the original bulk TCGA breast tumor transcriptome data. Details were presented in our response to Reviewer 3’s comment 1 and added to **Additional file 1 S8**.
4. We added a new **Fig. 2b** that displays the overall deconvolution accuracy per cell type for each method and the five real bulk datasets.
5. We evaluated BayesPrism on real bulk deconvolution and updated all the figures accordingly.
6. We show a strong correlation between cell fraction and RNA fraction across cell types based on the four single-cell reference datasets to show that the RNA fractions per cell type can serve as a good surrogate to the cell fraction per cell type despite the potential differences in cell sizes among cell types (i.e., different average RNA counts among cell types) (**Figure** **R5**, **R6**).

The revised contents were highlighted in the main text with track changes.

# Reviewer #2:

**The authors have responded to my comments and I have no further concerns.
Our response:** Thank you once again for the constructive feedback. They helped a lot in improving our manuscript.

# Reviewer #3:

**The manuscript has been substantially improved, and the method description is clear now. Below I list some remaining questions.**

**Our response:** We are glad to hear that and thank you once again for the constructive feedback. They helped a lot in improving our manuscript.

## 1. In the "Learning phenotype-specific gene signatures from bulk RNA-seq data" section, more justification is needed to interpret the "sparsifying" process. It seems like this sparsification is just to suit the topic model better. However, what does the sparsified bulk data mean? Would GTM-decon fail for dense bulk data or performs worse than sparsified bulk data?

## The rationale for sparsifying bulk data is based on the (75%) zero expression in single-cell data. It makes sense to have zero in single-cell data, but when bulk data measure many cells, the bulk expression should be dense, not sparse. Would sparsifying make a lot of genes with all zero expression in bulk data, and thus they do not contribute to the deconvolution?

## Also, this sparsifying applies to both cellular deconvolution and phenotype/disease-subtype deconvolution. Putting the description of sparsifying only in the phenotype section may be misleading as it only applies to phenotype-subtype deconvolution. Would the downstream CTS DE analysis lose many genes because of sparsifying?

**Our response:**

We apologize for the confusion. First of all, we would like to clarify that the sparsification procedure was done on the bulk data only when we directly inferred gene-topic distributions from them (i.e., only when we trained GTM-decon directly on the bulk data). This only pertains to inferring *de novo* topics from the bulk data using the standard LDA and the phenotype-guided topic inference from the bulk data. All of the deconvolution experiments (where we first inferred gene-topic distributions from single-cell reference data and then deconvolved bulk data) does not involve sparsification of the bulk data. In other words, the general deconvolution process uses the bulk dataset as they are.

The results shown in **Figs.** **2-4**, and described in all 8 Results sections up until “**GTM-decon learns phenotype-guided gene topics specific to BRCA subtypes**” all used the original bulk data (except for the *de novo* topics presented in **Fig. 4**, which were inferred from the 75% sparsified bulk data by the standard LDA model). This was why we described the sparsification only in “Learning phenotype-specific gene signatures from bulk RNA-seq data” under Methods. To better communicate this, we now added a separate subsection titled “**Sparsification of the bulk data to directly infer topic distributions from them**” under Methods.

As mentioned above, we applied the sparsification process when inferring topics directly from the bulk for phenotype-guided topic inference from the bulk RNA-seq data (**Fig. 5**) and nested guided topic modeling, where the phenotypes from the bulk data were used as a guide to fine-tune the CTS topics in order to identify CTS differentially expressed genes directly from the bulk (**Fig. 6**). We observe that sparsification improved phenotype prediction accuracy on breast cancer data (TCGA-BRCA) for predicting ER+ and Basal subtypes compared to using the original bulk data as they are. Specifically, we varied the sparsification rates on the bulk data by setting all values below the n-th percentile to be zero and evaluated the prediction accuracies as a function of the sparsification rate. We observe increasing prediction accuracies for up to 90% as we increased the sparsification rate up to 60% (**Figure R1**).

Please note that we sparsified values not genes. As a result, the percentage of the non-zero genes (i.e., genes with non-zero values in at least one sample after the sparsification) is about 70% even at 75% sparsification, suggesting that the model still contains a sufficient number of genes for capturing the cell types and phenotype specific variations. We investigated the contribution of the zero genes (i.e., genes that were removed after sparsification due to zero value across all samples). We observe that most of the zero genes occur in the lower 50th percentile of the CTS probabilities (**Figure R2**, left panel). Additionally, most of the zero genes do not feature in the top 25th percentile of the DE genes as determined by DESeq2 on the bulk TCGA-BRCA samples at FDR < 0.05 (**Figure R2**, right boxplot). We added this description in **Additional file 1 S7. Effect of sparsification on phenotype classification**.

**Figure R1 (Figure S27 in Additional file 1). Effect of sparsification rate on prediction accuracy of ER+ versus Basal subtype and percentage of non-zero genes.** In order to effectively capture phenotype signatures directly from the bulk RNA-seq data using our topic modeling approach, the TCGA-BRCA RNA-seq data were sparsified to varying degrees by setting the values below the “n-th” percentile to be zero. We varied the percentiles from 10 to 90, with the 75-th percentile representing the value chosen based on scRNA-seq datasets. The model performance was evaluated as the phenotype prediction accuracy comparing the phenotype-specific topic probabilities with the groundtruth ER+ and Basal on the held-out 20% subjects. The percentage of the non-zero genes (i.e. genes which have non-zero counts in at least one sample after the sparsification procedure) was also shown in the blue curve as a function of sparsification rate.

**Figure R2 (Figure S28 in Additional file 1). Marginal contribution of zero genes to the CTS topics and DE analysis.** Left panel: the boxplots show the distribution of rank percentiles of the genes-by-CTS topic probabilities of the zero genes due to 75-percentile sparsification in the entire gene list for the 5 cell types from the scRNA-seq data of breast tissues. The gene with the highest probability for that cell type is set at 100-th percentile rank and the gene with the lowest probability at the 0-th percentile. Right panel: The boxplot shows the distribution of the rank percentiles of adjusted p-values of DE genes identified by DESeq2 from whole bulk. The gene with the lowest p-value was set at 100-th percentile rank and the gene with the highest p-value at the 0-th percentile.

That being said, we sought alternative approaches that do not require sparsifying the bulk data while still producing phenotype-guided topics and CTS-phenotype guided topics. For the former, we trained GTM-decon on an scRNA-seq dataset collected from breast cancer tumor samples, where some of the cell-types like Cancer.Her2, Cancer.Basal, etc were annotated based on their occurrence in those patients. This allowed us to use those cell types as a surrogate phenotype-guide at the single-cell level and then applied the trained GTM-decon to deconvolve the original TCGA-BRCA data. We observed a concordance between the deconvolved cell-type proportions and the PAM50 classified breast cancer subtypes in the bulk samples as shown in **Figure R3a** and discussed in “**Deconvolving breast cancer transcriptomes revealed subtype-specific markers**” of the Results section.

**Figure R3 (Figure S23 in Additional File 1):** **Deconvolving breast cancer bulk transcriptome based on single-cell transcriptomes from breast tumors**. **a**) Inferred cell-type proportions matched the cancer subtypes. We trained GTM-decon on the scRNA-seq breast cancer dataset using the 8 cell types as a guide. Each boxplot shows the distribution of the cell-type proportion for the samples separated by the cancer subtypes as defined by the PAM50 system. P-values based on the Kruskal-Wallis were shown. The subtype that exhibits the highest cell-type proportion in each panel was circled. **b**) Detailed visualization of the deconvolved proportions. The consolidated heatmap illustrates the cell type and de novo topic proportions from GTM-decon and unguided LDA separately trained on the scRNA-seq breast cancer data and TCGA-BRCA data, respectively. These two sets of topics were colored in gray and blue, respectively. The rows represent the 1212 tumor samples, and the columns represent the cell types and de novo topics. The clinical phenotypes were annotated in the left color bar to help interpret the topics. Abbreviations of cell-types: CAFs – cancer associated fibroblasts; myCAF – myofibroblast; iCAF – inflammatory CAF; DCs – dendritic cells; NK – natural killer; NKT – natural killer T cells; PVL – perivascular-like.

For the latter, we sought to infer both phenotype-specific and CTS topics from the same single-cell breast cancer transcriptomes, which are composed of labeled cells from patients with 11 breast cancer patients of ER+ subtype and 10 patients with triple negative breast cancer (TNBC) subtype. From this dataset, we inferred CTS-phenotype topics simultaneously guided by the cancer subtypes (i.e., ER+ and TNBC) and cell types. We then a) applied the GTM-decon to deconvolve bulk TCGA-BRCA RNA-seq samples into to CTS and phenotype-specific profiles; b) identified CTS-specific DE genes between ER+ and TNBC from the single-cell data; c) used the genes-by-CTS topics matrix to visualize the CTS distributions of DE genes identified from the bulk data. The details are described below.

**Figure R4 (Figure S30 in Additional file 1): Phenotype-CTS guided topic inference of ER+ and TNBC breast cancer scRNA-seq data.**

**a**) Briefly, we inferred the phenotype-CTS topics by assigning one topic to each combination of cell type and phenotype. During GTM-decon training, the topic designated for the observed cell type and phenotype is endowed with a topic prior value of 0.9, and the rest of the topics were set at a random value between 0.01 and 0.1. As a result, these priors guide the topic model to infer phenotype-CTS topic distributions. All the genes in the experiment were used as features.

**b**) Phenotype and phenotype-CTS deconvolution of TCGA-BRCA samples (n=1212). In both heatmaps, each row represents a bulk sample. For the left heatmap, the columns indicate the phenotype-specific topics, and for the right heatmap the columns indicate phenotype-CTS topics, where the prefixes ‘E’ and ‘T’ before the cell type names indicate ER+ and TNBC cancer subtype, respectively. Enrichment (solid rectangle) and depletion (dash rectangle) for known cell types for the cancer subtypes were indicated using blue rectangles (for ER+) and orange rectangles (for TNBC), respectively.

**c**) Genes-by-CTS topic proportions for the 4838 statistically significant DE genes at the empirical p-value < 0.05 based on 100,000 permutation tests, out of which 1687 genes overlap with the 5936 DE genes identified from bulk TCGA-BRCA via DESeq2. The rows correspond to DE genes, and the columns to the change of CTS topics for ER+ with respect to TNBC (therefore indicated as columns with prefix ‘E’). The differences for TNBC minus ER+ comparison are the mirror image of this and hence not shown.

**d**) Differential signals of the TCGA-BRCA DE genes from the DESeq2 analysis of the TCGA-BRCA data. We visualized the differential signals of the DE genes in two ways:

(i) Differential CTS topic probabilities. The left heatmap shows the change of the genes-by-CTS topic probabilities in ER+ w.r.t. Basal-like subtype from the single-cell breast cancer transcriptomes for the 5936 DE genes detected by DESeq2 for ER+ cancer subtype w.r.t. Basal-like cancer subtype from the bulk TCGA-BRCA transcriptomes. Among these 5936 DE genes, 1687 genes were among the 4838 DE genes detected by 100,000 permutation tests from the scRNA-seq breast cancer data based on the differences in CTS-topic probabilities between the ER+ and TNBC phenotypes for each cell type. These genes are shown by the row annotations on the left side of the heatmap.

(ii) Differential average gene expression for ER+ subtype w.r.t. TNBC subtype per cell type. The right heatmap shows the average gene expression differences between the ER+ subtype and TNBC subtype calculated directly from the observed single-cell data. For the ease of comparison, the rows were ordered according to the clustering pattern from the left heatmap. Among the 5936 DE genes from DESeq2, 592 genes were among the 1892 DE genes detected by 100,000 permutation tests based on the differences in average gene expression between the ER+ and TNBC phenotypes for each cell type. These genes are shown by the row annotations on the left side of the heatmap.

Briefly, we modeled the phenotype-guided and cell-type-guided topic model by assigning one topic to each cell type per phenotype. During training, the topic corresponding to that cell type of that phenotype is set to a prior value of 0.9, and the rest of the topics (for the other cell types or phenotype) were set at a random value between 0.01 and 0.1 (**Figure R4a**). These priors guide the topic model to learn phenotype-CTS distribution. All the genes in the experiment were used as features.

We described the results presented in **Figure R4b-d** as follows:

1. The trained GTM-decon was evaluated by its classification accuracy in discriminating ER+ phenotypes from Basal-like phenotypes (the closest subtype to the TNBC subtype from the TCGA-BRCA data) in bulk RNA-seq data from TCGA-BRCA (n=1212). The left panel in **Figure R4b** illustrates that the samples corresponding to ER+ subtype are enriched for the ER+ topic. Interestingly, the samples corresponding to Basal-like subtype exhibit 2 different clusters - a cluster of samples showing enrichment for the TNBC topic (enclosed in green rectangle), and another cluster showing similar distributions of ER+ and TNBC topics (enclosed in brown rectangle). The former cluster likely corresponds to TNBC samples clustered into the Basal-like category, and the latter to others in the Basal-like category.
2. We deconvolved the TCGA-BRCA bulk samples **without sparsification**, resulting in a remarkably high phenotype classification accuracy of 92% using all genes, 90% using pre-processed genes, and 93% using highly variable genes. These results indicate that phenotype-guided topic modeling using scRNA-seq data can be effective in deconvolving bulk RNA-seq data as they are.
3. Furthermore, the CTS topics inferred for each phenotype enable us to visualize the deconvolution per sample at the CTS resolution for ER+ and TNBC/Basal-like subtypes (**Figure R4b**, **right panel**). We observe the enrichment of Cancer-Basal topic for TNBC (enclosed in orange rectangles), and enrichment of LumA and LumB topics for ER+ samples, as expected (enclosed in blue rectangles), and in concordance with the results obtained from cell-type guided topic modeling before (**Figure R3** or **Figure S23** in **Additional file 1**).
4. Using the single-cell breast cancer transcriptomes alone, we identified CTS DE genes between the ER+ and TNBC subtypes based on the phenotypic differences in terms of the CTS probabilities per gene (**Figure R4c**). Based on 100,000 permutation tests of randomly shuffling the phenotype labels across cells, we identified 4,838 DE genes for at least one cell type at the empirical p-value < 0.05, 1,687 of which overlapped with the 5,936 DE genes identified from the TCGA-BRCA bulk RNA-seq dataset using DESeq2 at FDR < 0.05 for testing 20,501 protein-coding genes in total. Therefore, the overlap is statistically significant at p-value = 6.86E-25 based on Hypergeometric test. Similarly, as a baseline, we performed 100,000 permutation tests on the average CTS gene expression values derived from the single-cell data to compare the CTS gene expression difference between ER+ and basal-like subtypes. We identified 1892 DE genes for at least one cell type at the empirical p-value < 0.05, 592 of which overlapped with the 5,936 DE genes identified from the TCGA-BRCA bulk RNA-seq dataset using DESeq2 at FDR < 0.05 for testing 20,501 protein-coding genes in total. Therefore, the overlap is not as statistically significant as the above result at p-value = 0.01041539 based on Hypergeometric test.
5. We then visualized the expression difference of the DE genes from the bulk data in terms of the CTS-topic probabilities difference between ER+ and TNBC (**Figure R4d** left heatmap). We observe two distinct clusters of genes as highlighted in the orange and blue boxes, the former down-regulated in the ER+ phenotype topics (and up-regulated in TNBC), and the latter down-regulated in the TNBC phenotype topics (and up-regulated in ER+).
   a. The cluster of genes upregulated in TNBC phenotype (enclosed in orange rectangle) consists of ~1200 genes, predominantly upregulated in the Cancer-Basal, and Cancer-Cycling cell types. Based on over-representation analysis by ClusterProfiler on the MSigDB database, we found that these genes are enriched for E2F_TARGETS, G2M_CHECKPOINT, MYC_TARGETS_V2, and MITOTIC_SPINDLE pathways at FDR < 1.71E-18, 4.71E-11, 2.37E-05, and 1.07E-02, respectively, with an overlap of 58, 46, 17 and 29 genes. For the E2F_TARGETS gene set, some of the DE genes are MCM4, SYNCRIP, STMN1, TK1, SSRP1, CKS1B, EED, HELLS, USP1, and for the MYC_TARGETS_V2 set, some of the DE genes are MCM4, SRM, NDUFAF4, WDR43, WDR74, PLK1, PUS1, NOP2, RCL1, NIP7, SUPV3L1, MRTO4. The associations of these genes with the TNBC phenotype are supported by the literature. For instance, MYC_TARGETS_V1 and MYC_TARGETS_V2 are associated with tumor aggressiveness and poor survival in aggressive subtypes such as TNBC and HER2-positive breast cancer, when compared with ER-positive/HER2-negative tumors (MYC v1 and v2, both *p* < 0.001) **[1]**. Also, the genes encoding cell-cycle-related targets of E2F transcription factors (E2F_TARGETS) are associated with aggressive clinical characteristics, such as TNBC **[2]**. Higher G2M cell cycle pathway activity has been shown to be associated with worse clinico-pathologic features, with the TNBC subtype exhibiting the highest activity **[3]**.
   b. The cluster of genes up-regulated in ER+ cancer subtype (enclosed in blue rectangle) consists of ~1000 genes, predominantly upregulated in the Cancer-LumA, Cancer-LumB, Cancer-Her2 cell types. These genes are over-represented in the Estrogen Response (Early and Late) pathways, with an overlap of 57 and 48 genes, at an FDR of 2.84E-25 and 9.54E-18, respectively. As the name suggests, these pathways correspond to genes up-regulated in response to Estrogen receptor activation, from which the ER+ subtype derives its name. Interestingly, 20 genes involved in Bile Acid Metabolism are also up-regulated at an FDR of 7.34E-03, which may be relevant as elevated bile metabolism is associated with better survival in breast cancer, with ER+ subtype exhibiting the highest bile acid metabolism in comparison to other subtypes **[4]**.
   c. Finally, we visualized the average gene expression in another heatmap (**Figure R4d** right heatmap), which exhibits a similar but much less salient pattern compared to the heatmap derived from the CTS-topic probabilities (**Figure R4d** left heatmap).

Together, these results further demonstrate the utility of GTM-decon in guiding phenotype and cell-type topic inference to identify disease-associated gene modules.

We added the above results as a separate section titled “**Phenotype-CTS topic modeling of single-cell breast cancer transcriptomes for TCGA-BRCA bulk deconvolution**” to **Additional File 1** **S8** and referred to it at the end of the last Results subsection in the main text.

**References**:

1. Schulze, A., Oshi, M., Endo, I., & Takabe, K. (2020). MYC Targets Scores Are Associated with Cancer Aggressiveness and Poor Survival in ER-Positive Primary and Metastatic Breast Cancer. International journal of molecular sciences, 21(21), 8127. <https://doi.org/10.3390/ijms21218127>
2. Oshi, M., Takahashi, H., Tokumaru, Y., Yan, L., Rashid, O. M., Nagahashi, M., Matsuyama, R., Endo, I., & Takabe, K. (2020). The E2F Pathway Score as a Predictive Biomarker of Response to Neoadjuvant Therapy in ER+/HER2- Breast Cancer. Cells, 9(7), 1643. https://doi.org/10.3390/cells9071643
3. Oshi, M., Takahashi, H., Tokumaru, Y., Yan, L., Rashid, O. M., Matsuyama, R., Endo, I., & Takabe, K. (2020). G2M Cell Cycle Pathway Score as a Prognostic Biomarker of Metastasis in Estrogen Receptor (ER)-Positive Breast Cancer. Int J Mol Sci. 21(8): 2921. doi: 10.3390/ijms21082921
4. Wu, R., Yu, I., Tokumaru, Y., Asaoka, M., Oshi, M., Yan, L., Okuda, S., Ishikawa, T., & Takabe, K. (2022). Elevated bile acid metabolism and microbiome are associated with suppressed cell proliferation and better survival in breast cancer. Am J Cancer Res., 12(11): 5271–5285.

## 2. Some deconvolution methods considered cell size since larger cell types produce more RNA molecules and thus would overestimate cell fractions as RNA fractions. I noticed that cell size is not discussed. Are the estimated fractions RNA fractions or cell fractions?

**Our response:** That’s an interesting point. Algorithmically, GTM-decon estimates the RNA fractions per cell type instead of cell fractions. Therefore, we agree with the Reviewer that differences in cell sizes may lead to divergences between RNA fractions and cell fractions. Using the Pancreas reference data as an example, while we observe differences in the average RNA counts per cell among the seven cell types (**Figure R5** left panel), the relative proportion of RNA counts are quite similar to the relative proportion of the cells (**Figure R5** right panel). More generally, using the four scRNA-seq reference datasets that were used for the deconvolution, we observed strong correlations between the total cell fraction and total RNA fraction associated with each given cell-type (**Figure R6**).

**Figure R5 (Figure S31a, b in Additional File 1)**. RNA counts versus cell proportion per cell type from Pancreas single-cell data. Left panel: Average number of RNA Read Counts for each cell-type in the Segerstolpe 7 Patients pancreas single cell data. Right panel: Matching Cell Proportions to total RNA counts for the Segerstolpe 7 Patients.

**Figure R6 (Figure 31c-f in Additional File 1)**. Comparing the proportions of total RNA read counts per cell-type versus the proportions of single cells per cell-type for the 4 distinct single-cell reference datasets used in deconvolving bulk data. The four scatterplots display strong correlation trends between the two quantities.

## 3. It is great that multiple existing methods have been compared in real data benchmarking. Can you compare GTM-decon with BayesPrism as well? As discussed in the paper, BayesPrism also uses a topic model.

**Our response:** Thank you for the suggestion. We ran BayesPrism using its online web portal (<https://www.bayesprism.org/>), using all genes in the same manner as the other methods, and added it to the benchmarking comparisons for both the simulated data (**Figure R8**) and real bulk data (**Figure R9**). While BayesPrism is a strong contender in deconvolving some of the simulated and purified bulk samples (**Figure R9c**), GTM-decon still achieved the overall best deconvolution performance, especially for deconvolving real heterogeneous bulk samples (**Figure R9b**). Therefore, the results suggest the general effectiveness of topic modeling in cell-type deconvolution and the additional benefits conferred by GTM-decon due to its algorithmic innovations.

**Figure R8 (Figure S10a in Additional File 1):** Evaluation of cell-type deconvolution accuracy. We simulated the bulk data by summing up counts per gene for all cells per individual. For unbiased evaluation, we adopted a leave-one-out cross-validation (LOOCV) design. Each deconvolution method was trained on N-1 individuals and deconvolved the bulk transcriptome simulated using the scRNA-seq data from the held-out individual for validation. The inferred cell proportions by each method were evaluated by Spearman correlation and Cross Entropy. The box and the whiskers in each boxplot indicate the 25%-75% quartile and min-max of the evaluation scores over the LOOCV individuals, respectively.

## 4. In Fig 2a, the correlation is calculated across cell types per sample. As I commented in the first review, this is over-optimistic since it is easy to maintain the relative fractions within a sample, but this is not directly related to cross-sample analysis. Instead, reporting the cross-sample correlation for each cell type is more reasonable. For instance, the differential fraction analysis comparing cases and controls directly uses this information. So I suggest reporting this in Fig 2a by aggregating and moving those CTS correlations in the supplement to the main figure. Also, should the R2 in Fig 2b R since there are negative values?

**Our response:** Thank you for the suggestion. We revised **Fig. 2** as shown in **Figure R9**. We think that both the sample-centric and cell-type-centric evaluation are important. The former can be challenging for deconvolving heterogeneous bulk samples with large variation of the cell proportions. It is also a standard benchmark on evaluating deconvolution performance [1,2]. Therefore, we kept **Fig. 2a** in the main figure (with added BayesPrism performance as suggested above).

For the latter, we added **Fig. 2b** as a heatmap to summarize the performance of all methods (including BayesPrism) on the basis of cell-types. Specifically, we computed the cross-sample performance for each cell-type, for each bulk dataset. We reported both the Pearson correlation R and RMSE. We observed that all methods tend to perform better on the more abundant cell-types based on the known bulk-cell fractions (i.e., the top green bar plots in **Figure R9b**). Overall, GTM-decon conferred competitive performance (i.e., the right red bar plots in **Figure R9b**) with the runner up method being BayesPrism.

**Additional file 1** **Figures S11-15** illustrate the detailed scatterplots of cross-sample deconvolution by each method at each cell-type. You are also correct that the reported performance is the Pearson correlation coefficient R instead of the R-squared. We have corrected this in the titles for **Figures S11-15.**

**References**:

1. Cobos, F. A., Alquicira-Hernandez, J., Powell, J. E., Mestdagh, P. & Preter, K. D. Benchmarking of cell type deconvolution pipelines for transcriptomics data. Nat Commun 11, 5650 (2020).
2. Jin, H. & Liu, Z. A benchmark for RNA-seq deconvolution analysis under dynamic testing environments. Genome Biol 22, 102 (2021).

**Figure R9 (Figure 2** in main text**). (a)** Evaluation of sample deconvolution. We evaluated the deconvolution performance of GTM-decon using all genes (GTM-ALL), preprocessed genes (GTM-PP), and highly variable genes (GTM-HVG) with five SOTA methods – CIBERSORTx, MUSIC, BSEQ-sc, BayesPrism, BISQUE. The 3 immune bulk data and the brain data were deconvolved using independent references of a similar tissue, while the pancreas bulk data is deconvolved using single cell reference from the same individuals in a leave-one-out cross-validation (LOOCV) manner. The bulk labeled PBMC-1 corresponds to SDY67 dataset, PBMC-2 corresponds to S13 cohort, Whole Blood to Whole Blood dataset, and Prefrontal cortex to ROSMAP dataset (Additional file 1: Table S2). For each test bulk sample, Spearman Correlation and Root Mean Square Error (RMSE) were computed between its ground-truth and predicted cell-type proportions by each method. The box and whiskers in each boxplot indicate the 25%-75% quartile and min-max of the evaluation scores over all samples in a dataset, respectively. **(b)** Heatmaps comparing the cell-type specific deconvolution performance of GTM-decon against existing methods on 5 different real bulk datasets with known ground truth mixing proportions. The cell-types are ordered from most to least prevalent in the bulk data (green barplots in first row indicate average proportion for each cell-type in the bulk data). The middle row shows the Pearson Correlation Coefficient between the predicted and known cell-type proportions. The lower row shows the Inverse RMSE (higher is better, scaled between 0 - 1), per cell-type per dataset. The barplots on the right shows the average performance over all cell-types for each method. **(c)** Cell-type prediction accuracy of the purified immune bulk RNA-seq samples. The two panels indicate the use of different independent immune references, for the deconvolution of two purified bulk immune datasets (Accession Numbers: GSE107011, GSE64655). For each purified bulk sample, the cell type corresponding to the highest inferred cell-type proportion by each method was used as the predicted cell type. The barplots show the prediction accuracy as the percentage of the correctly predicted samples.

## 5. "There are few reference-free methods like ours that can infer de novo (sub-)cell-type-specific gene expression distributions over all genes from a single-cell reference data distribution without marker gene information." The commonly used definition of reference-free deconvolution is not based on using marker genes not. MuSiC does not use marker genes but is still a reference-based method. Reference-free deconvolution instead does not use the cell type-specific (CTS) gene expression signature/profile, like TOAST which estimates reference as shown in its Fig 1. The proposed GTM-decon uses the genes by CTS topics estimates inferred from the scRNA-seq reference data, so it should be defined as a reference-based method. The term "reference" has been used in the text. E.g., "We evaluated the deconvolution accuracy for purified bulk RNA-seq data of immune cells using two different independent *references* (HBC and PBMC2)."

**Our response:** You are right that the term “reference-free” was misused in this context and should have been “marker-free” instead. We revised the text accordingly. It is true that completely unsupervised approaches like TOAST (which is based on non-negative matrix factorization) or LDA for that matter do not use a reference. However, identifying the latent manifold learned from the matrix factorization method with all of the known cell types is not guaranteed. These methods also require reference profiles for the post-hoc cluster annotation (i.e., assigning cell types to the latent factors) after the deconvolution process. GTM-decon strikes a balance between leveraging the cell-type information and the heterogeneity of the bulk sample by inferring sub-cell-type distributions.

## Minor comments:

## 1. Ref 33 is invalid. Ref 62 lacks information. Our response: Thanks for the correction. Ref 33 was PMID: 34791404 and Ref 62 was a technical report by Thomas Minka with the publisher being the Technical Report, MIT. We fixed both of them.

## 2. Some figures in Fig 3b need to be bigger.

**Our response:** Thanks for the suggestion. We changed **Fig. 3b** to barplots of -log10 p-values from the GSEA results and displaying a leading edge plot for the most enriched subtopic per cell type on the right.

## 3. Fig 4a, the 3D pie chart is notorious since the areas do not look proportional to the real proportions. A 2D pie chart works.

Our response: Thank you. We changed the 3D to a 2D pie chart and moved the labels inside the pie to save space in Fig. 3a.

**3^rd^ round**

**Reviewer 3**

Thanks for the clarification. To ease comparison, Fig 2b can be in the same format of boxplots as Fig 2a to differentiate sample-specific performance and cell type-specific performance evaluation.
